# Glucose‐Modified Viscosity‐Responsive Turn‐On Fluorescence Molecular Rotors for Wash‐Free Live‐Cell Imaging

**DOI:** 10.1002/cbic.70372

**Published:** 2026-05-17

**Authors:** Takashi Kanamori, Yuki Sadai, Kenji Hida, Takayuki Tsuduki, Chihiro Nogi, Hiroya Asami, Shun‐ichiro Ogura, Hideya Yuasa

**Affiliations:** ^1^ School of Life Science and Technology Institute of Science Tokyo Yokohama Japan; ^2^ Department of Chemistry Faculty of Science Gakushuin University Tokyo Japan

**Keywords:** fluorescent probe, GFP fluorophore, glycoconjugate, molecular rotor, turn‐on fluorescence

## Abstract

The overexpression of glucose transporters (GLUTs) in cancer cells represents a promising diagnostic target. While **2‐NBDG**, a fluorescent glucose analog, enables GLUT‐mediated imaging, it suffers from low brightness and requires high concentrations and washing. Herein, we report the development of turn‐on fluorescent probes for GLUTs based on the viscosity‐sensitive fluorophore in GFP for a wash‐free imaging of prostate cancer cell, PC‐3. A series of fluorescent molecular rotors (FMRs) were synthesized by modifying the 2‐methyl‐4‐(*p*‐dimethylaminobenzylidene)‐5‐imidazolinone (**DMAB**) core. Structural modifications, such as cyclic amine substitution and julolidine incorporation, enhanced fluorescence quantum yields (Φ_F_ up to 0.17 in glycerol) and viscosity sensitivity (*χ* up to 0.87). Among them, the julolidine‐containing fluorophore **Julo‐Ph** showed the best performance. The **DMAB**‐based fluorophores were conjugated to glucose or glucosamine to create GLUT‐targeted probes. A glucosamine‐bound conjugate, **GlcN‐Julo‐Ph,** was found to enable a wash‐free bright fluorescence imaging of PC‐3 cancer cells. Inhibition studies and docking simulations strongly suggested a GLUT‐mediated uptake of the fluorophore. **GlcN‐Julo‐Ph** outperformed **2‐NBDG** in emitting a brighter intracellular fluorescence at a lower concentration, notably, under wash‐free conditions. Our findings shed light on the utility of viscosity‐sensitive FMRs for the design of turn‐on imaging probes and offer a promising platform for a rapid, low‐background cancer cell detection.

## Graphical abstract

We developed fluorescent molecular rotors (FMRs) that respond sensitively to intracellular viscosity. Based on the dye scaffold of GFP, we systematically investigated molecular rigidity and surface area, and discovered FMRs with remarkably high viscosity sensitivity. Finally, by conjugating the FMR with glucosamine, we achieved wash‐free cellular imaging in live cells via glucose transporter‐mediated uptake.

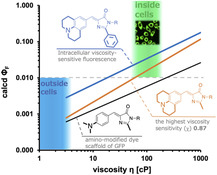

## 1 Introduction

Various imaging modalities targeting tumor‐associated proteins have been developed for cancer diagnosis [1, 2, 3, 4]. Among them, diagnostic strategies that exploit the aberrant glucose metabolism and the overexpression of glucose transporters (GLUTs) in cancer cells—a hallmark of the Warburg effect [5, 6]—are recognized as one of the most widely adopted and powerful approaches [7, 8, 9]. Positron emission tomography (PET) using 2‐[^18^F]fluoro‐2‐deoxy‐D‐glucose (**FDG**) as a tracer, in which the hydroxyl group at the C‐2 position of glucose is replaced with a radioactive fluorine isotope, detects the tracer internalized selectively in cancer cells through GLUTs and enables 3D mapping of cancer tissues in human body [10, 11]. However, PET imaging requires specialized equipment and access to a cyclotron for the timely synthesis of radio‐labeled tracers.

**FIGURE 1:**
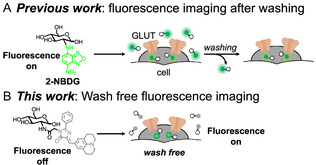
Cell‐imaging protocols with two types of fluorophore‐modified glucose analogs: (A) previously reported **2‐NBDG**; (B) our viscosity‐responsive **GlcN‐Julo‐Ph**.

As an alternative, more accessible imaging modality, the fluorescence imaging with biomolecular‐targeting fluorophores has attracted considerable attention, and numerous fluorescent ligands targeting GLUTs have been developed [12, 13, 14, 15]. For instance, **2‐NBDG** [12, 16], a probe bearing a small fluorophore, 7‐nitro‐2,1,3‐benzoxadiazol, attached at the amino group of glucosamine, has been shown to enter cancer cells via GLUTs. However, due to its inherently low fluorescence intensity, relatively high concentrations (>100 µM) are required for cell imaging [13, 15]. Moreover, in cellular experiments, extensive washing steps are necessary to remove uninternalized **2‐NBDG**, and in vivo applications require a prolonged circulation time of about 1 h for the elimination of obscuring background fluorescence (Figure 1A) [13]. Recently, Tanasova and coworkers reported rhodamine‐based turn‐on glycoconjugates for GLUT‐related imaging, including mannitol‐based probes **ManRho** for GLUT5 monitoring and **GluRho**, a glucopyranoside‐mimetic probe for wash‐free monitoring of GLUT‐mediated glucose uptake [17, 18]. Building on these advances, we became interested in incorporating an additional intracellular activation mechanism into glucose‐conjugated probes. In this study, we aimed to develop a wash‐free, turn‐on fluorescent probe that is nonfluorescent outside cells and becomes highly fluorescent only after cellular uptake via GLUTs (Figure 1B).

**FIGURE 2:**
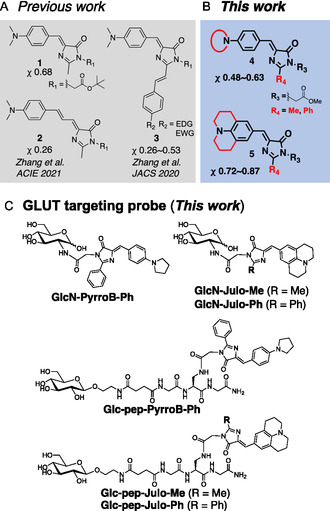
(A) Previously reported **HBDI** derivatives and their viscosity sensitivity values (*χ*), (B) **HDBI** derivatives synthesized in this study and *χ* values, and (C) glucosamine‐ or glucose‐modified **HBDI** derivatives synthesized in this study.

To this end, we focused on fluorescent molecular rotors (FMRs), a class of viscosity‐responsive fluorophores [19, 20, 21, 22]. Some FMRs consist of electron‐donating and ‐withdrawing groups connected by conjugated bonds, and their fluorescence is quenched through the intramolecular rotation in the excited states. These dyes exhibit enhanced fluorescence in viscous or crowded environments because of restricted intramolecular motions. FMR‐based probes have been applied for an intracellular viscosity sensing [22, 23, 24] and a target protein or nucleic acid detection [25, 26, 27, 28, 29]; however, no reports exist of GLUT‐targeted FMRs. Here, we designed a series of glucose‐conjugated FMRs that turn‐on fluorescence only after GLUT‐mediated cellular uptake. The FMR probes must exhibit a strong fluorescence only in the highly viscous environment of the cytoplasm [30, 31]. We thus sought to optimize both the fluorescence quantum yield (Φ_F_) and viscosity sensitivity (*χ* value) of the FMRs under the intracellular viscous conditions. Among known FMRs, the fluorophore of green fluorescent protein (GFP) provides the most promising turn‐on properties as imaginable from the fact that the life crafts GFP by densely wrapping around the intrinsically less bright fluorophore with a polypeptide. Whereas the bare fluorophore, 4′‐hydroxybenzylidene‐2,3‐dimethylimidazolinone (**HBDI**), has a very low Φ_F_ (≈0.0002) [32], GFP exhibits a strikingly high Φ_F_ (≈0.8) [33], suggesting that **HBDI** would bring about a large *χ* value. With the beneficial properties of **HBDI** and its derivatives [34, 35] (Figure 2A) in mind, we developed a series of novel **HBDI** derivatives with a large *χ* and a sufficient Φ_F_ under viscous conditions through the study of structure–photophysics relationships (Figure 2B). These **HBDI** derivatives were then conjugated with glucose derivatives to create novel GLUT‐targeted probes for turn‐on fluorescence imaging of cancer cells, which were evaluated here at the live‐cell level as an initial proof of concept (Figure 2C).

## 2 Results and Discussion

### 2.1 Design and Photophysical Properties

**FIGURE 3:**
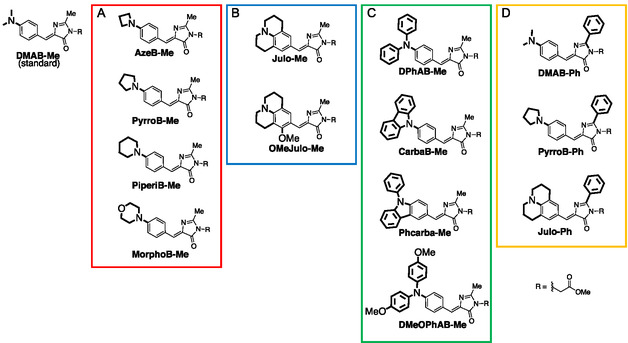
Amino‐modified 4‐(*p*‐dimethylaminobenzylidene)‐5‐imidazolinone (**DMAB**) derivatives synthesized in this study. (A) Cyclic amine modification, (B) julolidine modification, (C) aromatic amine modification, and (D) phenyl modification at the imidazolinone ring.

Intramolecular torsion is a key factor governing the photophysical properties of many small organic fluorophores, as twisting can modulate *π*‐conjugation, excited‐state relaxation, and fluorescence output [34, 35, 36, 37]. In the present HBDI‐derived system, we focused particularly on the rotational freedom of the amino‐group‐containing donor moiety. 2‐Methyl‐4‐(*p*‐dimethylaminobenzylidene)‐5‐imidazolinone (**DMAB‐Me**), an **HBDI** derivative with the hydroxy group replaced with an amino group, has been proposed to undergo nonradiative deactivation in the twisted conformation (the excited state) of its central double bond via the twisted intramolecular charge transfer (TICT) mechanism [34]. Also, an alternative pathway involving twisting of the dimethyl amino group is possible [34, 36]. We hypothesized that suppressing the phenyl–N bond rotation in the excited state by fixation with a crosslinker or by another strategy described below could enhance fluorescence intensity in high‐viscosity environments. In other classes of fluorescent dyes, such as rhodamines and aminocoumarins, replacement of the dimethylamino group with cyclic amines has been shown to increase fluorescence quantum yield [36], which was attributed to a restricted rotation around the C—N bond [38]. Based on these rationales, we designed several structural modifications as shown in Figure 3, in which A) cyclic amines were introduced to reduce rotational freedom of the amino group, B) a rigid julolidine scaffold was incorporated to fully restrict amino group twisting [39, 40] and to further increase molecular surface area, C) aromatic substituents were introduced onto the amino group, and D) a benzene ring was attached at the imidazolinone core. These GFP fluorophore derivatives were synthesized by using previously reported procedures (Scheme S1) [41, 42, 43].

Intramolecular torsion is a key factor governing the photophysical properties of many small organic fluorophores, as twisting can modulate *π*‐conjugation, excited‐state relaxation, and fluorescence output [34, 35, 36, 37]. In the present HBDI‐derived system, we focused particularly on the rotational freedom of the amino‐group‐containing donor moiety. 2‐Methyl‐4‐(*p*‐dimethylaminobenzylidene)‐5‐imidazolinone (**DMAB‐Me**), an **HBDI** derivative with the hydroxy group replaced with an amino group, has been proposed to undergo nonradiative deactivation in the twisted conformation (the excited state) of its central double bond via the twisted intramolecular charge transfer (TICT) mechanism [34]. Also, an alternative pathway involving twisting of the dimethyl amino group is possible [34, 36]. We hypothesized that suppressing the phenyl–N bond rotation in the excited state by fixation with a crosslinker or by another strategy described below could enhance fluorescence intensity in high‐viscosity environments. In other classes of fluorescent dyes, such as rhodamines and aminocoumarins, replacement of the dimethylamino group with cyclic amines has been shown to increase fluorescence quantum yield [36], which was attributed to a restricted rotation around the C—N bond [38]. Based on these rationales, we designed several structural modifications as shown in Figure 3, in which A) cyclic amines were introduced to reduce rotational freedom of the amino group, B) a rigid julolidine scaffold was incorporated to fully restrict amino group twisting [39, 40] and to further increase molecular surface area, C) aromatic substituents were introduced onto the amino group, and D) a benzene ring was attached at the imidazolinone core. These GFP fluorophore derivatives were synthesized by using previously reported procedures (Scheme S1) [41, 42, 43].

**FIGURE 4:**
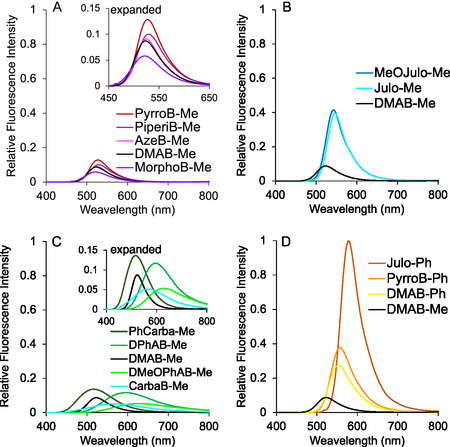
Fluorescence spectra of 15‐μM **DMAB** derivatives in glycerol. (A) Cyclic amine modification, (B) julolidine modification, (C) aromatic amine modification, and (D) phenyl modification at the imidazolinone ring.

To evaluate the viscosity dependence of fluorescence intensity, we measured the fluorescence spectra, as well as the absorption spectra, of the synthesized **DMAB** derivatives in methanol (a low‐viscosity solvent) and glycerol (a high‐viscosity solvent) (Table 1, Figure 4, S1). All derivatives exhibited very weak fluorescence in methanol, with fluorescence quantum yields (Φ_F_) below 0.01 (Table 1, Figure S1(I–L)). In contrast, they all showed significantly enhanced fluorescence in glycerol, with Φ_F_ values exceeding 0.02 (Table 1, Figure 4). Group A derivatives exhibited Φ_F_ values in glycerol comparable to that of **DMAB‐Me** (Table 1), even though we had anticipated an increase in Φ_F_ upon cyclic amine substitution as was reported for rhodamine derivatives (e.g., Φ_F_ = 0.41 for dimethylamino rhodamine vs. Φ_F_ = 0.74 for its pyrrolidine analog) [36]. On the other hand, group B derivatives, in which the Ph—N bond rotation is constrained, exhibited Φ_F_ values ranging from 0.07 to 0.11 in glycerol—up to fourfold higher than that of **DMAB‐Me** (Φ_F_ = 0.025) (Table 1). As a side note, the fluorescence maxima of group B compounds were redshifted by 19–24 nm compared to **DMAB‐Me** (523 nm). Group C derivatives, featuring aromatic ring substitution on the amino group, also exhibited enhanced Φ_F_ values in glycerol. In particular, **DPhAB‐Me** showed a Φ_F_ of 0.078—approximately three times that of **DMAB‐Me** (Table 1). These derivatives also displayed notable bathochromic shifts, with the fluorescence maxima redshifted by 35–111 nm as compared to **DMAB‐Me**. Group D derivatives exhibited Φ_F_ values between 0.049 and 0.17 in glycerol, which represents up to a sevenfold increase relative to **DMAB‐Me** (Table 1). The fluorescence maxima were also significantly redshifted by 32–55 nm. Notably, **Julo‐Ph** showed the most redshifted absorption (515 nm) and emission (578 nm) wavelengths, as well as the highest fluorescence quantum yield (Φ_F_ = 0.17) among all the derivatives examined.

**TABLE 1:** Photophysical properties of DMAB derivatives synthesized in this study.

Group	GFP fluorophore	Methanol						Glycerol				Glycerol–methanol
		***λ*_abs_ (nm)**	***λ*_em_ (nm)**	Stokes’ shift (nm)	*I*_F_	Φ_F_		***λ*_abs_ (nm)**	***λ*_em_ (nm)**	Stokes’ shift (nm)	Φ_F_	*χ*
	**DMAB‐Me**	433	504	71	0.17	<0.01		452(450)^a^	523(518)^a^	71(68)^a^	0.025(0.027)^a^	0.69(0.68)^b^
A	**AzeB‐Me**	429	507	78	0.16	<0.01		445	524	79	0.023	0.63
	**PyrroB‐Me**	442	511	69	0.24	<0.01		461	527	66	0.03	0.63
	**PiperiB‐Me**	430	512	82	0.20	<0.01		449	529	80	0.026	0.54
	**MorphoB‐Me**	411	503	92	0.15	<0.01		425	521	96	0.024	0.62
B	**Julo‐Me**	465	531	66	0.46	<0.01		485	547	62	0.11	0.87
	**MeOJulo‐Me**	466	528	62	0.39	<0.01		484	542	58	0.07	0.82
C	**DPhAB‐Me**	433	603	170	0.65	<0.01		451	599	148	0.078	0.54
	**DMeOPhAB‐Me**	444	655	211	0.13	<0.01		460	634	174	0.026	0.59
	**CarbaB‐Me**	384	ND	ND	ND	<0.01		398	568	170	0.034	0.48
	**PhCarbaB‐Me**	402	486	84	0.20	<0.01		414	517	103	0.059	0.57
D	**DMAB‐Ph**	461(460)^c^	540(533)^c^	79(73)^c^	0.32	<0.01		480	555	75	0.049	0.78
	**PyrroB‐Ph**	469	547	78	0.39	<0.01		489	558	69	0.07	0.77
	**Julo‐Ph**	495	570	75	1.00	<0.01		515	578	63	0.17	0.72

a Reference [44], **1** (R_4_ = *tert*‐butyl) in glycerol.

b Reference [44], **1** (R_4_ = *tert*‐butyl) in glycerol–ethylene glycol.

c Reference [45], **DMAB‐Ph** (R = Me) in methanol. *λ*_abs_: Maximum absorption peak wavelength. *λ*_em_: Wavelength of maximum fluorescence excited at each *λ*_abs_. *I*_F_: Relative fluorescence intensity at the each *λ*_em_. Φ_F_: Fluorescence quantum yield measured using an integrating sphere.

The relationship between fluorescence quantum yield (Φ_F_) and solvent viscosity (*η*) for FMRs is described by the Förster–Hoffmann equation [46, 47, 48]

$$\log(\Phi_{F})=\chi \log(\eta )+C$$

**FIGURE 5:**
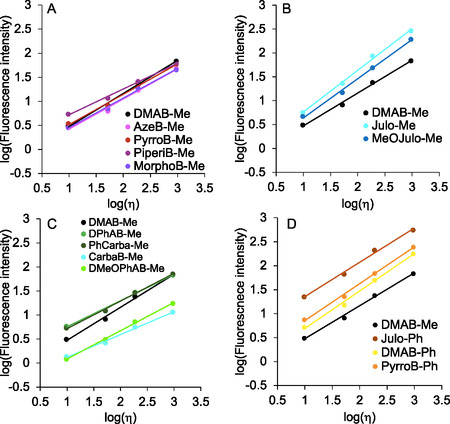
Viscosity dependency of fluorescence intensity of **DMAB** derivatives synthesized in this study. 15‐μM fluorophores were measured in glycerol–methanol mixed solvent. (A) cyclic amine modification, (B) julolidine modification, (C) aromatic amine modification, and D) phenyl modification at the imidazolinone ring.

**FIGURE 6:**
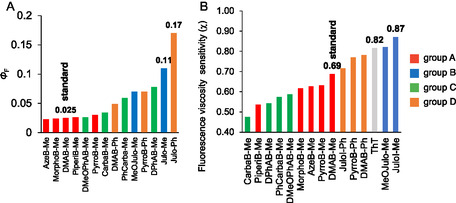
Summary of (A) Φ_F_ and (B) fluorescence viscosity sensitivity (*χ*) of **DMAB** derivatives used in this study.

Here, the *χ* value represents the sensitivity of fluorescence response to viscosity changes [47, 48, 49, 50]. We thus determined *χ* values for our **DMAB** derivatives from the fluorescence spectra in glycerol–methanol mixed solvent systems (Figure S2). The results are shown in Figures 5 and 6, and Table 1. For **DMAB‐Me**, the value we obtained (*χ* = 0.69) was in good agreement with the previously reported datum (*χ* = 0.68) [34]. The *χ* values for group‐A FMR derivatives (0.54–0.63) were lower than that of **DMAB‐Me** (Figure 5A). The relatively low *χ* values mainly arise from their low fluorescence intensities in high‐viscosity solvents. A similar trend was observed for group C derivatives, all of which also showed reduced *χ* values (Figure 5C). In contrast, group B derivatives exhibited higher *χ* values than **DMAB‐Me**, ranging from 0.82 to 0.87 (Figure 5B). The relatively high *χ* values for group B FMR derivatives are mainly accounted for by enhanced fluorescence intensity in high‐viscosity environments. Notably, **Julo‐Me** exhibited the highest *χ* value (0.87) among all the tested FMR derivatives, surpassing even the best record ever reported of thioflavin T (*χ* = 0.79) to the best of our knowledge [35]. Group D derivatives also showed a high viscosity sensitivity, with *χ* values from 0.72 to 0.78 (Figure 5D). Based on their high Φ_F_ values in glycerol and large *χ* values, we selected **Julo‐Me** and **Julo‐Ph** as promising FMR scaffolds for intracellular viscosity‐responsive imaging. **PyrroB‐Ph** was also included for comparison.

### 2.2 Excited State Calculation

**FIGURE 7:**
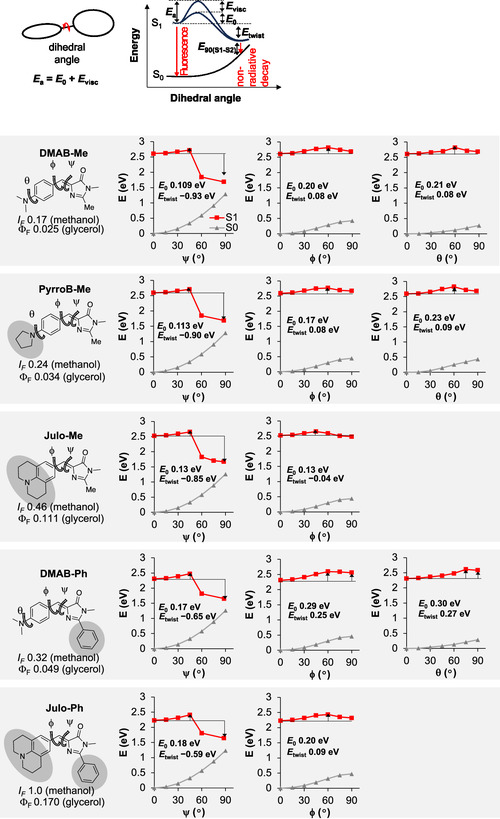
PEC of **DMAB** derivatives calculated with regard to the dihedral angles (*ψ*, *ϕ*, and *θ*). *E*_0_: energy barrier for twist, *E*_twist_: energy difference between planar and twisted state in the excited state. Calculations were performed at TD‐B3LYP/6‐31G+d level of theory. PEC = Potential energy curves.

The viscosity sensitivity of FMR fluorescence is governed by the ease of intramolecular bond twisting. In particular, in the excited state (S_1_), the rotational energy barrier (*E*_a_) around the dihedral axis of interest can be interpreted as the sum of the intrinsic barrier in vacuum (*E*_0_) and the solvent‐derived barrier (*E*_visco_) that is most significantly influenced by the molecular surface area of the FMR (Figure 7) [51]. To understand how the molecular structure of our **DMAB** derivatives impacts on rotational flexibility, we estimated the potential energy curves (PEC) of the ground state (S_0_) and excited state (S_1_) with regard to three key dihedral angles (*ψ*, *ϕ*, and *θ*) by time‐dependent density functional theory (TD‐DFT) [52] calculations (Figure 7, S3).

Prior studies on **DMAB** derivatives have shown that 0°‐to‐90° twisting around the double bond (*ψ*) in S_1_ leads to a drop in energy, which approaches the S_0_ PEC and facilitates nonradiative decay via the TICT mechanism [45, 53, 54]. For **DMAB‐Ph**, an energy difference of *E*_90_ (S_1_–S_0_) ≈0.4 eV at *ψ* = 90° has been reported [45]. Consistently, our calculations showed almost the same *E*_90_ (S_1_–S_0_) values for **DMAB‐Me** (0.40 eV), **DMAB‐Ph** (0.39 eV), **PyrroB‐Me** (0.41 eV), **Julo‐Me** (0.41 eV), and **Julo‐Ph** (0.41 eV). These energy differences at a dihedral angle of 90° are much smaller than those calculated for *ϕ* and *θ* (>2.0 eV), supporting the critical role of *ψ* twisting and irrelevance of the other twistings (*ϕ* and *θ*) in fluorescence quenching.

**FIGURE 8:**
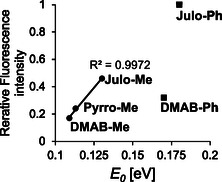
Correlation of *E*_0_ with the relative fluorescence intensity in methanol for **DMAB‐Me**, **Pyrro‐Me**, **Julo‐Me**, **DMAB‐Ph**, and **Julo‐Ph** (TD‐B3LYP/6‐31G+d level of theory).

Assuming that the C—N bond twisting (*θ*) is unrelated to the fluorescence quenching, we next focus on the substituent effects of dimethylamino, pyrrolidine, and julolidine groups in the methyl‐substituted **DMAB** derivatives (**DMAB‐Me**, **PyrroB‐Me**, **Julo‐Me**) on the *ψ* twisting barrier *E*_0_ of the double bond in the excited state under vacuum. *E*_0_ correlates well with their fluorescence intensity (*I*_F_) in methanol (Figure 8), suggesting that a larger group causes a higher barrier on the double‐bond rotation (*ψ*), thus giving a brighter fluorescence. Similar substituent effects are found between two phenyl‐substituted derivatives, **DMAB‐Ph** and **Julo‐Ph**, and between two 2‐substituted‐imidazolinone derivatives, **DMAB‐Ph** and **DMAB‐Me** (Figure 8). These analyses demonstrate that larger substituents on both sides of the center double bond of **DMAB** derivatives tend to slow its rotation (*ψ*) in the excited states even without viscous solvents, emitting a brighter fluorescence. Since larger substituents generally have larger surface areas, which promote more frequent collisions with solvent molecules, the substituent effects should be more pronounced in highly viscous solvents.

**FIGURE 9:**
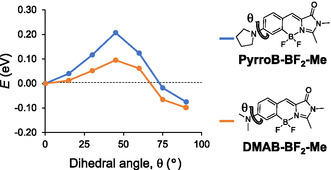
PEC of **PyrroB‐BF_2_‐Me** and **DMAB‐BF_2_‐Me** calculated as a function of the dihedral angle (*θ*) of the Ph—N bond in the S_1_ state at the TD‐B3LYP/6‐31+G(d) level of theory.

Unlike our results, there is a report that the C–N twisting (*θ*) in DMAB derivatives appears to participate in fluorescence quenching. **Pyrro‐BF_2_‐Me** displayed a significantly higher fluorescence (Φ_F_ = 0.33) than **DMAB‐BF_2_‐Me** (Φ_F_ = 0.03) [40], though the bond rotations (*ψ* and *ϕ*) directly associated with the double bond were restricted (Figure 9). Our TD‐DFT calculations reproduced the potential curves supportive of the C–N twist‐based fluorescence quenching of these compounds. The twisted excited states at *θ* = 90° are more stable than the planar structures (*θ* = 0°), which is the most prominent difference from the *θ*‐dependent potential curves for our **DMAB** derivatives. The barrier difference (Δ*E*_0_) between **DMAB‐BF_2_‐Me** (0.10 eV) and **Pyrro‐BF_2_‐Me** (0.21 eV) explains the enhanced fluorescence of **Pyrro‐BF_2_‐Me**.

Overall, although the rotations of the *σ* bonds other than the central double bond in **DMAB** derivatives may contribute to fluorescence quenching, it should happen only when the double‐bond rotation is restricted. Our results indicate that in **DMAB**‐type fluorophores, where no rotational constraints are imposed on the central double bond, the *ψ* twisting at the excited state is the dominant pathway for nonradiative deactivation. It is worthy of attention that there is a report that nonradiative transitions in protic solvents, such as alcohols, are most likely due to hydrogen bonding interactions between the solvent and the fluorescent solute, and TICT might be barely involved [55]. The calculations in our study were performed under a polarizable continuum model as a solvent approximation and might have not well reproduced the potential energy of each state in alcohol. Further studies are necessary to resolve this issue. The enhanced Φ_F_ values for julolidine‐containing derivatives (**Julo‐Ph** and **Julo‐Me**) as compared with the other **DMAB** derivatives in this study are probably due to its large size and not originate from the fixation of C–N rotation.

**FIGURE 10:**
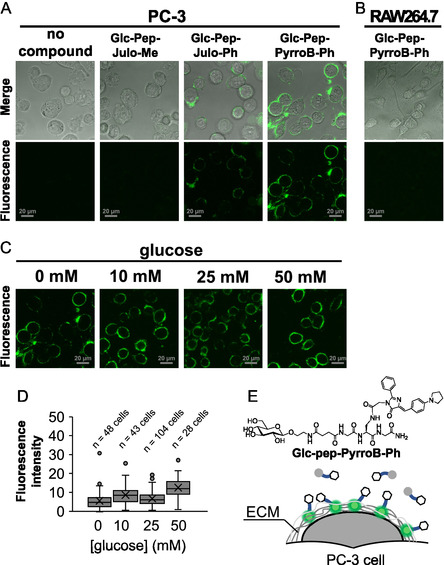
Wash‐free fluorescence CLMS images of (A) PC‐3 cells with peptide modified 10‐µM **Glc‐Pep‐Julo‐Me**, **Glc‐Pep‐PyrroB‐Ph**, and **Glc‐Pep‐Julo‐Ph** and (B) CLMS images of RAW264.7 cells with 10‐µM **Glc‐Pep‐PyrroB‐Ph**. The grayscale bars correspond to 20 µm. (C) Wash‐free fluorescence CLMS images of PC‐3 cells with 10‐µM **Glc‐Pep‐PyrroB‐Ph** in the absence and presence of glucose (10, 25, 50 mM), and (D) the quantified fluorescence intensity at each glucose concentration. Fluorescence intensities were quantified for individual cells, with a total of *n* cells analyzed from three fields of view for each condition. Error bars represent standard deviation. (E) Schematic illustration of fluorescence imaging of PC‐3 with **Glc‐Pep‐PyrroB‐Ph.** CLMS = Confocal laser scanning microscope.

**FIGURE 11:**
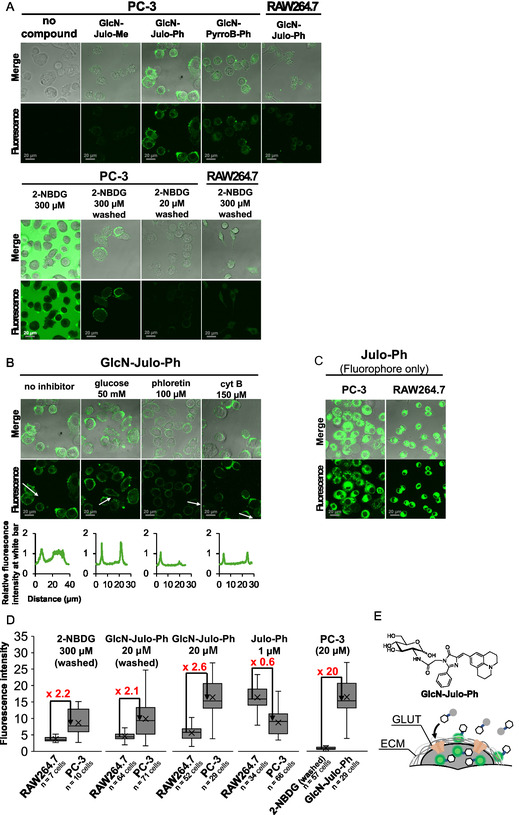
Wash‐free fluorescence CLMS images of (A) PC‐3 cells with 20‐µM **GlcN‐Julo‐Me**, **GlcN‐Julo‐Ph, GlcN‐PyrroB‐Ph**, and 300‐µM **2‐NBDG**. For the **2‐NBDG** imaging, “washed” images were obtained after washing. (B) Inhibition of **GlcN‐Julo‐Ph** uptake into PC‐3 cells with glucose (50 mM), phloretin (100 μM), and cytochalasin B (150 μM). (C) Wash‐free images of PC‐3 and RAW264.7 cells with 5‐µM **Julo‐Ph** (fluorophore only). The grayscale bars correspond to 20 µm. (D) Quantification of fluorescence intensity of each cell image (for detail, see supporting information (SI)). Fluorescence intensities were quantified for individual cells, with a total of *n* cells analyzed from three fields of view for each condition. Error bars represent standard deviation. (E) Schematic illustration of fluorescence imaging with **GlcN‐Julo‐Ph**. CLMS = Confocal laser scanning microscope.

On developing turn‐on fluorescent probes that emit only upon binding to GLUT or GLUT‐mediated internalization, we adopted **Julo‐Me**, **Julo‐Ph**, and **PyrroB‐Ph** scaffolds, which are highly viscosity sensitive with sufficient fluorescence strengths. As mentioned above, **2‐NBDG**, *N*‐NBD‐glucosamine for fluorescence imaging of cancer cells, is known to be taken up into cancer cells via GLUTs [12, 13], which prompts us to adopt *N*‐substituted glucosamine derivatives for passage through the GLUT channels. We thus synthesized **GlcN‐Julo‐Me**, **GlcN‐PyrroB‐Ph**, and **GlcN‐Julo‐Ph** by conjugating GFP fluorophores to the amino group of glucosamine via amide bond (Figure 2C, Scheme S2). In parallel, we prepared **Glc‐Pep‐Julo‐Me**, **Glc‐Pep‐Julo‐Ph,** and **Glc‐Pep‐PyrroB‐Ph** by conjugating glucose and GFP fluorophore derivatives through a peptide linker (Figure 2C, Scheme S3). These compounds were evaluated for wash‐free fluorescence imaging of cancer cells by using PC‐3 cells (GLUT‐high) and RAW264.7 murine macrophage cells as a nontumor control (Figure 10, 11, S4–S6).

### 2.3 Cellular Imaging

First, we tested cell imaging with the peptide‐conjugated glucose probes under wash‐free conditions with a confocal laser scanning microscope (CLSM). **Glc‐Pep‐Julo‐Ph** and **Glc‐Pep‐PyrroB‐Ph** showed a relatively strong fluorescence emission localized near the cell membrane of PC‐3 cells (Figure 10A), whereas no significant fluorescence emission was detected for RAW264.7 cells (Figure 10A). To determine if this fluorescence arose from a specific binding of the probes to GLUT, we performed a glucose competition assay of **Glc‐Pep‐PyrroB‐Ph** (Figure 10C) [15]. The fluorescence intensity remained unchanged upon glucose addition as was quantitated in Figure 10D, in which the fluorescence intensity derived from cells was quantified by ImageJ [56]. The immunostaining of the **Glc‐Pep‐PyrroB‐Ph**‐treated PC‐3 with an anti‐GLUT1 antibody revealed no significant colocalization of **Glc‐Pep‐PyrroB‐Ph** and the antibodies (Figure S4). These results suggest that the observed fluorescence was not due to a specific binding of **Glc‐Pep‐PyrroB‐Ph** with GLUT. Notably, the fluorescent contours for PC‐3 cells appeared as fibrous structures extending out from the outer membrane, suggesting that **Glc‐Pep‐PyrroB‐Ph** might have bound nonspecifically to hydrophobic structures such as the extracellular matrix (ECM) (Figure 10E). It is known that collagen, which is one of the major components of the ECM, is excessively abundant in cancer cells [57]. Previously, a BODIPY‐based FMR that exhibits fluorescence upon binding to collagen‐like peptides has been reported [58]. **Glc‐Pep‐Julo‐Ph** and **Glc‐Pep‐PyrroB‐Ph** might have exhibited similar behavior. The complete absence of fluorescence in the noncancerous RAW264.7 cells supports this interpretation (Figure 10B).

Next, we examined glucosamine‐conjugated probes (Figure 11). Whereas **GlcN‐Julo‐Me** gave no clear cell staining, **GlcN‐Julo‐Ph** and **GlcN‐PyrroB‐Ph** yielded clear fluorescence emissions at the cell membrane and partially in cytoplasmic region of PC‐3 cells under wash‐free conditions, whereas only a weak fluorescence emission was observed for RAW264.7 cells (Figure 11A). The wash‐free image of PC‐3 treated with **2‐NBDG** demonstrates an apparent difference from that treated with **GlcN‐Julo‐Ph** or **GlcN‐PyrroB‐Ph**. The medium is brightened and the cells look dark. Only after washing, did the clear fluorescence emissions from PC‐3 cells appear, but the concentration of **2‐NBDG** (300 µM) required for this staining was 15 times higher than that of **GlcN‐Julo‐Ph** (20 µM).

To assess whether the observed fluorescence of the key probe **GlcN‐Julo‐Ph** arose from GLUT‐mediated uptake, we conducted inhibition studies using glucose, phloretin, and cytochalasin B (Figure 11B) [15]. In the PC‐3 cells treated with the inhibitors, the membrane‐associated, fibrously delineated fluorescence emissions of **GlcN‐Julo‐Ph** persisted. On the other hand, the intracellular fluorescence was significantly reduced by the addition of the inhibitors, as obviously demonstrated by the sectional fluorescence intensities of single cells (Figure 11B bottom) and also quantified fluorescence intensity (Figure S5), which suggests a GLUT‐dependent internalization of **GlcN‐Julo‐Ph** (Figure 11E).

The membrane‐associated fluorescence emissions of **GlcN‐Julo‐Ph** disappeared after washing (Figure S6), which suggests an ECM binding as discussed above for **Glc‐Pep‐PyrroB‐Ph**. **Julo‐Ph**, which lacks glucosamine, was up‐taken moderately by PC‐3 and substantially by RAW264.7, as shown in Figure 11C. These results imply that the glucosamine moiety might have acted as a protector from uptakes by penetration through the cell membrane and as such **GlcN‐Julo‐Ph** could have been taken up only by GLUT‐overexpressed cells.

The fluorescence activation of intracellular **GlcN‐Julo‐Ph** is likely due to its response to intracellular viscosity, which is reported to range from 100 to 400 cP [30, 59, 60, 61]. Under the equivalent conditions in glycerol–methanol mixtures, Φ_F_(viscous) of **GlcN‐Julo‐Ph** was calculated to range from 0.03 to 0.09 (Figure S7), more than 3–9 times higher than the Φ_F_(methanol) value in methanol only (Φ_F_ < 0.01), which indicates the observed intracellular fluorescence as a result of viscosity response. On the other hand, **GlcN‐Julo‐Me** showed a lower response to viscosity (a Φ_F_(viscous)/Φ_F_(methanol) value of 2–5), which may explain the lack of strong intracellular fluorescence (Figure S7). Another possible explanation for the intracellular fluorescence activation is that the relatively high lipophilicity of **GlcN‐Julo‐Ph** could facilitate hydrophobic interactions with intracellular proteins, restricting intramolecular motion and enhancing fluorescence.

The CLMS images clearly demonstrate that **GlcN‐Julo‐Ph** is superior to **2‐NBDG** in enabling a selective PC‐3 cancer cell imaging without washing steps with less amount. We then estimated how much selective are these probes and methods toward PC‐3 over RAW264.7. The ratios of the median fluorescence intensities of PC‐3 cells over those of RAW264.7 cells (PC‐3/RAW) were calculated for the cell images with each fluorophore with (w/W) or without (w/oW) washings. The ratios of PC‐3/RAW values of **2‐NBDG** (w/W), **GlcN‐Julo‐Ph** (w/W), **GlcN‐Julo‐Ph** (w/oW), and **Julo‐Ph** (w/oW) are 2.2, 2.1, 2.6, and 0.6, respectively (Figure 11D). When compared at the same concentration (20 µM), **GlcN‐Julo‐Ph** exhibited 20‐fold stronger fluorescence in PC‐3 cells than **2‐NBDG** (Figure 11D). Interestingly, **GlcN‐Julo‐Ph** gave a better selectivity w/oW than w/W conditions, which may indicate that a part of the probe molecules were specifically bound to the surface GLUTs and the washing step removed them in addition to the nonspecifically bound probes on the cell surface.

**FIGURE 12:**
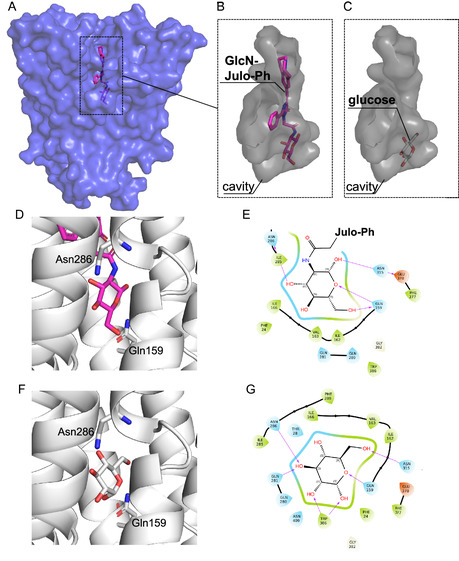
Docking simulation of **GlcN‐Julo‐Ph** toward GLUT3 (PDBID:4zw9) by using Glide docking program from Schrodinger Maestro. (A) GLUT3 with **GlcN‐Julo‐Ph** docked, (B) expanded structure of docked **GlcN‐Julo‐Ph** with cavity (Glide score: −7.616 kcal/mol), (C) expanded structure of glucose from X‐ray cocrystal GLUT3 structure with cavity. (D) Expanded view of the sugar moiety of docked **GlcN‐Julo‐Ph** in the substrate‐binding site of GLUT3. (E) Ligand interaction diagram of docked **GlcN‐Julo‐Ph** in GLUT3. (F) Expanded view of glucose in the substrate‐binding site of the GLUT3 cocrystal structure. (G) Ligand interaction diagram of glucose in the GLUT3 cocrystal structure. For (E) and (G), the violet lines indicate hydrogen‐bonding interactions.

The above results indicate that GLUTs are necessary for the uptake of **GlcN‐Julo‐Ph** into PC‐3, but how GLUTs worked to internalize the fluorophore, i.e., whether by endocytosis or channel passage, is unknown. Tanasova and coworkers have reported, based on docking studies, that rhodamine glycoconjugates can be accommodated by GLUT‐family transporters despite their relatively large size, suggesting that they could plausibly pass through the transporter during the conformational changes associated with the transition from the outward‐open to inward‐open state [17, 18]. Their work included docking studies of the mannitol‐based rhodamine probe **ManRho** with GLUT5 [18], and, more recently, of the 6‐amino‐glucose‐derived rhodamine probe **GluRho** with inward‐open GLUT1 and outward‐open GLUT2 [17]. We also ran docking simulations to test whether our probe could be recognized by GLUT1. Since no outward‐open GLUT1 structure is available, we instead docked **GlcN‐Julo‐Ph** into the glucose‐bound, outward‐occluded cocrystal structure of the highly homologous GLUT3 [62] (Figure 12A). The results suggested that the sugar moiety of **GlcN‐Julo‐Ph** partially overlaps with the canonical glucose‐recognition region of GLUT3 [62]. In particular, hydrogen bonds were predicted between the 3‐hydroxyl group and Asn286, and between the ring oxygen/5‐hydroxyl group and Gln159 (Figure 12B,C). Notably, these residues are also involved in glucose recognition in the original GLUT3 cocrystal structure (Figure 12F,G), supporting a glucose‐like binding mode of the probe to some extent, while the **Julo‐Ph** moiety is accommodated within the extracellular‐gate region. Although docking alone does not constitute definitive proof of translocation, these structural insights, together with the imaging and inhibition data, support the possibility that **GlcN‐Julo‐Ph** adopts a transport‐compatible binding mode in a GLUT‐family transporter.

**FIGURE 13:**
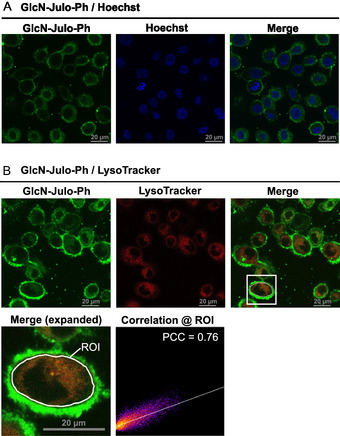
Colocalization analysis of **GlcN‐Julo‐Ph‐**treated PC‐3 cells using (A) Hoechst and (B) LysoTracker Deep Red. The grayscale bars correspond to 20 µm.

To examine whether **GlcN‐Julo‐Ph** bound to GLUTs contributes to the fluorescence observed near the cell periphery, we performed costaining with an anti‐GLUT1 antibody and the **GlcN‐Julo‐Ph** probe (Figure S8). Although regions positive for GLUT1 were often accompanied by **GlcN‐Julo‐Ph** fluorescence, the probe‐derived fluorescence was also observed in peripheral regions where no apparent red fluorescence from GLUT1 was detected. Thus, the overall staining pattern did not support a clear one‐to‐one correlation between GLUT1 and **GlcN‐Julo‐Ph** fluorescence, suggesting that the fluorescence of **GlcN‐Julo‐Ph** observed around the cell contour may arise, at least in part, from binding to ECM. To further explore subcellular localization, we performed costaining with Hoechst and LysoTracker Deep Red. The intracellular regions lacking signal during **GlcN‐Julo‐Ph** staining were identified as nuclei based on costaining with Hoechst (Figure 13A). A high degree (Pearson's correlation coefficient = 0.76) of localization overlaps between LysoTracker and **GlcN‐Julo‐Ph** was observed, indicating that **GlcN‐Julo‐Ph** mainly exhibits fluorescence in lysosomes (Figure 13B).

Lastly, we evaluated the cytotoxicity of **GlcN‐Julo‐Ph** and **2‐NBDG** (20 µM each) toward PC‐3 and RAW264.7 cells after 24 h of incubation. **GlcN‐Julo‐Ph** exhibited slight cytotoxicity in both PC‐3 and RAW264.7 cells (23% and 16% inhibition, respectively), which was comparable to that induced by **2‐NBDG** (25% and 14% inhibition, respectively, Figure S9). Considering that **2‐NBDG** has been successfully employed for in vivo imaging in previous studies [15, 63], **GlcN‐Julo‐Ph** can also be expected to be suitable for imaging applications.

## 3 Conclusion

In this study, we developed new FMRs, i.e., glucose‐conjugated **DMAB** probes designed for wash‐free, turn‐on imaging of cancer cells. Our approach focused on optimizing both the fluorescence quantum yield (Φ_F_) and viscosity sensitivity (*χ* value) to match the high‐viscosity intracellular environment. We synthesized a series of **DMAB** derivatives with modified amino groups or extended aromatic frameworks. Guided by TD‐DFT calculations, we identified structural features that enhance excited‐state rigidity and fluorescence. Among them, **Julo‐Me** exhibited the highest *χ* value (0.87), while **Julo‐Ph** demonstrated both an excellent *χ* value (0.72) and a satisfactory Φ_F_ in viscous media (0.17 in glycerol), making them promising scaffolds for intracellular turn‐on probes. To achieve GLUT‐targeted uptake, we conjugated these fluorophores to glucosamine or glucose via amide or peptide linkages. Wash‐free fluorescence imaging demonstrated that **GlcN‐Julo‐Ph**, in particular, produced a strong intracellular fluorescence with GLUT‐overexpressing PC‐3 cancer cells. Inhibition experiments confirmed that uptake was GLUT‐mediated. Unlike conventional fluorescent glucose tracers such as **2‐NBDG**, our probes enabled rapid, low‐concentration, and wash‐free imaging. A comparison between **GlcN‐Julo‐Me** and **GlcN‐Julo‐Ph** revealed that both high Φ_F_ and *χ* values are critical for effective intracellular visualization under wash‐free conditions. Although further in vivo validation will be necessary, the present results provide a promising cell‐level proof of concept for the development of glucose‐conjugated FMR probes aimed at wash‐free imaging of cancer cells.

## Supporting Information

Additional SI can be found online in the Supporting Information section.

## Funding

This work was supported by Japan Society for the Promotion of Science (JP16K16640, JP18K05352, JP22K05347, JP25K08853, and JP25K08821), Toyota Physical and Chemical Research Institute, Koyanagi Foundation, and Research Foundation for Opto‐Science and Technology.

## Conflicts of Interest

The authors declare no conflicts of interest.

## Supporting information

Supplementary Material

## Data Availability

The data that support the findings of this study are available in the SI of this article.
