# Supplementary material for: Glucose‐Modified Viscosity‐Responsive Turn‐On Fluorescence Molecular Rotors for Wash‐Free Live‐Cell Imaging
Source: Chembiochem. 2026 May 17;27(10):e70372. doi: 10.1002/cbic.70372 (PMC13181232; doi:10.1002/cbic.70372)
Supplement: Supplementary file 1 — Supplementary Material [file CBIC-27-e70372-s001.pdf]

Supplementary Information

Glucose-Modified Viscosity-Responsive Turn-On Fluorescence Molecular Rotors for Wash-Free Live-Cell Imaging

Takashi Kanamori,*[a] Yuki Sadai,[a] Kenji Hida,[a] Takayuki Tsuduki,[a] Chihiro Nogi,[a] Hiroya Asami,[b] Shun-ichiro Ogura,[a] and Hideya Yuasa[a]

[a] School of Life Science and Technology, Institute of Science Tokyo, J2-10 4259 Nagatsuta, Midoriku, Yokohama 226-8501, Japan

[b] Department of Chemistry, Faculty of Science, Gakushuin University, 1-5-1 Mejiro, Toshima-ku, Tokyo 171-8588, Japan

[b] Present Address: Computational Science Group, Analysis Research Department, Chemical Research Laboratories, Nissan Chemical Corporation, 2-10-1 Tsuboi-Nishi, Funabashi-shi, Chiba 274-8507, Japan

Table of contents

1. **Experimental section**
   - **General methods -- S3**
   - **Synthetic protocols of compounds --- S4~S11**
   - **Photophysical measurements -- S12**
   - **Quantum chemical calculations -- S12**
   - **Molecular docking simulations -- S12**
   - **Synthetic protocols of GFP fluorophore-modified probes ------------------------------------ S12~S13**
   - **Cellular experiment protocols -- S13~S15**
2. **Supplemental Schemes and Figures**
   - **Synthetic Scheme of fluorophore monomers -- S16**
   - **UV and fluorescence spectra-- S17~18**
   - **Optimized structures obtained by DFT and TD-DFT calculations -------------------------- S19~38**
   - **Synthetic Scheme of Glucosamine probes --- S39**
   - **Synthetic Scheme of Glucose-peptide probes --- S39**
   - **Confocal imaging -- S40~41**
   - **Relationship between viscosity and fluorescence quantum yield ---------------------------- S41**
   - **Cytotoxicity -- S43**
   - **^{1}H NMR, ^{13}C NMR, and ESI-TOF-MS spectra of compounds ------------------------------ S44~80**
   - **HPLC charts and ESI-TOF-MS spectra of probes -- S81~86**
3. **References -- S87**

General methods

All reagents and starting materials were purchased from Wako Pure Chemical Industries, Ltd.; Tokyo Chemical Industry Co., Ltd.; Kanto Chemical Co., Inc.; Sigma Aldrich Co., Nacalai Tesque, Inc. Water used for the organic synthesis was purified by Milli-Di, Merck Millipore. For the spectroscopic measurement, spectroscopic analysis grade of solvents from Wako Pure Chemical Industries, Ltd. or Kanto Chemical Co., Inc. were used. Thin-layer chromatography (TLC) was performed on pre-coated silica gel Merck 60-F_{254} plates and visualized by the UV light (AS ONE SLUV-6, 254 or 365 nm) or charring after immersing in a solution of 1% $Ce(SO_4)_2$·1.5% $(NH_4)_6Mo_7O_{24}\cdot 4H_2O$ in 10% H_2SO_4. Column chromatography was performed on Kanto silica gel 60N (spherical, neutral, 40-50 μm) or Biotage Sfär HC D, or Fuji Silysia NH silica gel (NH-DM1020) with the solvent system specified. ^{1}H NMR spectra were recorded at 400 MHz (JEOL JNM-ECZL400S) or 500 MHz (Varian Unity INOVA 500 or Bruker AVANCEIII HD 500). The tetramethylsilane peak (δ = 0.00 ppm) or a solvent peak was used as a standard in $CDCl_3$ (δ = 7.26 ppm), CD_3OD (δ = 3.31 ppm) or $(CD_3)_2SO$ (δ = 2.50 ppm). Chemical shifts are expressed in ppm referenced to the standards. The multiplicity of the signals is abbreviated as follows: s = singlet, d = doublet, dd = doublet of doublets, t = triplet, quin = quintet, br = broad signal, and m = multiplet. ^{13}C NMR spectra were recorded at 126 MHz (Varian Unity INOVA 500 or Bruker AVANCEIII HD 500) and a solvent peak of $CDCl_3$ (δ = 77.16 ppm), CD_3OD (δ = 49.00 ppm) or $(CD_3)_2SO$ (δ = 39.52 ppm) was used as a standard. High-resolution mass spectra (HRMS) were recorded on a Bruker micrOTOF II ESI-TOF MS.

Synthetic protocols of compounds

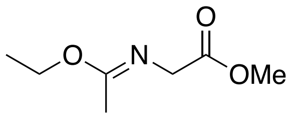

Methyl 2-(1-ethoxyethylidene)aminoethanoate: This compound was synthesized following the previous report.[1] To a solution of glycine methyl ester hydrochloride (12.6 g, 100 mmol) and K_2CO_3 (15.5 g, 112 mmol) in Et_2O (200 mL) in extraction funnel, were added ethyl acetimidate hydrochloride (12.4 g, 100 mmol) then water (40 mL). The reaction mixture was shaken vigorously then 20 mL-water was added and shaken again. Then the organic layer was collected and dried over Na_2SO_4 and evaporated to give colorless liquid **1** (8.0 g, 50%); ^{1}H NMR spectrum was identical to the previous report[1]; ^{1}H NMR (500 MHz, $CDCl_3$, 298 K) δ (ppm) 4.03 (q, 2H, J = 7.1 Hz, $-OC\underline{H_2}CH_3$), 3.97 (s, 2H, $-COCH_2-$), 3.66 (s, 3H, $-OCH_3$), 1.80 (s, 3H, $-CH_3$), 1.18 (t, 3H, J = 7.1 Hz, $-OCH_2C\underline{H_3}$).

General procedure for the preparation of amino modified GFP dye derivatives.

The following procedure was adapted from the literature.[2] Methyl 2-(1-ethoxyethylidene)aminoethanoate (2.2 eq) and corresponding benzaldehyde (1.0 eq) were dissolved in dry toluene (2.1 ~ 5.0 mL). With vigorous stirring, the sealed mixture was heated at 75 ºC for 12-19 h. Then the reaction mixture was heated at 100 °C for 0-19 h if needed based on the TLC result. Then it was diluted with dichloromethane and evaporated. The remaining residue was purified by silica gel column chromatography (hexane/ethyl acetate).

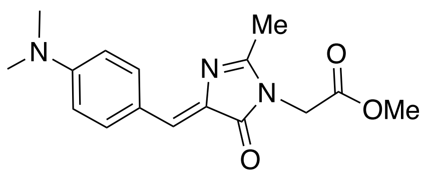

DMABI-Me: 4-(dimethylamino)benzaldehyde (427 mg, 2.86 mmol) and imidate **1** (1.00 g, 6.28 mmol) in toluene (5 mL) were stirred at 75 °C for 36 h. Then the mixture was purified by column chromatography (N60, hexane/ethyl acetate, 1:1) to give **DMABI-Me** (196 mg, 23%). R_f 0.25 (hexane/ethyl acetate, 1:1); ^{1}H NMR spectrum was identical to the literature.[2] ^{1}H NMR (500MHz, $CDCl_3$, 298 K) δ (ppm) 8.07 (d, 2H, J = 8.8 Hz, Ar-H), 7.12 (s, 1H, Vinyl-H), 6.70 (d, 2H, J = 9.0 Hz, Ar-H), 4.40 (s, 2H, $-COCH_2-$), 3.77 (s, 3H, $-OCH_3$), 3.06 (s, 6H, $-NCH_3$ x2), 2.32 (s, 3H, $-CCH_3$).

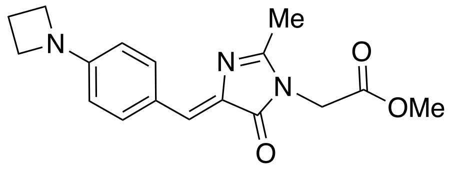

AzeB-Me: 4-(Azetidin-1-yl)benzaldehyde[3] (500 mg, 3.10 mmol) and imidate **1** (1.28 g, 8.04 mmol) in toluene (6 mL) were stirred at 75 °C for 3 days. Then the mixture was purified by column chromatography (N60, hexane/ethyl acetate, 4:1) to give **AzeB-Me** (737 mg, 76%). R_f 0.20 (hexane/ethyl acetate, 1:1); ^{1}H NMR (500 MHz, $CDCl_3$, 298 K) δ (ppm) 8.03 (d, 2H, J = 8.7 Hz, Ar-H), 7.10 (s, 1H, Vinyl-H), 6.39 (d, 2H, J = 8.8 Hz, Ar-H), 4.39 (s, 2H, $-COCH_2-$), 3.99 (t, 4H, J = 7.3 Hz, $-N(CH_2)_2-$), 3.77 (s, 3H, $-OCH_3$), 2.41 (pentet, 2H, J = 7.3 Hz, $-NCH_2C\underline{H_2}-$), 2.31 (s, 3H, $-CCH_3$); ^{13}C NMR (126 MHz, $CDCl_3$, 298K)δ 170.15, 168.43, 157.88, 152.81, 134.36, 134.16, 130.03, 122.93, 110.72, 52.83, 51.76, 41.43, 16.66, 15.55; ESI-TOF-MS m/z calcd for $C_{17}H_{19}N_3O_3Na^+$: 336.1319, $[M+Na]^+$; found: 336.1319.

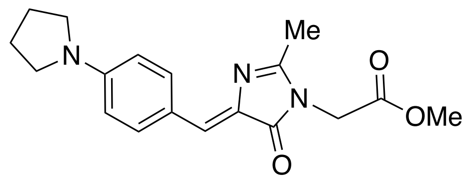

PyrroB-Me: 4-(Pyrrolidin-1-yl)benzaldehyde (501 mg, 2.86 mmol) and imidate **1** (1.00 g, 6.28 mmol) in toluene (5 mL) were stirred at 75 °C overnight. Then the reaction mixture was further stirred at 100 °C for 19 h. Then the mixture was purified by column chromatography (N60, hexane/ethyl acetate, 5:2) to give **PyrroB-Me** (364 mg, 39%). R_f 0.25 (hexane/ethyl acetate, 1:1); ^{1}H NMR (500 MHz, $CDCl_3$, 298 K) δ (ppm) 8.06 (d, 2H, J = 8.9 Hz, Ar-H), 7.12 (s, 1H, Vinyl-H), 6.57 (d, 2H, J = 9.1 Hz, Ar-H), 4.40 (s, 2H, -$COCH_2$-), 3.77 (s, 3H, -OCH_3), 3.37 (m, 4H, -$N(CH_2)_2$-), 2.31 (s, 3H, -CCH_3), 2.03 (m, 4H, -$NCH_2\underline{CH_2}$-); ^{13}C NMR (126 MHz, $CDCl_3$, 298 K) δ (ppm) 170.01, 168.39, 157.21, 149.31, 134.62, 133.40, 130.16, 121.58, 111.82, 52.70, 47.56, 41.31, 25.45, 15.41; ESI-TOF-MS m/z calcd for $C_{18}H_{21}N_3O_3Na^+$: 350.1475, $[M+Na]^+$; found: 350.1472.

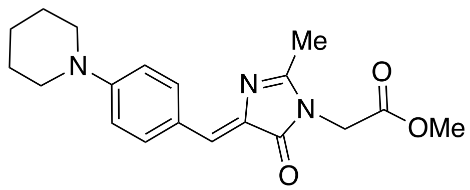

PiperiB-Me: 4-(piperidin-1-yl)benzaldehyde[3] (500 mg, 2.64 mmol) and imidate **1** (1.05 g, 6.60 mmol) in toluene (6 mL) were stirred at 75 °C for 3 days. Then the mixture was purified by column chromatography (N60, hexane/ethyl acetate, 4:1) to give **PiperiB-Me** (631 mg, 70%). R_f 0.23 (hexane/ethyl acetate, 1:1); ^{1}H NMR (500 MHz, $CDCl_3$, 298 K) δ (ppm) 8.04 (d, 2H, J = 8.9 Hz, Ar-H), 7.10 (s, 1H, Vinyl-H), 6.88 (d, 2H, J = 9.0 Hz, Ar-H), 4.39 (s, 2H, -$COCH_2$-), 3.77 (s, 3H, -OCH_3), 3.34 (t, 4H, J = 5.1 Hz, -$N(CH_2)_2$-), 2.31 (s, 3H, -CCH_3) 1.71-1.63 (m, 6H, -$NCH_2\underline{CH_2CH_2CH_2}CH_2N$-); ^{13}C NMR (126MHz, $CDCl_3$, 298K) δ (ppm) 170.19, 168.41, 158.25, 152.77, 134.64, 134.38, 129.53, 123.63, 114.49, 52.85, 48.88, 41.43, 25.53, 24.52, 15.55; ESI-TOF-MS m/z calcd for $C_{19}H_{23}N_3O_3Na^+$: 364.1632, $[M+Na]^+$; found: 364.1636.

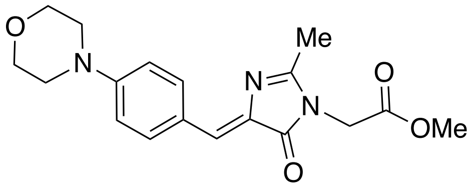

MorphoB-Me: 4-(Morpholin-4-yl)benzaldehyde (547 mg, 2.86 mmol) and imidate **1** (1.00 g, 6.28 mmol) in toluene (5 mL) were stirred at 75 °C for 17 h. Then the reaction mixture was further stirred at 100 °C for 19 h. Then the mixture was purified by column chromatography (N60, hexane /ethyl acetate, 9:11) to give **MorphoB-Me** (686 mg, 70%). R_f 0.14 (hexane/ethyl acetate, 1:1); ^{1}H NMR (500 MHz, $CDCl_3$, 298 K) δ 8.08 (d, 2H, J = 8.9 Hz, Ar-H), 7.10 (s, 1H, Vinyl-H), 6.90 (d, 2H, J = 9.2 Hz, Ar-H) 4.40 (s, 2H, -$COCH_2$-), 3.86 (t, 4H, J = 4.9 Hz, -CH_2- x2), 3.78 (s, 3H, -OCH_3), 3.29 (t, 4H, J = 4.9 Hz, -CH_2- x2) 2.32 (s, 3H, -CCH_3); 13C NMR (126 MHz, $CDCl_3$, 298K) δ 170.13, 168.30, 159.05, 152.37, 135.48, 134.15, 128.84, 125.15, 114.40, 66.73, 52.86, 47.83, 41.39, 15.57; ESI-TOF-MS: m/z calcd for $C_{18}H_{21}N_3O_4Na^+$: 366.1424 $[M+Na]^+$; found: 366.1419.

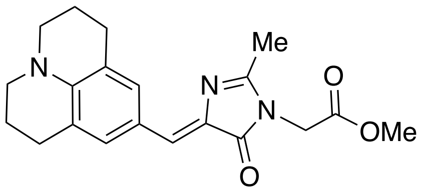

Julo-Me: 9-Julolidinecarboxaldehyde (577 mg, 2.87 mmol) and imidate (1.00 g, 6.30 mmol) in toluene (5 mL) were stirred at 75 °C for 18 h. Then the reaction mixture was stirred at 100 °C for 19 h. Then the mixture was purified (N60, hexane/ethyl acetate, 6:4) to give **Julo-Me** (686 mg, 68%). R_f 0.23 (hexane/ethyl acetate, 1:1); ^{1}H NMR (500 MHz, DMSO, 298 K) δ 7.62

(s, 2H, Ar-H), 6.76 (s, 1H, Vinyl-H), 4.47 (s, 2H, -COCH$_2$-), 3.70 (s, 3H, -OCH$_3$), 3.25 (t, J = 5.8, 4H, -CH$_2$- x2), 2.67 (t, J = 6.3 Hz, 4H, -CH$_2$- x2), 2.25 (s, 3H, -CCH3), 1.90-1.82 (m, 4H, -CH$_2$- x2); ^{13}C NMR (126 MHz, DMSO, 298 K) δ 169.43, 169.06, 168.85, 159.92, 157.95, 144.79, 132.73, 131.61, 127.95, 120.36, 120.22, 99.53, 52.41, 51.90, 49.29, 41.03, 40.84, 27.20, 21.00, 14.97; ESI-TOF-MS: m/z calcd for $C_{20}H_{23}N_3O_3Na^+$: 376.1632 [M+Na]$^+$; found: 376.1641; UV/vis (DMSO): λ_{max} (ε in M^{-1} cm^{-1}) = 494 nm (48700).

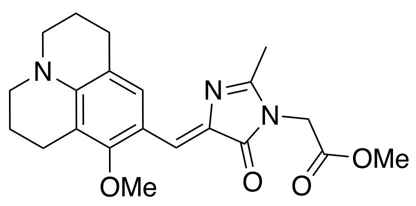

OMeJulo-Me: 8-methoxyjulolidine-9-carboxaldehyde[4] (300 mg, 1.30 mmol) and imidate **1** (525 mg, 3.30 mmol) in toluene (3.6 mL) were stirred at 75 °C overnight. Then the imidate (207 mg, 1.30 mmol) was added and the reaction mixture were further stirred at 75 °C overnight. Then the mixture was purified (N60, hexane/ethyl acetate, 3:2) to give **OMeJulo-Me** (163 mg, 33%). R_f 0.37 (hexane/ethyl acetate, 1:1); ^{1}H NMR (500 MHz, DMSO, 298 K) δ 8.36 (s, 1H, Ar-H), 7.49 (s, 1H, Vinyl-H), 4.40 (s, 2H, -COCH$_2$-), 3.76 (s, 3H, -OCH$_3$), 3.74 (s, 3H, -OCH$_3$), 3.28-3.21 (m, 4H, -CH$_2$- x2), 2.81-2.71 (m, 4H, -CH$_2$- x2) 2.31 (s, 3H, -CH$_3$), 1.98-1.89 (m, 4H, -CH$_2$- x2); ^{13}C NMR (101 MHz, CDCl$_3$, 298 K) δ 168.58, 158.59, 156.72, 130.89, 146.56, 130.89, 124.44, 118.09, 114.33, 113.00, 62.25, 52.81, 50.21, 49.78, 41.41, 27.82, 21.82, 21.37, 21.15, 15.52; ESI-TOF-MS m/z calcd for $C_{21}H_{25}N_3O_4Na^+$:406.1737 [M+Na]$^+$; found: 406.1746.

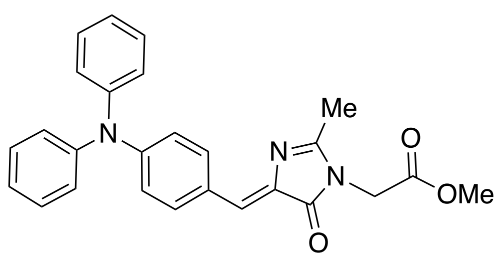

DPhAB-Me: 4-(N,N-diphenylamino)benzaldehyde (782 mg, 2.86 mmol) and imidate **1** (1.00 g, 6.30 mmol) in toluene (5 mL) were stirred at 75 °C for 17 h. Then the reaction mixture was stirred at 100 °C for 19 h. Then the mixture was purified (N60, hexane/ethyl acetate, 3:1) to give **DPhAB-Me** (639 mg, 53%). R_f 0.47 (hexane/ethyl acetate, 1:1); ^{1}H NMR (500 MHz, CDCl$_3$, 298 K) δ 8.00 (d, 2H, J = 8.7 Hz, Ar-H), 7.29 (t, 4H, J = 7.9 Hz, Ar-H), 7.15 (d, J = 7.6 Hz, 4H, Ar-H), 7.12-7.08 (m, 3H, Ar-H, Vinyl-H) 7.02 (d, 2H, J = 8.8 Hz, Ar-H), 4.39 (s, 2H, -COCH$_2$-), 3.78 (s, 3H, -OCH$_3$), 2.30 (s, 3H, -CCH$_3$); ^{13}C NMR (126 MHz, CDCl$_3$, 298 K) δ 170.13, 168.27, 159.49, 149.93, 146.80, 136.03, 133.70, 129.58, 128.47, 127.19, 125.78, 124.34, 121.24, 52.89, 41.41, 15.6; ESI-TOF-MS m/z calcd for $C_{26}H_{23}N_3O_3Na^+$: 448.1632 [M+Na]$^+$; found: 448.1643.

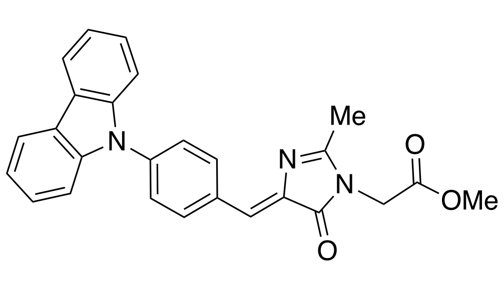

CarbaB-Me: 4-(9H-carbazol-9-yl)benzaldehyde (200 mg, 0.74 mmol) and imidate **1** (258 mg, 1.62 mmol), in toluene (2.1 mL) were stirred at 95 °C for 18 h. Then the mixture was purified (N60, hexane/ethyl acetate, 3:1) to give **CarbaB-Me** (156 mg, 50%). R_f 0.31 (hexane/ethyl acetate, 1:1); ^{1}H NMR (500 MHz, CDCl$_3$, 298 K) δ 8.39 (d, 2H, J = 8.7 Hz, Ar-H), 8.15 (d, 2H, J = 7.7 Hz, Ar-H), 7.67 (d, 2H, J = 8.6 Hz, Ar-H), 7.51 (d, 2H, J = 8.2 Hz, Ar-H), 7.43 (t, 2H, J = 7.1 Hz, Ar-H), 7.31 (t, 2H, J = 6.9 Hz, Ar-H), 7.24 (s, 1H, Vinyl-H), 4.44 (s, 2H, -COCH$_2$-), 3.82 (s, 3H, -OCH$_3$), 2.38 (s, 3H, -CCH$_3$); ^{13}C NMR (126 MHz, CDCl$_3$, 298 K) δ 170.11, 168.12, 161.86, 140.50, 139.41, 138.49, 133.83, 132.95, 127.03, 126.89, 126.22, 123.81, 120.49, 120.47, 110.06, 53.01, 41.46, 15.73; ESI-TOF-MS: m/z calcd for $C_{26}H_{21}N_3O_3Na^+$: 446.1475 [M+Na]$^+$; found: 446.1469.

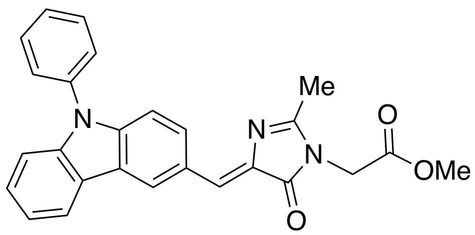

Phcarba-Me: 9-Phenyl-9*H*-carbazole-3-carboxaldehyde (200 mg, 0.74 mmol) and imidate **1** (258 mg, 1.62 mmol) in toluene (2.1 mL) were stirred at 75 °C for 13 h. Then the reaction mixture was further stirred at 100 °C for 15 h. Then the mixture was purified (N60, hexane/ethyl acetate, 2:1) to give **Phcarba-Me** (164 mg, 52%). R_f 0.35 (hexane/ethyl acetate, 1:1); ^{1}H NMR (500 MHz, $CDCl_3$, 298 K) δ 8.96 (s, 1H), 8.28 (d, 1H, J = 8.7 Hz, Ar-H), 8.22 (d, 1H, J = 7.7 Hz, Ar-H), 7.63 (t, 2H, J = 7.8 Hz, Ar-H), 7.57 (d, 2H, J = 7.2 Hz, Ar-H), 7.50 (t, 1H, J = 7.3 Hz, Ar-H), 7.45-7.38 (m, 4H, Ar-H), 7.34 (t, 1H, J = 7.3 Hz, Ar-H), 4.44 (s, 2H, $-COCH_2-$), 3.80 (s, 3H, $-OCH_3$) 2.39 (s, 3H, $-CCH_3$); ^{13}C NMR (126 MHz, $CDCl_3$, 298 K) δ 170.26, 168.31, 159.65, 142.17, 141.62, 137.21, 136.05, 130.93, 130.10, 130.00, 128.02, 127.19, 126.59, 126.47, 125.51, 124.05, 123.56, 120.89, 120.82, 110.27, 110.25, 52.91, 41.46, 15.68; ESI-TOF-MS m/z calcd for $C_{26}H_{21}N_3O_3Na^+$: 446.1475 [M+Na]$^+$; found: 446.1470.

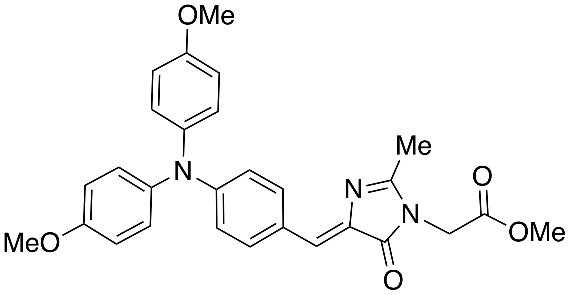

DMeOPhAB-Me: 4-[Bis(4-methoxyphenyl)amino]benzaldehyde (400 mg, 1.20 mmol) and imidate **1** (420 mg, 2.64 mmol) in toluene (3.4 mL) were stirred at 75 °C for 19 h. Then the mixture was purified (N60, hexane : ethyl acetate, 1:1) to give **DMeOPhAB-Me** (413 mg, 71%). R_f 0.16 (hexane/ethyl acetate, 1:1); ^{1}H NMR (500 MHz, $CDCl_3$, 298 K) δ 7.95 (d, 2H, J = 8.9 Hz, Ar-H), 7.13-7.06 (m, 5H, Ar-H, Vinyl-H), 6.89-6.83 (m, 6H, Ar-H), 4.39 (s, 2H, $-COCH_2-$), 3.81 (s, 6H, $-OCH_3$ x2), 3.77 (s, 3H, $-OCH_3$), 2.29 (s, 3H, $-CH_3$); ^{13}C NMR (126 MHz, $CDCl_3$, 298 K) δ 170.13, 168.33, 158.75, 156.87, 150.78, 139.64, 135.28, 133.82, 128.92, 127.68, 125.53, 118.49, 114.96, 55.62, 52.85, 41.41, 15.56; ESI-TOF-MS: m/z calcd for $C_{28}H_{27}N_3O_5Na^+$: 508.1843 [M+Na]$^+$; found: 508.1835.

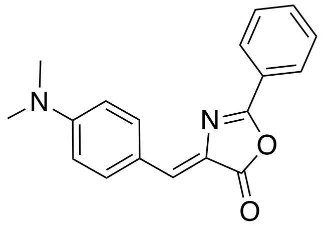

DMAB-Ph-lactone: To a solution of 4-(dimethylamino)benzaldehyde (600 mg, 4.02 mmol) in THF (1 mL), were added hippuric acid (1.35 g, 7.53 mmol), NaOAc (494 mg, 6.02 mmol), Ac_2O (3.0 mL). The reaction mixture was stirred at reflux condition for 1.5 h. The reaction mixture was extracted with CH_2Cl_2 and washed with aq. $NaHCO_3$. The organic layer was evaporated and the residue was purified by column chromatography (60N gel, hexane/ethyl acetate, 3:1, v/v) to give compound **DMAB-Ph-lactone** (867 mg, 74%). R_f 0.71 (hexane/EtOAc 1:1); ^{1}H NMR (500 MHz, $CDCl_3$, 298 K) δ (ppm) 8.17-8.11 (m, 4H, Ar-H), 7.58-7.46 (m, 3H, Ar-H), 7.21 (s, 1H, Vinyl-H), 6.74 (d, J = 8.8 Hz, 2H, Ar-H), 3.10 (s, 6H, $-N(CH_3)_2$); ^{13}C NMR (101 MHz, $CDCl_3$, 298 K) δ (ppm) 168.74, 160.70, 152.37, 135.01, 133.55, 132.44, 128.93, 128.41, 127.90, 126.47, 121.83, 111.88, 40.21; ESI-TOF-MS m/z calcd for $C_{18}H_{16}N_2O_2Na^+$: 315.1104 [M+Na]$^+$; found : 315.1107.

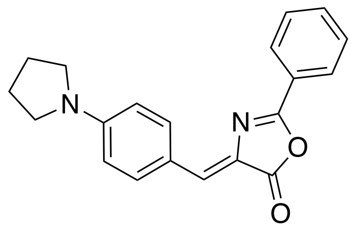

PyrroB-Ph-lactone: To a solution of 4-(pyrrolidine-1-yl)benzaldehyde (1.20 g, 6.86 mmol) in THF (1.7 mL), were added hippuric acid (2.46 g, 13.7 mmol), NaOAc (856 mg, 10.4 mmol), Ac_2O (5.1 mL). The reaction mixture was stirred at 90 °C overnight. The reaction mixture was extracted with CH_2Cl_2 and washed with $NaHCO_3$ aq. and the organic layer was evaporated. The residue was purified by column chromatography (60N gel, hexane/EtOAc, 3:1, v/v) to give compound **PyrroB-Ph-lactone** (1.82 g, 83%). R_f 0.95 (hexane/EtOAc (1:1 v/v)); ^{1}H NMR (500 MHz, $CDCl_3$, 298 K) δ (ppm) 8.17-8.10 (m, 3H, Ar-H), 7.57-7.42 (m, 4H, Ar-H), 7.21 (s, 1H, Vinyl-H), 6.61 (d, J = 8.4 Hz, 2H, Ar-H), 3.44-3.38 (m, 4H, $-N\underline{CH_2}CH_2-$ x2), 2.09-2.03 (m, 4H, $-NCH_2\underline{CH_2}-$ x2); ^{13}C NMR (101 MHz, $CDCl_3$, 298 K) δ (ppm) 168.84, 160.36, 150.05, 135.25, 133.91, 132.30, 128.91, 127.83, 127.80, 126.56, 121.48, 112.13, 47.77, 25.57; ESI-TOF-MS m/z calcd for $C_{20}H_{18}N_2O_2Na^+$: 341.1260 $[M+Na]^+$; found 341.1256.

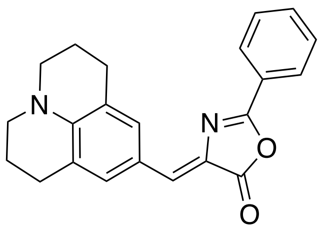

Julo-Ph-lactone: To a solution of 9-julolidine carboxaldehyde (601 mg, 2.99 mmol) in THF (0.75 mL), were added hippuric acid (1076 mg, 6.00 mmol), NaOAc (379 mg, 4.62 mmol), Ac_2O (2.3 mL). The reaction mixture was stirred at reflux condition for 4 h. The reaction mixture was extracted with CH_2Cl_2 and washed with $NaHCO_3$ aq. and the organic layer was evaporated. The residue was purified by column chromatography (60N gel, hexane/EtOAc, 3:1, v/v) to give compound **Julo-Ph-lactone** (698 mg, 68%). R_f 0.74 (hexane/EtOAc (1:1 v/v)); ^{1}H NMR (500 MHz, $CDCl_3$, 298 K) δ (ppm) 8.12 (d, J = 6.7 Hz, 2H, Ar-H), 7.70 (br, 2H, Ar-H), 7.55-7.45 (m, 3H, Ar-H), 7.11 (s, 1H, Vinyl-H), 3.32 (t, J = 5.8 Hz, 4H, $-N\underline{CH_2}CH_2CH_2-$ x2), 2.80 (t, J = 6.3 Hz, 4H, $-NCH_2CH_2\underline{CH_2}-$ x2), 1.98 (quin, J = 6.0 Hz, 4H, $-NCH_2\underline{CH_2}CH_2-$ x2); ^{13}C NMR (101 MHz, $CDCl_3$, 298 K) δ (ppm) 168.96, 159.81, 146.25, 134.12, 132.72, 132.11, 128.88, 127.71, 127.06, 126.66, 121.16, 120.82, 50.28, 27.91, 21.50; ESI-TOF-MS m/z calcd for $C_{22}H_{21}N_2O_2^+$: 345.1598 $[M+H]^+$; found: 345.1607.

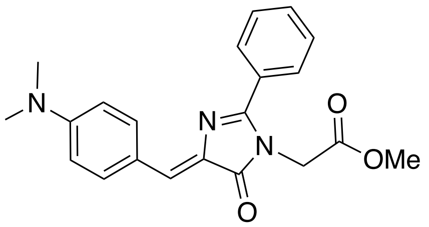

DMAB-Ph: To a solution of compound **DMAB-Ph-lactone** (859 mg, 2.94 mmol) in THF (16 mL), were added glycine methyl ester hydrochloride (814 mg, 6.48 mmol), triethylamine (1.8 mL, 12.9 mmol). The reaction mixture was stirred at reflux condition for 5 h. The reaction mixture was evaporated. To the residue, was added dry pyridine (16 mL) and the reaction mixture was stirred at reflux condition for 17 h. Then, the reaction mixture was evaporated and the residue was purified by column chromatography (60N gel, hexane/EtOAc, 3:2, v/v) to give compound **DMAB-Ph** (132 mg, 12%). R_f 0.56 (hexane/EtOAc (1:1 v/v)); ^{1}H NMR (500 MHz, $CDCl_3$, 298 K) δ (ppm) 8.17 (d, J = 8.7 Hz, 2H, Ar-H), 7.71 (d, J = 7.8 Hz, 2H, Ar-H), 7.54-7.47 (m, 3H, Ar-H), 7.27 (s, 1H, Vinyl-H), 6.71 (d, J = 8.9 Hz, 2H, Ar-H), 4.52 (s, 2H, $-COCH_2-$), 3.71 (s, 3H, $-OCH_3$), 3.07 (s, 6H, $-N(CH_3)_2$); ^{13}C NMR (101 MHz, $CDCl_3$, 298 K) δ (ppm) 171.08, 168.72, 158.05, 151.99, 135.07, 134.33, 131.60, 131.04, 129.84, 129.07, 128.79, 128.39, 127.20, 122.39, 111.86, 52.78, 43.22, 40.20; ESI-TOF-MS m/z calcd for $C_{21}H_{21}N_3O_3Na^+$: 386.1475 $[M+Na]^+$; found : 386.1483.

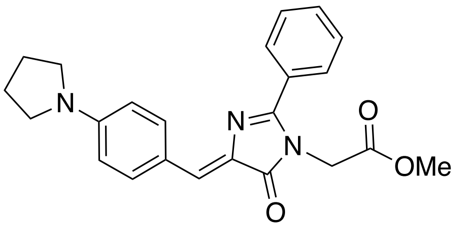

PyrroB-Ph: To a solution of compound **PyrroB-Ph-lactone** (980 mg, 3.08 mmol) in THF (20 mL), were added glycine methyl ester hydrochloride (604 mg, 4.81 mmol), triethylamine (2 mL, 14.3 mmol). The reaction mixture was stirred at reflux condition for 16 h. The reaction mixture was evaporated. To the residue, was added dry pyridine (10 mL) and the reaction mixture was stirred at reflux condition for 19 h. Then, the reaction mixture was evaporated and the residue was purified by column chromatography (60N gel, hexane/EtOAc, 3:2, v/v) to give compound **PyrroB-Ph** (460 mg, 38%). R_f 0.56 (hexane/EtOAc (1:1 v/v)); ^{1}H NMR (500 MHz, $CDCl_3$, 298 K) δ (ppm) 8.17 (d, J = 7.8 Hz, 2H, Ar-H), 7.73-7.68 (m, 2H, Ar-H), 7.53-7.46 (m, 3H, Ar-H), 7.27 (s, 1H, Vinyl-H), 6.58 (d, J = 8.6 Hz, 2H, Ar-H), 4.52 (s, 2H, $-COCH_2-$), 3.71 (s, 3H, $-OCH_3$) 3.42-3.36 (m, 4H, $-N\underline{CH_2}CH_2-$ x2) 2.06-2.02 (m, 4H, $-NCH_2\underline{CH_2}-$ x2); ^{13}C NMR (101 MHz, $CDCl_3$, 298 K) δ (ppm) 171.05, 168.77, 157.61, 149.65, 135.33, 133.81, 132.01, 130.96, 129.93, 129.05, 128.38, 121.97, 112.02, 52.77, 47.70, 43.22, 25.58; ESI-TOF-MS m/z calcd for $C_{23}H_{23}N_3O_3Na^+$: 412.1632, $[M+Na]^+$; found: 412.1640; UV/vis (DMSO): λ_{max} (ε in M^{-1} cm^{-1}) = 480 nm (32800).

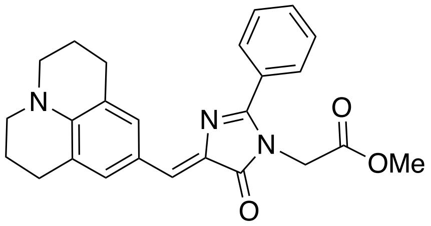

Julo-Ph: To a solution of compound **Julo-Ph-lactone** (2.15 g, 6.24 mmol) in THF (40 mL), were added glycine methyl ester hydrochloride (1.18 g, 9.40 mmol), triethylamine (3.8 mL, 27.3 mmol). The reaction mixture was stirred at reflux condition for 16 h. The reaction mixture was evaporated. To the residue, was added dry pyridine (30 mL) and the reaction mixture was stirred at reflux condition for 22 h. Then, the reaction mixture was evaporated and the residue was purified by column chromatography (60N gel, hexane/EtOAc, 1:1, v/v) to give compound **Julo-Ph** (468 mg, 18%). R_f 0.29 (hexane/EtOAc (1:1 v/v)); ^{1}H NMR (500 MHz, DMSO, 298 K) δ (ppm) 7.79-7.69 (m, 4H, Ar-H), 7.61-7.52 (m, 3H, Ar-H), 6.97 (s, 1H, Vinyl-H), 4.58 (s, 2H, $-COCH_2-$), 3.60 (s, 3H, $-OCH_3$), 3. 28 (t, J = 5.5 Hz, 4H, $-N\underline{CH_2}CH_2CH_2-$ x2), 2.69 (t, J = 5.9 Hz, 4H, $-NCH_2CH_2\underline{CH_2}-$ x2) 1.91-1.83 (m, 4H, $-NCH_2\underline{CH_2}CH_2-$ x2); ^{13}C NMR (126 MHz, DMSO, 298 K) δ (ppm) 169.69, 168.80, 156.80, 145.36, 132.51, 132.23, 130.87, 130.26, 129.43, 128.98, 127.96, 120.54, 120.29, 52.39, 49.38, 42.76, 27.24, 20.92; ESI-TOF-MS m/z calcd for $C_{25}H_{25}N_3O_3Na^+$: 438.1788, $[M+Na]^+$; found: 438.1794; UV/vis (DMSO): λ_{max} (ε in M^{-1} cm^{-1}) = 461 nm (35500).

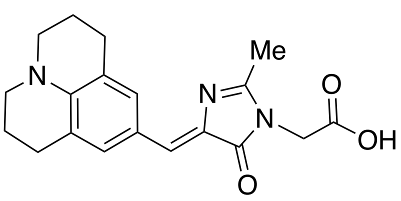

Julo-Me-COOH: The compound **Julo-Me** (110 mg 0.31 mmol) in CH_2Cl_2/MeOH (3 mL) was treated with 1 M NaOH in MeOH (0.62 mL 0.62 mmol) at room temperature for 26 h. Then, To the reaction mixture was added 1 M HCl in MeOH (1.0 mL). The reaction mixture was evaporated and applied to short column chromatography (N60, CH_2Cl_2/MeOH, 2:1, v/v) to give crude product (95 mg). The obtained crude product was directly used for solid phase synthesis. HRMS (ESI): m/z calcd for $C_{19}H_{20}N_3O_3^-$: 338.1510, $[M-H]^-$; found: 338.1501.

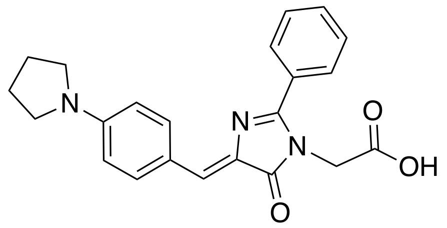

PyrroB-Ph-COOH: The compound **PyrroB-Ph** (200 mg 0.513 mmol) in CH_2Cl_2/MeOH (5:1, v/v, 1.2 mL) was treated with 1 M NaOH in MeOH (1 mL 1.0 mmol) at room temperature for 3 h. Then, To the reaction mixture was added 0.5 M HCl (50 mL). The reaction mixture was extracted with CH_2Cl_2. The organic layer was evaporated to give crude product (188 mg). The obtained crude product was directly used for solid phase synthesis. HRMS (ESI): m/z calcd for $C_{22}H_{20}N_3O_3$-: 374.1510, $[M-H]^-$; found: 374.1656.

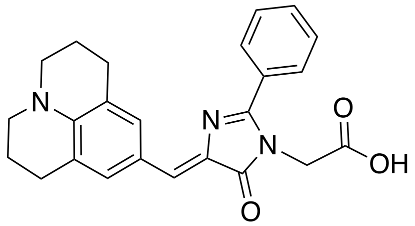

Julo-Ph-COOH: The compound **Julo-Ph** (70 mg, 0.17 mmol) in CH_2Cl_2/MeOH (1:1, v/v, 2 mL), was treated with 1 M NaOH in MeOH (0.34 mL, 0.34 mmol) at room temperature for 20 h. Then, To the reaction mixture was added 0.1 M HCl and extracted with CH_2Cl_2. The organic layer was evaporated to give crude product (41 mg). The obtained crude product was directly used for solid phase synthesis. HRMS (ESI): m/z calcd for $C_{24}H_{22}N_3O_3^-$: 400.1667, $[M-H]^-$; found: 400.1667.

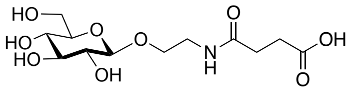

Glc-COOH: To a solution of 2-aminoethyl *β*-D-glucopyranoside[5] (461 mg 2.07 mmol) in DMF (20 mL), was added succinic anhydride (269 mg, 2.69 mmol). The reaction mixture was stirred at room temperature for 2 days. Then, To the reaction mixture, was added MeOH and the reaction mixture was evaporated. The residue was purified by column chromatography (60N gel, CH_2Cl_2/MeOH, 4:1, v/v) to give compound **Glc-COOH** (410 mg, 61%). R_f 0.58 (CH_2Cl_2/MeOH/AcOH 3:1:0.5 v/v)); 1H NMR (500 MHz, Methanol-*d4*) δ 4.29 (d, J = 7.8 Hz, 1H), 3.94–3.84 (m, 2H), 3.70–3.63 (m, 2H), 3.48–3.42 (m, 1H), 3.39–3.33 (m, 2H), 3.30–3.26 (m, 2H), 3.23–3.15 (m, 1H), 2.60–2.54 (m, 2H), 2.51–2.46 (m, 2H); ^{13}C NMR (126MHz, Methanol-*d4*, 298K) δ (ppm) 104.53, 77.97, 75.14, 71.63, 69.68, 62.72, 40.70, 31.96, 31.01; HRMS (ESI): m/z calcd for $C_{12}H_{21}NO_9Na^+$: 364.1109, $[M+Na]^+$; found: 364.1103.

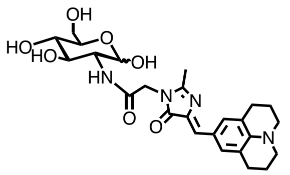

GlcN-Julo-Me: **Julo-Me-COOH** (30.0 mg, 88.4 μmol), 1-(3-dimethylaminopropyl)-3-ethylcarbodiimide hydrochloride (33.0 mg, 213 μmol), 1-hydroxybenzotriazole (28.6 mg, 213 μmol) in DMF (1 mL) were stirred at room temperature for 15 min. Then, 2-amino-2-deoxy-*β*-D-glucopyranose hydrochloride (38.2 mg, 177 μmol) and NaHCO3 (37.0 mg, 426 μmol) were added to the reaction mixture and stirred for 17 h. Then, the mixture was quenched by adding 400 μL water and extracted with DCM containing MeOH. The organic layer was concentrated, and the residue was purified by reverse phase HPLC (water/MeCN) to give compound **GlcN-Julo-Me** (21.9 μmol (determined by UV-absorption), 25%). ESI-TOF-MS: m/z calcd for $C_{25}H_{32}N_4O_7Na^+$:523.2163, $[M+Na]^+$; found: 523.2166.

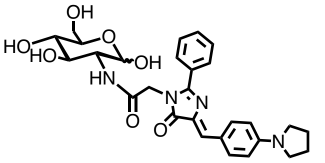

GlcN-PyrroB-Ph: **PyrroB-Ph-COOH** (30.2 mg, 80.4 μmol), 1-(3-dimethylaminopropyl)-3-ethylcarbodiimide hydrochloride (26.1 mg, 168 μmol), 1-hydroxybenzotriazole (23.1 mg, 171 μmol) in DMF (1 mL) were stirred at room temperature for 15 min. Then, 2-amino-2-deoxy-*β*-D-glucopyranose hydrochloride (35.9 mg, 167 μmol) and $NaHCO_3$ (35.8 mg, 426 μmol) were added to the reaction mixture and stirred for 17 h. Then, the mixture was quenched by adding 400 μL water and extracted with DCM containing MeOH. The organic layer was concentrated, and the residue was purified by reverse phase HPLC (water/MeCN) to give compound **GlcN-PyrroB-Ph** (13.4 μmol (determined by UV-absorption), 17%). ESI-TOF-MS: m/z calcd for C28H32N4O7Na$^+$:559.2163, [M+Na]$^+$; found: 559.2156.

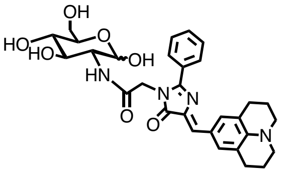

GlcN-Julo-Ph: **Julo-Ph-COOH** (150 mg, 374 μmol), 1-(3-dimethylaminopropyl)-3-ethylcarbodiimide hydrochloride (116 mg, 747 μmol), 1-hydroxybenzotriazole (101 mg, 747 μmol) in DMF (1 mL) were stirred at room temperature for 5 min. Then, 2-amino-2-deoxy-*β*-D-glucopyranose hydrochloride (161 mg, 747 μmol) and $NaHCO_3$ (157 mg, 1869 μmol) were added to the reaction mixture and stirred for 18 h. Then, the mixture was diluted with DCM and concentrated, and the residue was purified by medium-pressure column chromatography system on a Purif-Compact and ODS silica gel with water/MeCN solvent system to give compound **GlcN-Julo-Ph** (53 mg, 25%). ESI-TOF-MS: m/z calcd for $C_{30}H_{34}N_4O_7Na^+$: 585.2320, [M+Na]$^+$; found: 585.2329.

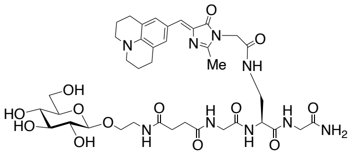

Glc-Pep-Julo-Me: The compound was synthesized via SPPS with **Julo-Me-COOH** and **Glc-COOH** as described in experimental section. HRMS (ESI): m/z calcd for $C_{38}H_{53}N_9O_{13}Na^+$: 866.3655, [M+Na]$^+$; found: 866.3669.

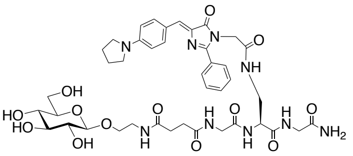

Glc-Pep-PyrroB-Ph: The compound was synthesized via SPPS with **PyrroB-Ph-COOH** and **Glc-COOH** as described in experimental section. HRMS (ESI): m/z calcd for $C_{41}H_{53}N_9O_{13}Na^+$: 902.3655, [M+Na]$^+$; found: 902.3662.

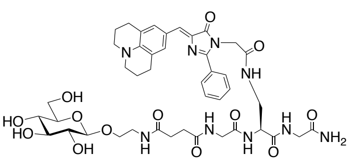

Glc-Pep-Julo-Ph: The compound was synthesized via SPPS with **Julo-Ph-COOH** and **Glc-COOH** as described in experimental section. HRMS (ESI): m/z calcd for $C_{43}H_{55}N_9O_{13}Na^+$: 928.3812, [M+Na]$^+$; found: 928.3826.

Photophysical Measurements

(1) UV–Vis and Fluorescence Spectra

Each fluorophore was dissolved in DMSO to prepare a 10 mM stock solution. From this stock, 15 μM solutions were prepared in methanol, glycerol, and methanol/glycerol mixtures, each containing 0.15% (v/v) DMSO. UV–Vis spectra were recorded on a UV-2600 spectrophotometer (Shimadzu) using a 1 cm pathlength quartz cuvette. Steady-state fluorescence spectra were recorded on a FP-8500 spectrofluorometer (JASCO) using a rectangular cuvette (1.0 cm × 0.3 cm); samples were positioned so that the excitation pathlength was 0.3 cm.

(2) Viscosity-Dependent Fluorescence

Stock solutions of the GFP-derived fluorophores (15 μM) were prepared in glycerol and methanol. Mixed solvents of methanol/glycerol were then prepared at 40%, 60%, 80%, and 100% (v/v) glycerol. Aliquots (150 μL) were dispensed into a 96-well plate, and fluorescence was recorded on a Varioskan LUX plate reader (Thermo Fisher; model VLBL00D0). Instrument settings were as follows: measurement time 100 ns, excitation bandwidth 12 nm, top-read mode. Each fluorophore was excited at its absorption maximum measured in glycerol (0.15% DMSO), and emission spectra were collected from 450 to 650 nm.

Quantum Chemical Calculations

All calculations were performed with Gaussian 16, Revision B.01.[6] Ground-state (S_0) geometries were optimized in methanol using the IEFPCM solvent model, the hybrid B3LYP functional, and the 6-31+G(d) basis set. During the geometry optimization, only the specified dihedral angle was constrained, while all other degrees of freedom were optimized without restraints. Excited-state (S_1) optimizations were carried out by TD-DFT at the same B3LYP/6-31+G(d)/IEFPCM(CH_3OH) level. Where necessary, the quadratically convergent (QC) SCF procedure was employed to improve convergence. The S_1 and S_0 molecular orbitals of each optimized structure were visualized based on DFT calculations performed in the same manner as described above.

Molecular Docking Simulations

Docking simulations of **GlcN-Julo-Ph** were performed with the Glide algorithm in the Schrödinger Maestro suite. The human GLUT3 structure (PDB ID: 4ZW9) was downloaded from the RCSB database and prepared with Protein Preparation Wizard. Ligand structures were prepared using LigPrep. Standard Precision (SP) Glide docking was carried out, and binding affinities were reported as Glide scores.

Synthetic protocols of GFP fluorophore-modified probes

(1) Solid phase peptide synthesis (SPPS) protocols

Following the scheme in **Scheme S3**, peptide probes were assembled on NovaSyn TGR resin (100 mg) in a 10 mL syringe. The resin was swollen in DMF and dichloromethane. For each coupling cycle, the corresponding Fmoc-amino acid (3 equiv), PyBOP (3 equiv), N-methylmorpholine (NMM, 6 equiv), and DMF (250 μL) were added and the mixture was shaken at r.t. for 1 h. The resin was washed with DMF and dichloromethane, then capped with Ac_2O/2,6-lutidine/DMF (5:6:89, v/v, 500 μL, 5 min), followed by washing. Fmoc deprotection was carried out with piperidine/DMF (1:4, v/v, 500 μL, 2 min); the treatment was repeated once more (2 min) and the resin was washed thoroughly. This cycle was repeated to elongate the full length peptide.

Subsequently, **Glc-COOH** (1.5 equiv), PyBOP (3 equiv), NMM (6 equiv), and DMF (250 μL) were added and the mixture was shaken at r.t. for 1 h. After washing, hydrazine/DMF (1:24, v/v, 500 μL) was added to remove the Dde protecting group (5 min, shaking), and this deprotection was repeated two times. After washing, each GFP fluorophore unit (1.5 equiv) was coupled with PyBOP (3 equiv) and NMM (6 equiv) in DMF (250 μL) at r.t. for 1 h. The resin was washed, and the product was cleaved with TFA/TIS/H_2O (38:1:1, v/v) for 1 h with shaking. The filtrate was concentrated in a 10 mL round-bottom flask and precipitated with ice-cold diethyl ether. The precipitate was collected by centrifugation (7000 rpm, 5 min, 4 °C) the supernatant was removed, and the pellet was dissolved in DMSO (100 μL), then diluted with Milli-Q water to obtain the crude solution.

(2) Purification of the Peptide-Backbone Probe and HPLC Analysis

The crude peptide-backbone probe was dissolved in water containing 50% acetonitrile and filtered through a hydrophilic PTFE syringe filter (0.45 μm). The solution was loaded onto an HPLC system (Shimadzu). Buffer A was 99% water with 1% acetonitrile, and Buffer B was 99% acetonitrile with 1% water. A linear gradient from 0% to 60% B over 30 min was applied; the column temperature was 30 °C. Analysis and fraction collection were performed at each maximum absorption wavelength (~480 nm). Solvents were removed by centrifugal evaporation. The collected material was dissolved in DMSO and diluted with water, then analyzed by HPLC under the same buffers and gradient conditions.

(3) Preparation of Glucosamine-Backbone Probe Solutions

A 2 mM DMSO stock of the glucosamine-backbone probe was prepared. To suppress precipitation, RPMI-1640 medium (glucose-free, L-glutamine-containing) and the probe stock were aliquoted into 1.5 mL tubes and heated on a heat block (80 °C, 5 min). The hot probe stock was then diluted 100-fold with hot RPMI-1640 to prepare a 20 μM working solution. For GLUT-inhibition experiments, probe stocks were diluted with RPMI-1640 containing each inhibitor.

Cellular experiment protocols

(1) Cell Culture

PC-3 cells maintained in RPMI-1640 (2.0 g/L glucose; Nacalai Tesque, 30264–56) supplemented with 10% FBS and 1% Antibiotic-Antimycotic (ABAM) were used. First, culture medium was aspirated, cells were washed with PBS (3 mL), and trypsin in PBS (1:1, v/v, 1 mL) was added and incubated for 3 min (37 °C, 5% CO_2). Then, RPMI (3 mL)

was added, the suspension was transferred to a 15 mL tube, and centrifuged (1200 rpm, 5 min, r.t.). The supernatant was removed, RPMI (2 mL) was added, and the pellet was resuspended by pipetting. A 10 µL aliquot was mixed with 10 µL trypan blue and counted on a Countess II FL. Cells were adjusted to 2.5 × 10^5 cells/mL and seeded at 132 µL/well into 35 mm multi-well glass-bottom dishes (Matsunami Glass, D141400). Cells were incubated overnight (37 °C, 5% CO_2) and used in the experiment on the following day.

(2) Live-Cell Imaging

PC-3 cells were seeded at 2.5 × 10^5 cells/mL in multi-well glass-bottom dishes and incubated overnight. The next day, medium was removed, and cells were washed with PBS (100 µL). RPMI-1640 (glucose-free, L-glutamine-containing; Nacalai Tesque, 09892-15) (100 µL) was added and cells were incubated at r.t. for 30 min. The medium was then removed, the GFP fluorophore probes solution diluted in RPMI-1640 was added, and cells were incubated at r.t. for 30–60 min before imaging on an inverted confocal laser-scanning microscope (Carl Zeiss LSM780). For the imaging of 2-NBDG (Peptide Institute, 23002-v) treated cells, 2-NBDG stock (50 mM in DMSO) was diluted with RPMI-1640 or DMEM as required and used following the same protocol as the GFP fluorophore probes. As indicated in the figure legends, imaging was performed with or without washing. For washing, cells were rinsed three times with PBS (100 µL each), then 100 µL of glucose-free medium was added for confocal imaging. Bright-field images were acquired in T-PMT mode. Fluorescence images were excited with the built-in 488 nm diode laser, and emission (490–624 nm) was collected using the built-in GaAsP detector.

(3) GLUT Inhibition Experiments

PC-3 cells were seeded at 2.5 × 10^5 cells/mL and incubated overnight. After removal of medium and a PBS wash (100 µL), solutions of D-glucose (FUJIFILM Wako, 044-00605), phloretin (TCI, P1966; 60 mM in DMSO), or cytochalasin B (FUJIFILM Wako, 036-17553; 20 mM in DMSO) were prepared in glucose-free RPMI-1640 at the indicated concentrations, and 100 µL was added to cells. After 30 min at r.t., the medium was removed and 100 µL of the fluorescent probe solution (from a 2 mM DMSO stock) containing each inhibitor was added. Cells were incubated at r.t. for 30 or 60 min and imaged on a confocal microscope.

(4) Fluorescence Imaging with RAW264.7 Cells

RAW264.7 cells maintained in DMEM (1.0 g/L glucose; Nacalai Tesque, 08456-65) supplemented with 10% FBS and 1% ABAM were used. Subsequent procedures were identical to those described for PC-3 cells, except that DMEM was used instead of RPMI-1640.

(5) Quantification of Fluorescence Intensity of Cell Imaging

Images were acquired with Carl Zeiss ZEN 2.3 (lite). Original data were stored as 16-bit images (gray-level range 0–65535). For all images, the contrast was adjusted so that the range between the minimum and maximum intensity values was 22,500. For the quantification, cell segmentation was performed with Cellpose 2.0 (general model). Fluorescence intensities at each cell were quantified by ImageJ 1.53t (NIH).

(6) Immunostaining

PC-3 cells were seeded at 2.5 × 10^5 cells/mL and incubated overnight. After removal of medium and a PBS wash (100 µL), cells were fixed with 4% formaldehyde in PBS (100 µL, 30 min, rt). After three times PBS washes (100 µL each), blocking was performed with 5% BSA in PBS (100 µL, 1 h, 37 °C, 5% CO_2). After three times PBS washes, cells were incubated with anti-GLUT1 antibody (EPR3915, abcam, ab115730) diluted 1:400 in 1% BSA/PBS (100 µL) for 45 min (37 °C, 5% CO_2), washed three times with PBS, and then incubated with a secondary antibody (Goat anti-Rabbit IgG (H+L), Alexa Fluor™ Plus 647; Invitrogen, Thermo Fisher Scientific, A32733), 1:2500 in 1% BSA/PBS, 100 µL, 45 min, 37 °C, 5% CO_2). After three times PBS washes, RPMI-1640 (100 µL) was added and cells were imaged by confocal microscopy. Fluorescence was excited with the 633 nm diode laser, and emission 638–756 nm was collected using the GaAsP detector.

(7) Colocalization Studies

Cells were co-stained with Hoechst 33342 and LysoTracker™ Deep Red (Invitrogen) with a standard method. Colocalization between LysoTracker Deep Red and the **GlcN-Julo-Ph**-derived fluorescence signals was analyzed in Fiji (ImageJ). Colocalization was analyzed for the white-outlined ROI using a standard method.

(8) MTT Assay

PC-3 or RAW264.7 cells were seeded in 96-well plates at 1.0 × 10^5 cells/mL and incubated overnight (37 °C, 5% CO_2). The next day, medium was removed, and cells were treated with **GlcN-Julo-Ph** or **2-NBDG** dissolved in glucose-free medium at the indicated concentrations and incubated for 24 h (37 °C, 5% CO_2). After incubation, medium was removed, and cells were washed with PBS (100 µL). Then, normal medium (100 µL) was added, followed by MTT reagent (10 µL, 10% in PBS), and incubation proceeded for 4 h (37 °C, 5% CO_2). SDS (100 µL, 10% in PBS) was then added and the plate was incubated overnight (37 °C, 5% CO_2). The next day, absorbance at 570 nm was measured on a plate reader, and cell viability was calculated from the absorbance values.

2. Supplemental Schemes and Figures

Synthetic Scheme of fluorophore monomers

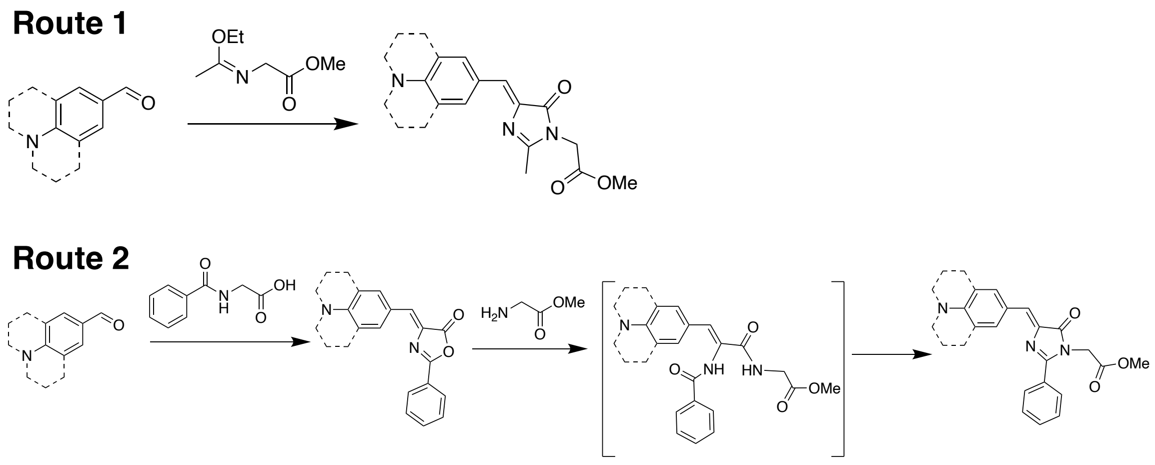

Scheme S1. Synthetic scheme for GFP analogues used in this study.

UV and fluorescence spectra

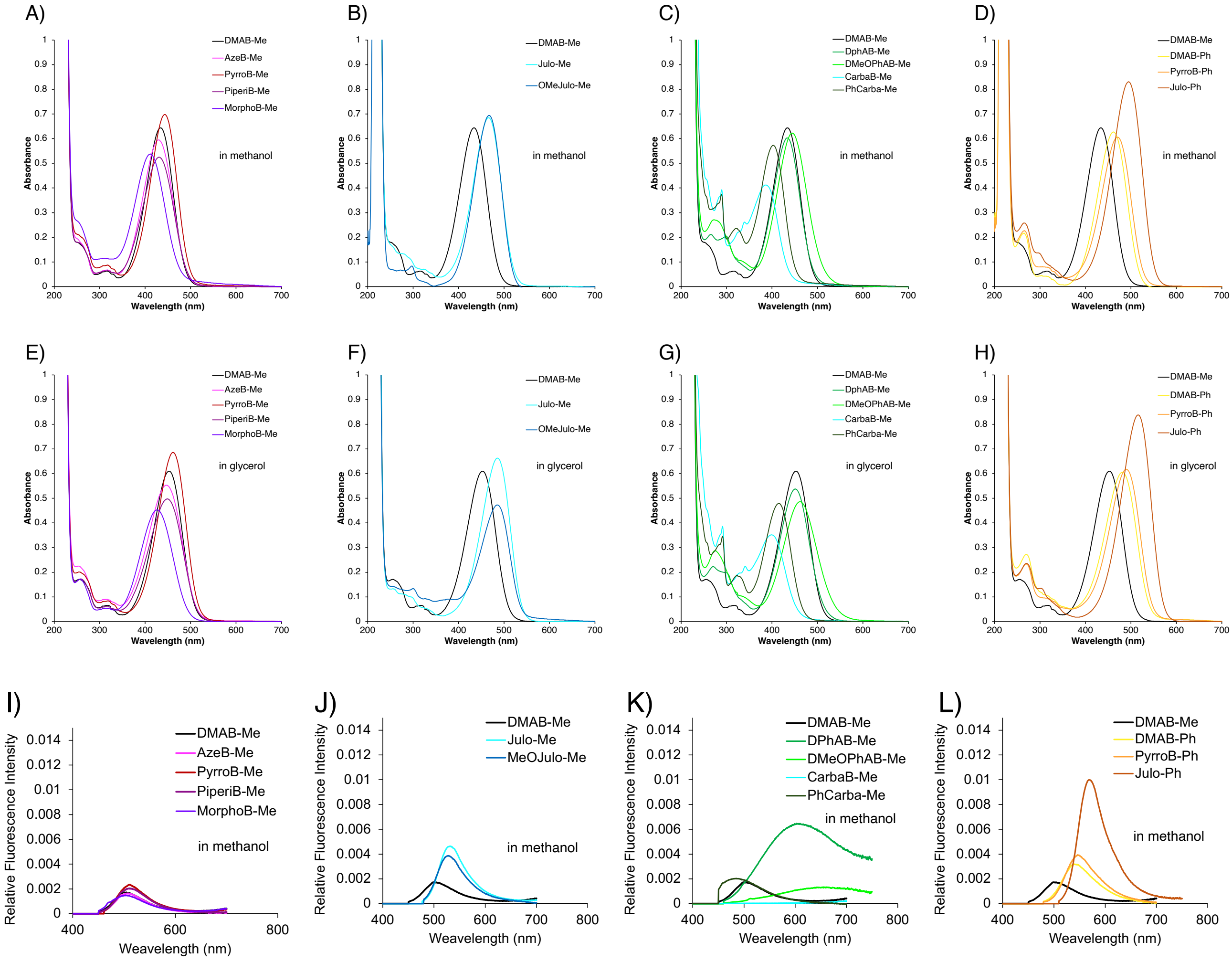

Figure S1. Absorption spectra of the synthesized DMAB derivatives (15 μM with 0.15% DMSO) in methanol [A) ~ D)] and glycerol [E) ~ H)], and fluorescence spectra of the synthesized DMAB derivatives (15 μM with 0.15% DMSO) in methanol [I) ~L)].

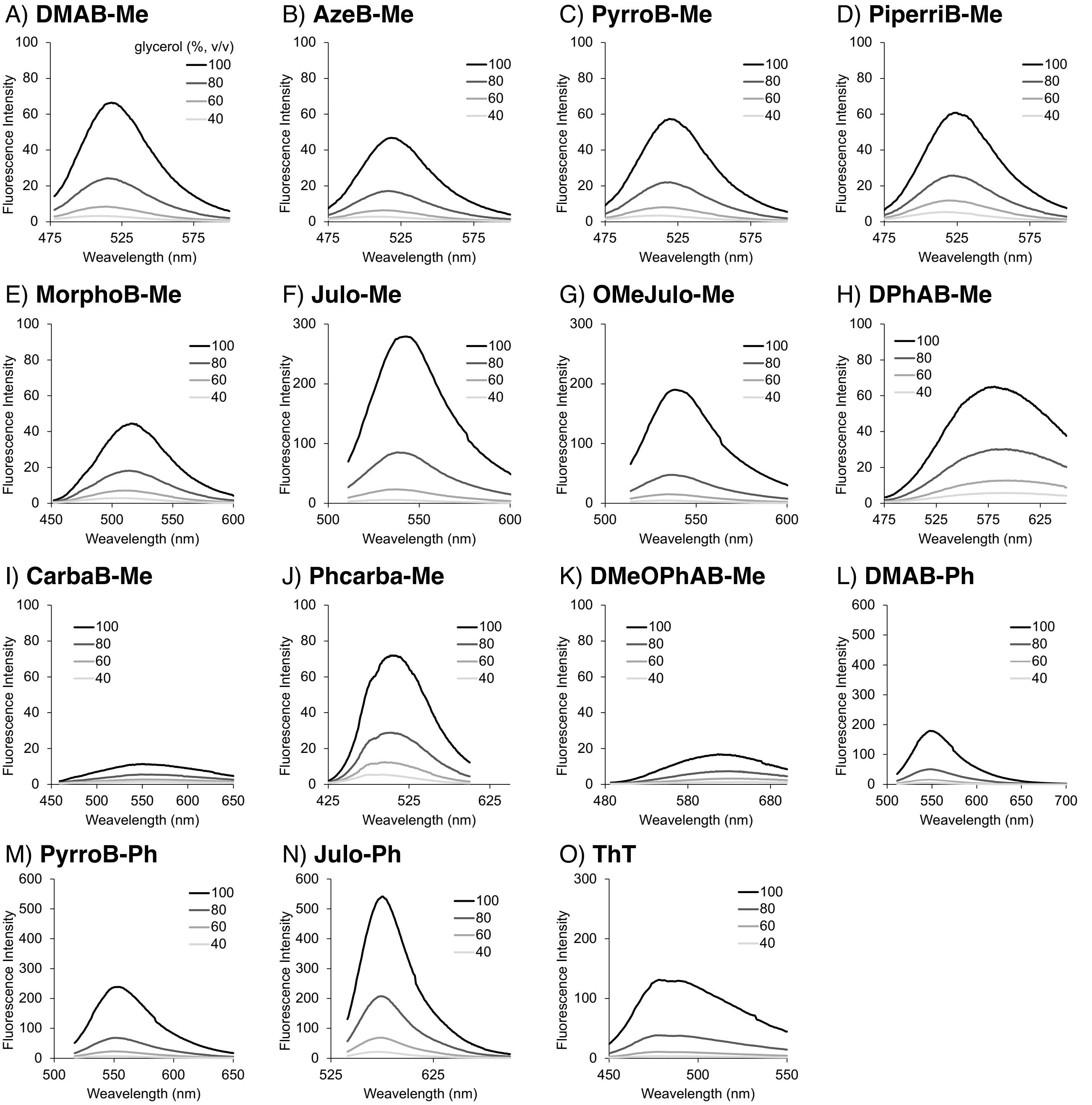

Figure S2. Fluorescence spectra of A) ~ N) DMAB derivatives (15 μM with 0.15% DMSO) and O**) ThT** in glycerol-methanol mixed solvent (100%, 80%, 60%, and 40% glycerol, v/v) measured by using fluorescent microplate reader.

Optimized structures obtained by DFT and TD-DFT calculations

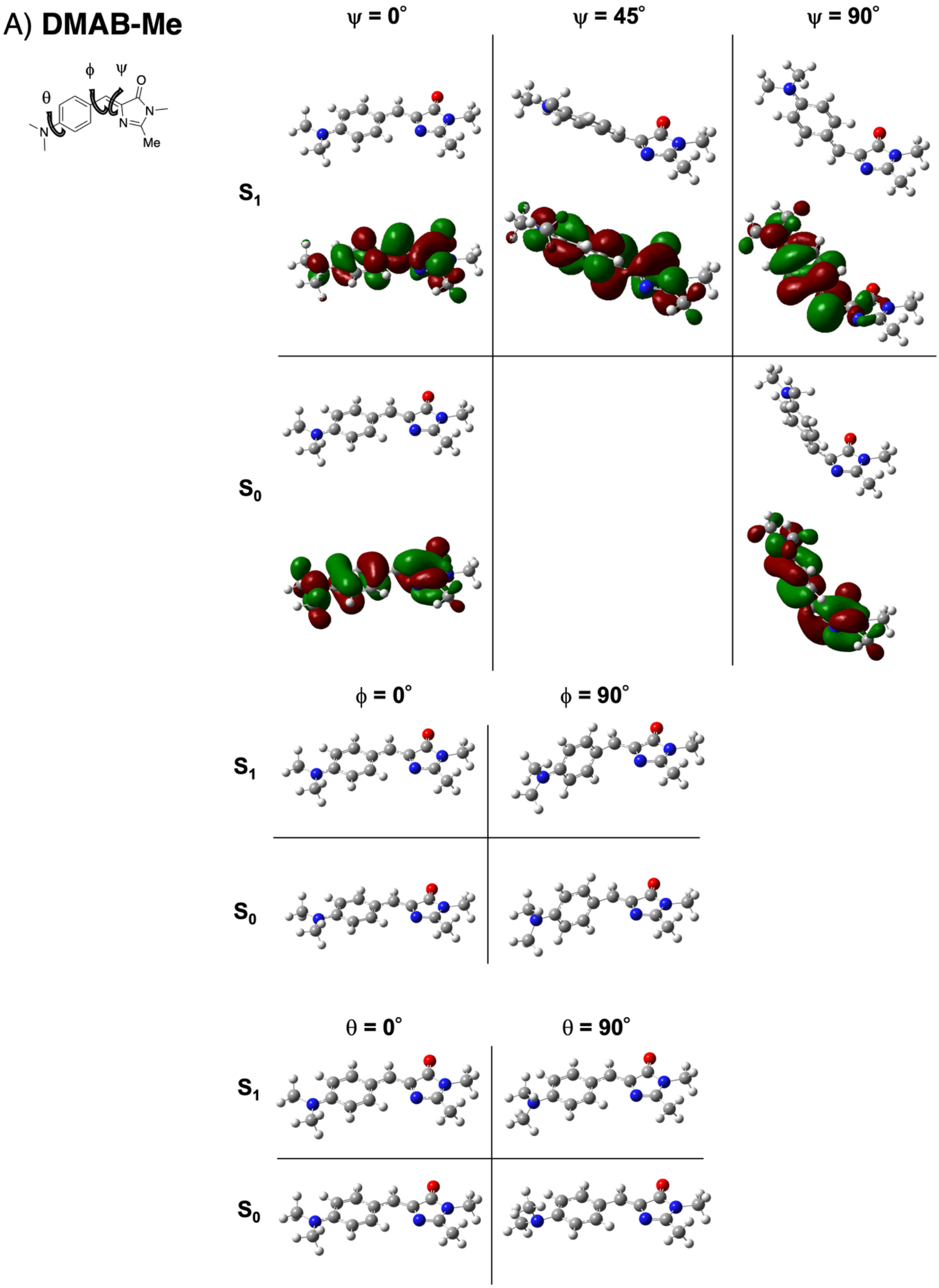

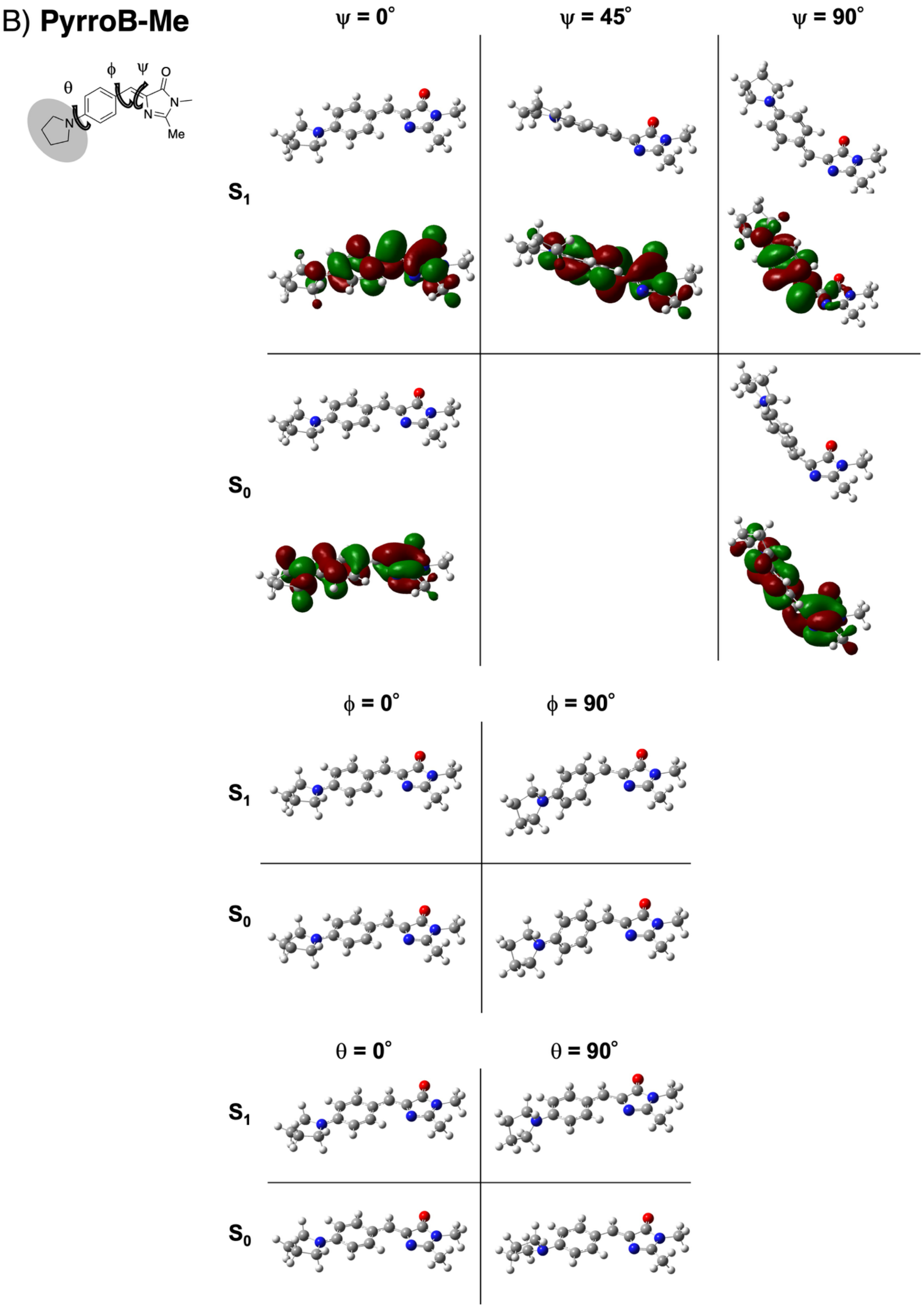

B) PyrroB-Me
θ
ϕ
ψ
O
N
N
Me
ψ = 0°
ψ = 45°
ψ = 90°
S1
S0
ϕ = 0°
ϕ = 90°
S1
S0
θ = 0°
θ = 90°
S1
S0

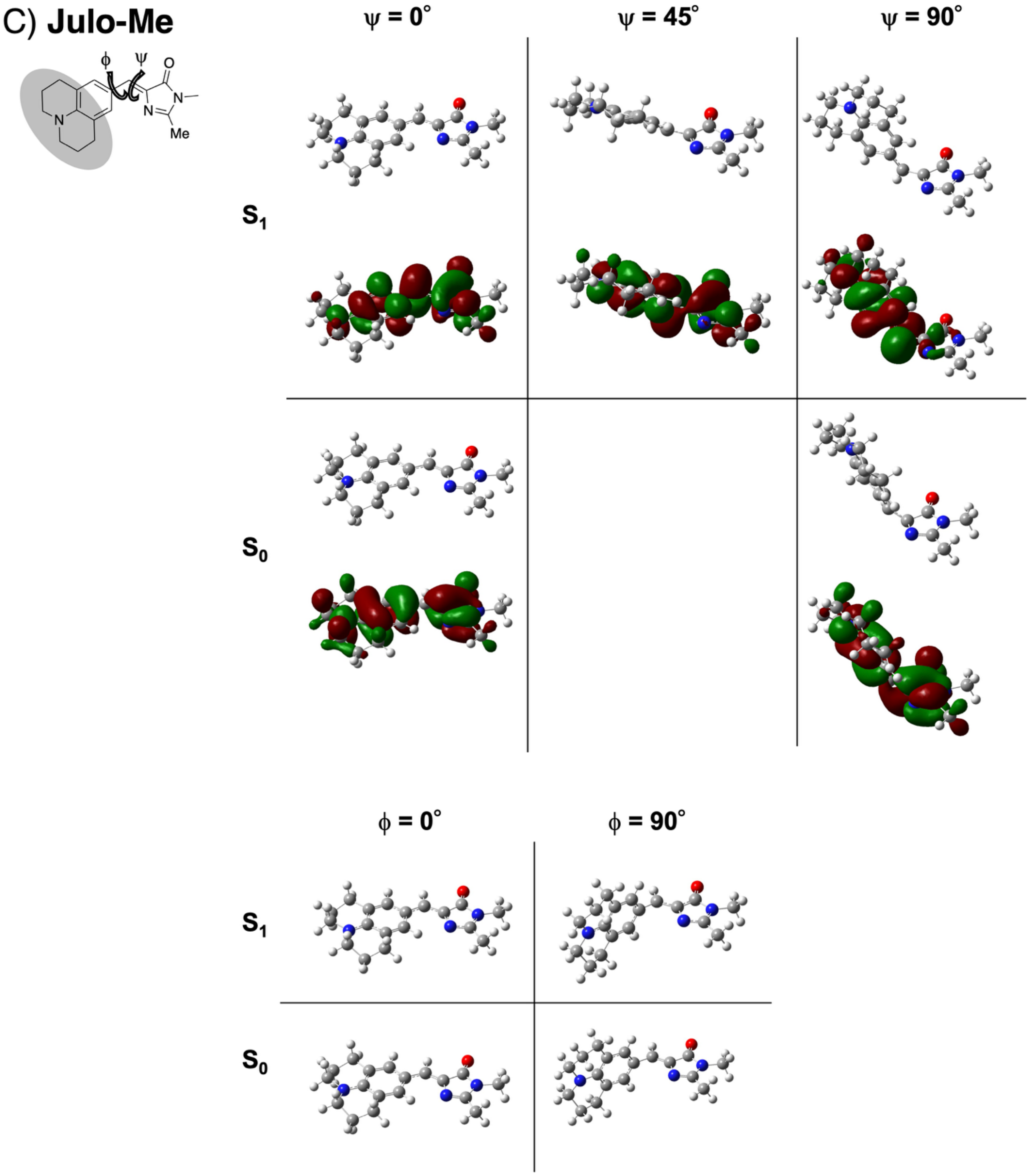
C) Julo-Me
ϕ
ψ
O
N
N
Me
ψ = 0°
ψ = 45°
ψ = 90°
S1
S0
ϕ = 0°
ϕ = 90°
S1
S0

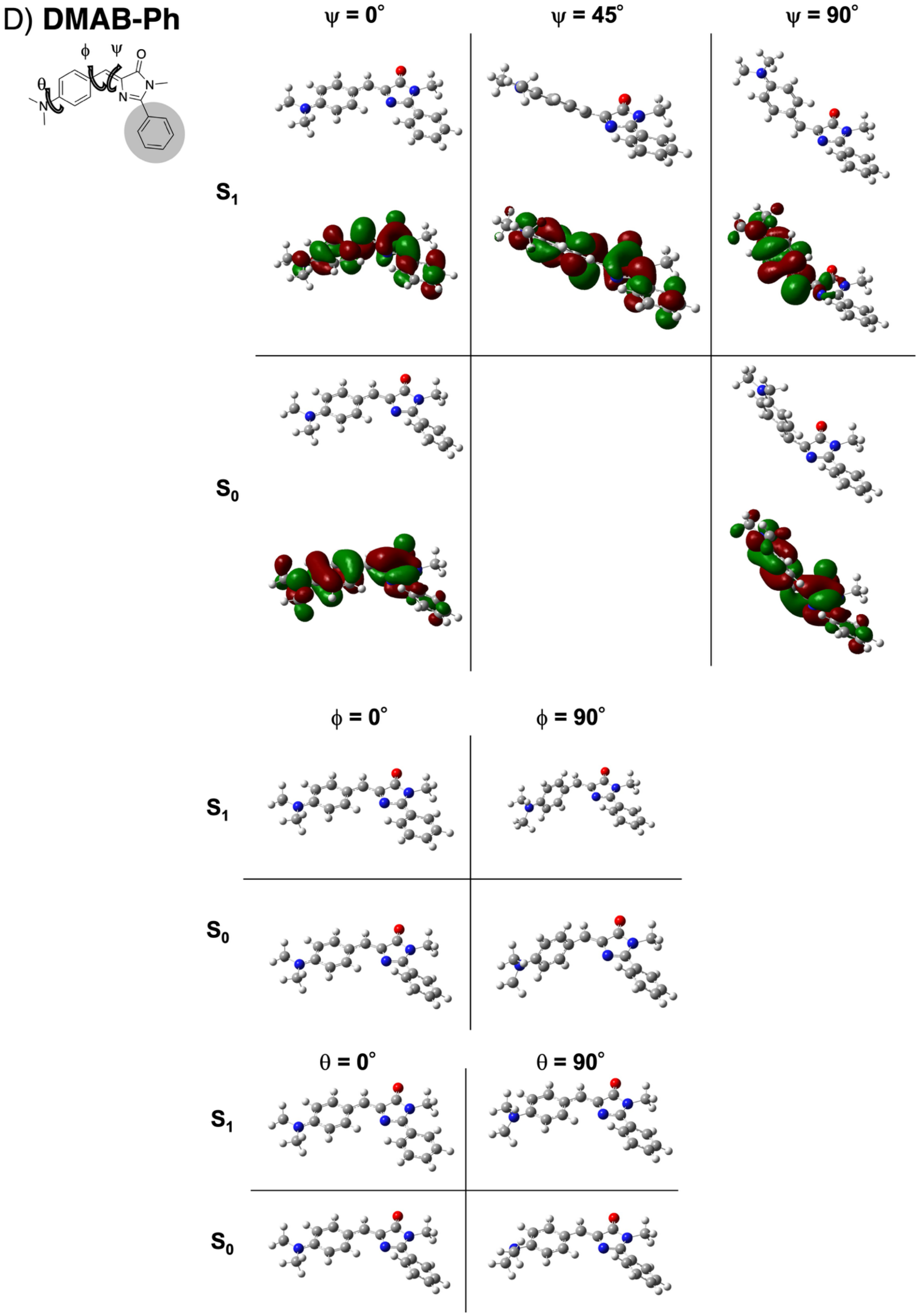
D) DMAB-Ph
θ
ϕ
ψ
O
N
N
ψ = 0°
ψ = 45°
ψ = 90°
S1
S0
ϕ = 0°
ϕ = 90°
S1
S0
θ = 0°
θ = 90°
S1
S0

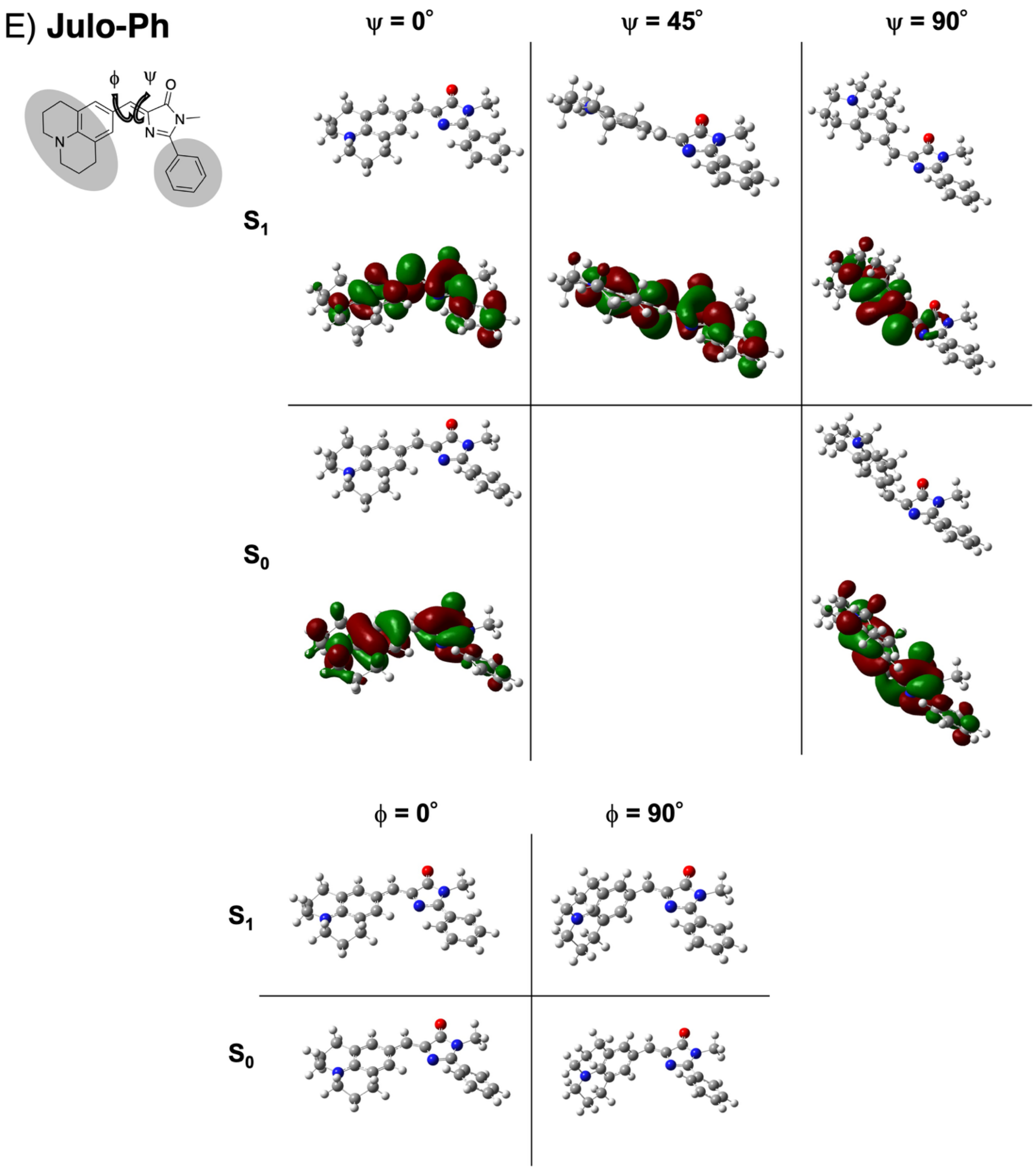

Figure S3. Optimized model structures and their molecular orbitals of A) **DMAB-Me**, B) **PyrroB-Me**, C) **Julo-Me**, D) **DMAB-Ph**, and E) **Julo-Ph** obtained by DFT or TD-DFT B3LYP/6-31+G(d)/IEFPCM (CH_3OH) calculations with each specified dihedral angle.

Table S1: Coordinates of optimized model structures of the GFP dyes A) ~ O) obtained from TD-DFT calculations at the B3LYP/6-31+G(d)/IEFPCM (CH_3OH) level.

A) DMAB-Me (S_1, ψ = 0°)

Atom	Coordinates (Angstroms)		
	X	Y	Z
C	-1.04819	0.624548	-0.000813
C	-0.648449	-0.753927	-0.001067
C	-1.712706	-1.716055	-0.001037
C	-3.040817	-1.351447	-0.00058
C	-3.426416	0.027086	-0.000258
C	-2.379203	0.996691	-0.000524
H	-0.278893	1.38738	-0.000701
H	-1.458339	-2.773475	-0.001435
H	-3.795638	-2.128805	-0.000931
H	-2.619676	2.053561	0.000079
C	0.699519	-1.203596	-0.001007
H	0.854781	-2.279989	-0.001342
C	1.893298	-0.439497	-0.000449
C	3.222121	-1.059479	-0.000466
C	3.346971	1.186143	0.000622
N	2.032539	0.926538	0.00023
O	3.573059	-2.258728	-0.000922
N	4.099984	0.040201	0.000016
C	3.943013	2.54782	0.001556
H	3.142422	3.291117	0.002378
H	4.577138	2.715436	0.884004
H	4.576601	2.716915	-0.880998
C	5.55041	-0.061252	0.001025
H	5.974113	0.417327	-0.887381
H	5.972315	0.404394	0.897217
H	5.806469	-1.122247	-0.006322
N	-4.746758	0.396322	0.000372
C	-5.800082	-0.613243	0.00518
H	-5.733624	-1.253147	0.894945
H	-6.76952	-0.116585	0.0108
H	-5.743222	-1.252288	-0.885995
C	-5.119436	1.806382	-0.003446
H	-6.205512	1.888679	-0.007777
H	-4.735522	2.321289	0.887367
H	-4.728926	2.31778	-0.893277

B) DMAB-Me (S_1, ψ = 45°)

Atom	Coordinates (Angstroms)		
	X	Y	Z
C	0.95139	0.537348	0.040536
C	0.629528	-0.80421	-0.366149
C	1.746096	-1.692868	-0.539958
C	3.046351	-1.288189	-0.351021
C	3.353645	0.055535	0.042085
C	2.253244	0.947636	0.232745
H	0.142781	1.243207	0.195416
H	1.550802	-2.719814	-0.840464
H	3.844044	-2.004641	-0.507883
H	2.433545	1.970569	0.541967
C	-0.682072	-1.264972	-0.576855
H	-0.803115	-2.332933	-0.757307
C	-1.897191	-0.517573	-0.37888
C	-3.07825	-1.03541	0.293152
C	-3.363489	1.097585	-0.372392
N	-2.119972	0.775189	-0.766281
O	-3.338918	-2.152004	0.791812
N	-3.984196	0.054313	0.250091
C	-3.992394	2.430663	-0.568148
H	-3.295293	3.082438	-1.099766
H	-4.921309	2.361564	-1.151185
H	-4.251856	2.903333	0.390021
C	-5.321561	0.033546	0.821298
H	-5.410389	0.759565	1.635687
H	-6.07166	0.25562	0.056155
H	-5.495417	-0.969863	1.213985
N	4.64424	0.462998	0.227751
C	5.751343	-0.475625	0.064458
H	5.790313	-0.872188	-0.958329
H	6.688487	0.041818	0.266533
H	5.665573	-1.319097	0.761804
C	4.936779	1.838489	0.620622
H	6.016431	1.968077	0.68587
H	4.547669	2.552376	-0.116428
H	4.499729	2.076787	1.599549

C) DMAB-Me (S_1, ψ = 90°)

Atom	Coordinates (Angstroms)		
	X	Y	Z
C	0.855716	0.430655	-0.003433
C	0.612756	-0.945348	-0.322828
C	1.783536	-1.757956	-0.485314
C	3.05847	-1.255461	-0.34058
C	3.283815	0.117132	-0.019129
C	2.13135	0.938922	0.143345
H	0.011157	1.100723	0.127001
H	1.656415	-2.809929	-0.729782
H	3.898602	-1.926517	-0.476139
H	2.243826	1.989044	0.386521
C	-0.66906	-1.490928	-0.468519
H	-0.758914	-2.547165	-0.715087
C	-1.919	-0.723135	-0.336619
C	-2.693501	-0.558199	0.883148
C	-3.639	0.485438	-0.857849
N	-2.524237	-0.082233	-1.360523
O	-2.513545	-0.977459	2.038335
N	-3.790062	0.237049	0.471217
C	-4.602394	1.291134	-1.653462
H	-4.267562	1.335898	-2.691448
H	-5.607333	0.852085	-1.622958
H	-4.682731	2.313194	-1.262512
C	-4.86757	0.68119	1.343625
H	-4.914675	1.773381	1.374387
H	-5.828819	0.284465	1.004644
H	-4.655102	0.301882	2.344526
N	4.552874	0.62096	0.124738
C	5.713943	-0.247037	-0.038901
H	5.750087	-0.684219	-1.045021
H	6.620535	0.339926	0.107939
H	5.71327	-1.064593	0.694053
C	4.753075	2.024351	0.468462
H	5.822152	2.225142	0.537297
H	4.329308	2.689515	-0.295101
H	4.295306	2.271877	1.435313

D) PyrroB-Me (S_1, ψ = 0°)

Atom	Coordinates (Angstroms)		
	X	Y	Z
C	-0.375488	0.508337	0.007412
C	0.083849	-0.851436	-0.02719
C	-0.935316	-1.861207	-0.054746
C	-2.278365	-1.556819	-0.046735
C	-2.722064	-0.196542	-0.01083
C	-1.72181	0.820701	0.014902
H	0.36008	1.303495	0.032159
H	-0.632269	-2.905249	-0.086806
H	-3.003785	-2.362203	-0.079588
H	-2.016821	1.863883	0.052992
C	1.451356	-1.241463	-0.034693
H	1.653221	-2.309769	-0.05933
C	2.610095	-0.427289	-0.011864
C	3.964281	-0.990212	-0.018201
C	3.992849	1.258261	0.031192
N	2.691449	0.944064	0.017958
O	4.365661	-2.17353	-0.042989
N	4.794191	0.144665	0.010692
C	4.532609	2.643129	0.06311
H	3.702301	3.353005	0.076309
H	5.156772	2.818196	0.951206
H	5.161404	2.856566	-0.813348
C	6.247478	0.103997	0.017084
H	6.654903	0.607339	-0.865363
H	6.645847	0.579148	0.919008
H	6.547428	-0.945504	0.002066
N	-4.050717	0.110831	-0.001794
C	-5.126202	-0.88757	0.080332
H	-4.915841	-1.616494	0.870802
H	-5.206833	-1.43538	-0.871488
C	-4.575973	1.481322	-0.066951
H	-4.387284	2.003357	0.884318
H	-4.079091	2.046282	-0.863701
C	-6.077368	1.290154	-0.319371
H	-6.667349	2.119391	0.081222
H	-6.26963	1.225193	-1.396867
C	-6.383316	-0.053387	0.360742
H	-6.505829	0.085162	1.441439
H	-7.286679	-0.534949	-0.024277

E) PyrroB-Me (S_1, ψ = 45°)

Atom	Coordinates (Angstroms)		
	X	Y	Z
C	0.290309	0.399787	-0.021192
C	-0.106427	-0.943033	-0.355102
C	0.960096	-1.896206	-0.509662
C	2.282502	-1.549345	-0.371123
C	2.66253	-0.204826	-0.050287
C	1.614571	0.751934	0.122997
H	-0.479232	1.152492	0.1104
H	0.704858	-2.925887	-0.749883
H	3.045211	-2.309743	-0.497812
H	1.861278	1.78001	0.364974
C	-1.443079	-1.345183	-0.515521
H	-1.621847	-2.412699	-0.643153
C	-2.614502	-0.523634	-0.339809
C	-3.807175	-0.936266	0.380979
C	-3.998061	1.160548	-0.42021
N	-2.779432	0.751048	-0.809151
O	-4.114747	-2.004134	0.955698
N	-4.658565	0.192626	0.278945
C	-4.565008	2.507918	-0.693095
H	-3.846751	3.088824	-1.276271
H	-5.506613	2.447631	-1.256468
H	-4.784256	3.053165	0.236229
C	-5.987053	0.27665	0.864414
H	-6.029838	1.061423	1.626305
H	-6.736399	0.480892	0.093483
H	-6.203376	-0.6871	1.328657
N	3.968505	0.142835	0.085678
C	5.096004	-0.767733	-0.169852
H	4.957833	-1.295179	-1.120015
H	5.164546	-1.521	0.629564
C	4.423479	1.478154	0.503328
H	4.249516	2.20656	-0.3032
H	3.8703	1.815137	1.386943
C	5.920537	1.287257	0.78128
H	6.48651	2.209405	0.621109
H	6.070173	0.972912	1.821046
C	6.320126	0.157595	-0.180568
H	6.478882	0.55506	-1.190058
H	7.230525	-0.366094	0.124809

F) PyrroB-Me (S_1, ψ = 90°)

Atom	Coordinates (Angstroms)		
	X	Y	Z
C	-0.204585	-0.258994	-0.102125
C	0.130479	1.133717	-0.172297
C	-0.98287	2.039224	-0.192497
C	-2.289316	1.604977	-0.141001
C	-2.604305	0.214936	-0.065815
C	-1.512223	-0.699362	-0.050001
H	0.59362	-0.99543	-0.095391
H	-0.782854	3.106968	-0.243659
H	-3.088009	2.338821	-0.148499
H	-1.704178	-1.766059	-0.007211
C	1.446647	1.611161	-0.214585
H	1.60805	2.686093	-0.269526
C	2.642577	0.752268	-0.210481
C	3.38273	0.328008	0.967754
C	4.291688	-0.459319	-0.921013
N	3.224817	0.258689	-1.32552
O	3.208295	0.55213	2.176744
N	4.433772	-0.454412	0.432096
C	5.216171	-1.177799	-1.837336
H	4.894491	-1.026232	-2.869346
H	6.245258	-0.813247	-1.728942
H	5.228844	-2.254027	-1.624138
C	5.464124	-1.111925	1.222913
H	5.439633	-2.194095	1.066892
H	6.455169	-0.728769	0.963603
H	5.258238	-0.894887	2.272399
N	-3.898175	-0.218887	-0.013231
C	-4.282874	-1.624284	0.176271
C	-5.065066	0.667257	-0.123225
H	-3.716086	-2.074719	0.998836
H	-4.07733	-2.207335	-0.733984
C	-5.787676	-1.554102	0.472992
H	-5.173225	1.278814	0.785164
H	-4.95801	1.347223	-0.975733
C	-6.243632	-0.301792	-0.293091
H	-5.949857	-1.419572	1.549308
H	-6.311208	-2.463311	0.163225
H	-7.179815	0.118368	0.086016
H	-6.383195	-0.537216	-1.355154

G) Julo-Me (S_1, $\psi = 0°$)

Atom	Coordinates (Angstroms)		
	X	Y	Z
C	-0.309946	0.591633	-0.074297
C	0.063948	-0.788356	-0.054894
C	-1.006462	-1.735931	-0.061362
C	-2.337509	-1.369772	-0.081902
C	-2.684174	0.026644	-0.083477
C	-1.633425	1.001343	-0.095015
H	0.475477	1.33899	-0.072647
H	-0.759907	-2.796192	-0.050376
C	1.411908	-1.259778	-0.025079
H	1.548724	-2.338501	-0.012929
C	2.611896	-0.517365	-0.00954
C	3.932355	-1.159161	0.021371
C	4.090062	1.085224	0.001913
N	2.77594	0.850801	-0.020224
N	4.826106	-0.076188	0.027437
C	4.712895	2.435703	-0.000473
H	3.926377	3.193685	-0.023923
H	5.33217	2.602373	0.8926
H	5.366474	2.583308	-0.872172
C	6.274151	-0.200296	0.057478
H	6.723449	0.254349	-0.831128
H	6.687968	0.274387	0.952813
H	6.513174	-1.265207	0.07424
N	-4.005467	0.416066	-0.038
C	-5.087985	-0.559599	-0.167335
H	-5.980052	-0.125911	0.294511
H	-5.316886	-0.712245	-1.237551
C	-4.381268	1.822523	-0.182379
H	-4.489367	2.063195	-1.255276
H	-5.365524	1.950587	0.277937
C	-3.352383	2.747753	0.459995
C	-1.97173	2.476328	-0.139564
H	-3.337473	2.577877	1.544328
H	-3.653332	3.787615	0.293057
H	-1.198441	3.049467	0.384177
H	-1.959017	2.821682	-1.184733
C	-4.727238	-1.890808	0.484439
C	-3.425794	-2.420472	-0.119349
H	-5.547072	-2.599824	0.327008
H	-4.615831	-1.748387	1.567062
H	-3.609934	-2.71572	-1.163744
H	-3.089239	-3.323008	0.402843
O	4.262052	-2.365119	0.040778

H) Julo-Me (S_1, ψ = 45°)

Atom	Coordinates (Angstroms)		
	X	Y	Z
C	0.231984	0.491703	0.010551
C	-0.067389	-0.887588	-0.245682
C	1.054236	-1.780145	-0.290826
C	2.355306	-1.360218	-0.126132
C	2.626102	0.037364	0.096029
C	1.522069	0.951624	0.184135
H	-0.590527	1.197542	0.066549
H	0.865299	-2.837262	-0.47033
C	-1.373369	-1.385243	-0.431776
H	-1.478063	-2.469156	-0.472816
C	-2.601228	-0.644305	-0.337791
C	-3.787419	-1.113765	0.363296
C	-4.087154	0.943097	-0.503653
N	-2.834193	0.608009	-0.843227
N	-4.705686	-0.048969	0.204777
C	-4.738132	2.238201	-0.836287
H	-4.042581	2.852729	-1.41259
H	-5.652543	2.094979	-1.428834
H	-5.028161	2.792324	0.068089
C	-6.055579	-0.038644	0.74515
H	-6.178867	0.76788	1.474892
H	-6.792265	0.083575	-0.054981
H	-6.219017	-0.998036	1.239313
N	3.919398	0.488126	0.191992
C	5.048575	-0.443207	0.246856
H	5.935905	0.095281	-0.099533
H	5.233927	-0.736434	1.294956
C	4.215987	1.877622	0.54869
H	4.272921	1.970459	1.647188
H	5.208429	2.114434	0.152885
C	3.167898	2.837557	-0.004703
C	1.779659	2.415109	0.478149
H	3.208364	2.829004	-1.101634
H	3.404184	3.854728	0.32611
H	1.000023	3.02925	0.013703
H	1.708494	2.589257	1.562506
C	4.798012	-1.681886	-0.607138
C	3.497691	-2.353706	-0.164411
H	5.64636	-2.366834	-0.50255
H	4.736261	-1.390288	-1.663582
H	3.640689	-2.783459	0.838676
H	3.242154	-3.188804	-0.826175
O	-4.037638	-2.181594	0.965829

I) **Julo-Me (S_1, ψ = 90°)**

Atom	Coordinates (Angstroms)		
	X	Y	Z
C	0.153237	0.361152	-0.019819
C	-0.065028	-1.048388	-0.142157
C	1.115737	-1.859735	-0.1606
C	2.38882	-1.338461	-0.065139
C	2.571641	0.079648	0.038675
C	1.414033	0.919115	0.07813
H	-0.70632	1.026454	-0.000854
H	0.999005	-2.938576	-0.248172
C	-1.335998	-1.628094	-0.233093
H	-1.407523	-2.710573	-0.323402
C	-2.599032	-0.871789	-0.232679
C	-3.399771	-0.551114	0.939097
C	-4.332023	0.215695	-0.94659
N	-3.198572	-0.394338	-1.346203
N	-4.502692	0.155151	0.401976
C	-5.602466	0.697376	1.186615
H	-5.661929	1.78331	1.070634
H	-6.552282	0.246281	0.885646
H	-5.406424	0.457582	2.23303
N	3.842099	0.626441	0.057984
C	5.020075	-0.228216	0.209643
H	5.881718	0.327312	-0.174707
H	5.211613	-0.427618	1.27806
C	4.036482	2.044586	0.360671
H	4.066595	2.200755	1.452777
H	5.015434	2.334782	-0.03438
C	2.933831	2.897641	-0.259407
C	1.570555	2.41915	0.243297
H	2.985612	2.817316	-1.353112
H	3.097583	3.948975	0.002083
H	0.758577	2.937542	-0.279635
H	1.470884	2.681649	1.307213
C	4.850476	-1.543156	-0.545418
C	3.593378	-2.260503	-0.050409
H	5.738991	-2.166011	-0.393497
H	4.771788	-1.336217	-1.620734
H	3.76789	-2.616835	0.975908
H	3.387892	-3.150828	-0.655769
O	-3.233916	-0.798558	2.144884
C	-5.296191	0.879577	-1.863329
H	-4.937828	0.793479	-2.890835
H	-6.290039	0.419839	-1.794521
H	-5.412282	1.942167	-1.616019

J) DMAB-Ph (S_1, $\psi = 0°$)

Atom	Coordinates (Angstroms)		
	X	Y	Z
C	-1.886408	0.299391	-0.004976
C	-1.883532	-1.133869	0.01109
C	-3.168414	-1.767824	0.028754
C	-4.34497	-1.053256	0.030418
C	-4.336098	0.378333	0.014166
C	-3.061678	1.023402	-0.003638
H	-0.936034	0.819616	-0.017539
H	-3.213237	-2.854341	0.041311
H	-5.284185	-1.593188	0.044027
H	-3.002637	2.105362	-0.015618
C	-0.711399	-1.934097	0.009247
H	-0.85168	-3.01237	0.020921
C	0.63797	-1.51824	-0.004478
C	1.750808	-2.489353	-0.012178
C	2.500089	-0.359228	-0.010613
N	1.142287	-0.263241	-0.012627
O	1.735359	-3.730151	-0.030284
N	2.897995	-1.693136	-0.014464
C	4.224324	-2.286119	-0.123691
H	4.783933	-1.832591	-0.945091
H	4.786128	-2.184062	0.809512
H	4.080967	-3.348323	-0.330111
N	-5.498895	1.095392	0.016568
C	-6.791804	0.415991	0.038789
H	-6.899046	-0.203934	0.938013
H	-7.585563	1.161856	0.039295
H	-6.91911	-0.224158	-0.843514
C	-5.469125	2.55537	-0.001621
H	-6.490957	2.932254	0.002829
H	-4.949225	2.95243	0.879726
H	-4.964988	2.930477	-0.901535
C	3.336662	0.811417	0.003887
C	2.710697	2.078376	-0.202253
C	4.742053	0.810726	0.233816
C	3.446763	3.255317	-0.195624
H	1.64055	2.104813	-0.373082
C	5.466185	1.99913	0.240498
H	5.27174	-0.110875	0.432496
C	4.833944	3.231978	0.022491
H	2.939257	4.202374	-0.363068
H	6.537028	1.963686	0.425143
H	5.408073	4.154412	0.027721

K) DMAB-Ph (S_1, ψ = 45°)

Atom	Coordinates (Angstroms)		
	X	Y	Z
C	1.843662	0.28369	0.006638
C	1.882035	-1.067836	-0.477019
C	3.188823	-1.628393	-0.675429
C	4.34093	-0.919987	-0.425473
C	4.288992	0.428242	0.055575
C	2.994028	0.997686	0.264221
H	0.878813	0.750828	0.170332
H	3.266637	-2.650719	-1.038309
H	5.296833	-1.399866	-0.598463
H	2.902215	2.01295	0.631821
C	0.736677	-1.845532	-0.744916
H	0.910627	-2.894155	-0.981568
C	-0.625988	-1.507097	-0.46434
C	-1.576858	-2.447991	0.138507
C	-2.476908	-0.391382	-0.105818
N	-1.203838	-0.292415	-0.573467
O	-1.455906	-3.646427	0.448632
N	-2.736699	-1.679609	0.329995
C	-3.888683	-2.219188	1.040906
H	-4.212377	-1.532722	1.82652
H	-4.720564	-2.421152	0.359336
H	-3.572376	-3.161864	1.491311
N	5.428154	1.137453	0.303735
C	6.741352	0.534856	0.087359
H	6.871786	0.233028	-0.959609
H	7.511731	1.265148	0.331859
H	6.885864	-0.346836	0.72502
C	5.354179	2.507669	0.804411
H	6.364423	2.894574	0.931673
H	4.820425	3.15919	0.100783
H	4.842725	2.549646	1.77479
C	-3.374213	0.739322	-0.10635
C	-2.818026	2.034916	-0.306989
C	-4.783356	0.647957	0.0497
C	-3.623164	3.16653	-0.329604
H	-1.745275	2.125345	-0.436208
C	-5.580095	1.790038	0.021143
H	-5.266673	-0.313685	0.161951
C	-5.012659	3.05795	-0.162421
H	-3.168073	4.142929	-0.476595
H	-6.656329	1.687791	0.135027
H	-5.640852	3.944286	-0.181159

L) DMAB-Ph (S_1, ψ = 90°)

Atom	Coordinates (Angstroms)		
	X	Y	Z
C	-2.012336	-0.056665	0.34244
C	-1.923171	0.642966	-0.904987
C	-3.152139	0.765892	-1.6337
C	-4.342601	0.250374	-1.16871
C	-4.415447	-0.442461	0.077214
C	-3.203028	-0.574315	0.813372
H	-1.115613	-0.1887	0.940694
H	-3.140616	1.287384	-2.587889
H	-5.234215	0.380391	-1.770893
H	-3.200054	-1.09173	1.765593
C	-0.731628	1.183243	-1.406865
H	-0.757106	1.694204	-2.367516
C	0.572027	1.090707	-0.730253
C	1.121673	2.068416	0.198411
C	2.53415	0.360187	-0.145671
N	1.442636	0.086393	-0.905299
O	0.655992	3.126364	0.650073
N	2.387696	1.536456	0.538749
C	3.237999	2.150713	1.552039
H	3.674388	1.384527	2.195232
H	4.032731	2.748486	1.096271
H	2.604817	2.80975	2.148786
N	-5.599558	-0.958764	0.542837
C	-6.82754	-0.798975	-0.228369
H	-6.744641	-1.267601	-1.217438
H	-7.648024	-1.278191	0.305655
H	-7.082067	0.260033	-0.36718
C	-5.647153	-1.64845	1.827272
H	-6.667867	-1.981401	2.015159
H	-4.993754	-2.530503	1.834214
H	-5.345491	-0.988066	2.651014
C	3.68845	-0.535214	-0.108097
C	3.4795	-1.901894	-0.391825
C	4.998867	-0.092312	0.161614
C	4.545454	-2.79836	-0.384744
H	2.474052	-2.246211	-0.608531
C	6.063558	-0.994289	0.159515
H	5.200854	0.956941	0.34061
C	5.841919	-2.348999	-0.10686
H	4.365678	-3.849003	-0.595132
H	7.069004	-0.634685	0.359279
H	6.673254	-3.048492	-0.103299

M) Julo-Ph (S_1, $\psi = 0°$)

Atom	Coordinates (Angstroms)		
	X	Y	Z
C	-1.161872	0.189027	-0.064261
C	-1.156549	-1.239251	-0.042311
C	-2.432507	-1.881454	-0.040428
C	-3.624137	-1.187374	-0.061096
C	-3.600694	0.251797	-0.069558
C	-2.333046	0.925104	-0.084216
H	-0.209724	0.707993	-0.065773
H	-2.465118	-2.969418	-0.024951
C	0.023387	-2.038046	-0.022602
H	-0.115844	-3.11632	-0.004009
C	1.366679	-1.620235	-0.01996
C	2.482416	-2.590462	0.001882
C	3.225708	-0.457742	-0.008007
N	1.872589	-0.361332	-0.032859
N	3.626867	-1.794028	0.010456
C	4.955302	-2.38456	-0.08378
H	5.526499	-1.923483	-0.892878
H	5.50425	-2.289951	0.857979
H	4.816998	-3.445409	-0.300922
N	-4.772205	0.965364	-0.039433
C	-6.074399	0.301897	-0.145698
H	-6.815315	0.955226	0.324599
H	-6.350233	0.209104	-1.210257
C	-4.776364	2.423353	-0.184495
H	-4.8191	2.682264	-1.256461
H	-5.695589	2.79722	0.275836
C	-3.547411	3.055219	0.461011
C	-2.280939	2.437422	-0.132897
H	-3.580183	2.889409	1.545519
H	-3.570708	4.137063	0.291752
H	-1.388187	2.793568	0.393225
H	-2.176584	2.765638	-1.178284
C	-6.063003	-1.072313	0.515441
C	-4.946361	-1.922841	-0.09108
H	-7.038917	-1.547343	0.368932
H	-5.909967	-0.956398	1.596066
H	-5.205344	-2.164408	-1.133186
H	-4.85135	-2.879421	0.434624
O	2.468483	-3.831915	-0.004291
C	4.067229	0.710851	0.006775
C	5.46359	0.710226	0.284713
C	3.454754	1.975232	-0.247522
C	6.192843	1.895899	0.290716
H	5.981299	-0.208863	0.523512
C	4.195402	3.14955	-0.240742
H	2.391198	2.001733	-0.45617
C	5.574443	3.12634	0.024882
H	7.256511	1.860073	0.513403
H	3.697857	4.094572	-0.446006
H	6.152113	4.046592	0.030028

N) Julo-Ph (S_1, $\psi = 45°$)

Atom	Coordinates (Angstroms)		
	X	Y	Z
C	1.129323	0.162047	-0.021329
C	1.159485	-1.219884	-0.395331
C	2.455807	-1.818304	-0.506399
C	3.624728	-1.128072	-0.272451
C	3.563262	0.264693	0.088529
C	2.27637	0.889335	0.225801
H	0.164997	0.651302	0.071058
H	2.517443	-2.868709	-0.78583
C	0.006024	-2.000789	-0.638403
H	0.17365	-3.066909	-0.783662
C	-1.354999	-1.637954	-0.40164
C	-2.320111	-2.542421	0.237101
C	-3.199331	-0.492392	-0.11274
N	-1.923528	-0.422874	-0.570283
N	-3.473985	-1.759022	0.381569
C	-4.634711	-2.252752	1.111039
H	-4.963255	-1.520521	1.852027
H	-5.461489	-2.491248	0.434916
H	-4.327015	-3.168584	1.619222
N	4.714221	0.986366	0.272094
C	6.029214	0.339309	0.267639
H	6.769274	1.100244	0.002435
H	6.269933	-0.008747	1.286848
C	4.677482	2.365938	0.765378
H	4.693165	2.36079	1.868733
H	5.595224	2.859287	0.431314
C	3.446397	3.111069	0.260286
C	2.184627	2.34038	0.650383
H	3.50657	3.216694	-0.830602
H	3.436942	4.118884	0.689247
H	1.293214	2.799624	0.208739
H	2.053985	2.394374	1.741784
C	6.077889	-0.828955	-0.71141
C	4.965883	-1.823595	-0.376434
H	7.061116	-1.307572	-0.649296
H	5.957212	-0.4523	-1.735262
H	5.198829	-2.311692	0.58204
H	4.913729	-2.621034	-1.126075
O	-2.208561	-3.724968	0.607982
C	-4.093021	0.639734	-0.175536
C	-5.504942	0.559	-0.038989
C	-3.530032	1.924435	-0.422079
C	-6.297618	1.700885	-0.129001
H	-5.993012	-0.39609	0.105448
C	-4.331171	3.056086	-0.50628
H	-2.454905	2.006484	-0.536908
C	-5.723721	2.958707	-0.357165
H	-7.375935	1.605928	-0.028496
H	-3.870439	4.024206	-0.687247
H	-6.348718	3.845037	-0.423751

O) Julo-Ph (S_1, ψ = 90°)

Atom	Coordinates (Angstroms)		
	X	Y	Z
C	1.273284	-0.0057	0.360514
C	1.182825	-1.071526	-0.59049
C	2.416191	-1.458682	-1.207723
C	3.623441	-0.857729	-0.919172
C	3.674939	0.21916	0.0256
C	2.465385	0.618816	0.675981
H	0.36842	0.330831	0.860433
H	2.397042	-2.268267	-1.935303
C	-0.016855	-1.716546	-0.916034
H	0.005955	-2.514421	-1.656227
C	-1.327088	-1.373573	-0.341475
C	-1.925667	-1.980523	0.840382
C	-3.277251	-0.446503	-0.08164
N	-2.164251	-0.462175	-0.858565
N	-3.179947	-1.33647	0.953752
C	-4.065537	-1.561898	2.090383
H	-4.489899	-0.616553	2.433101
H	-4.870019	-2.258506	1.837083
H	-3.462748	-2.000601	2.887585
N	4.862284	0.885676	0.270148
C	6.129551	0.365572	-0.24411
H	6.839544	1.198516	-0.273744
H	6.541453	-0.388858	0.448001
C	4.962706	1.847096	1.368371
H	5.184459	1.323902	2.314368
H	5.812665	2.503362	1.155054
C	3.679541	2.660656	1.509031
C	2.494212	1.717307	1.721952
H	3.529851	3.260504	0.601764
H	3.780089	3.354991	2.350604
H	1.548122	2.270603	1.706267
H	2.572782	1.266571	2.722659
C	5.955759	-0.23986	-1.6338
C	4.897732	-1.343598	-1.583671
H	6.916502	-0.637524	-1.979201
H	5.651757	0.546817	-2.33669
H	5.301305	-2.197557	-1.019256
H	4.673903	-1.716211	-2.589855
O	-1.503171	-2.84368	1.625616
C	-4.405769	0.435417	-0.372039
C	-5.733785	0.131002	-0.011781
C	-4.152001	1.629363	-1.080424
C	-6.772143	1.003506	-0.340038
H	-5.969383	-0.798159	0.493336
C	-5.191991	2.49965	-1.398437
H	-3.132639	1.86314	-1.36806
C	-6.506384	2.191748	-1.027618
H	-7.791527	0.749003	-0.063888
H	-4.978001	3.419626	-1.935376
H	-7.317442	2.870092	-1.27751

Synthetic scheme of glucosamine probes

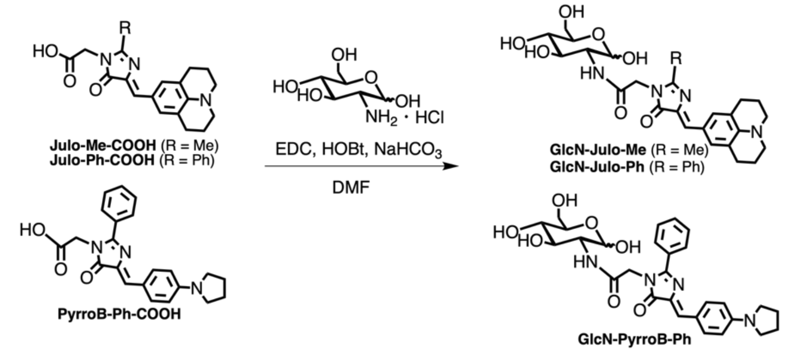

Scheme S2. Synthetic scheme for glucosamine modified probes (**GlcN-Julo-Me**, **GlcN-PyrroB-Ph**, and **GlcN-Julo-Ph**).

Synthetic Scheme of Glucose-peptide probes

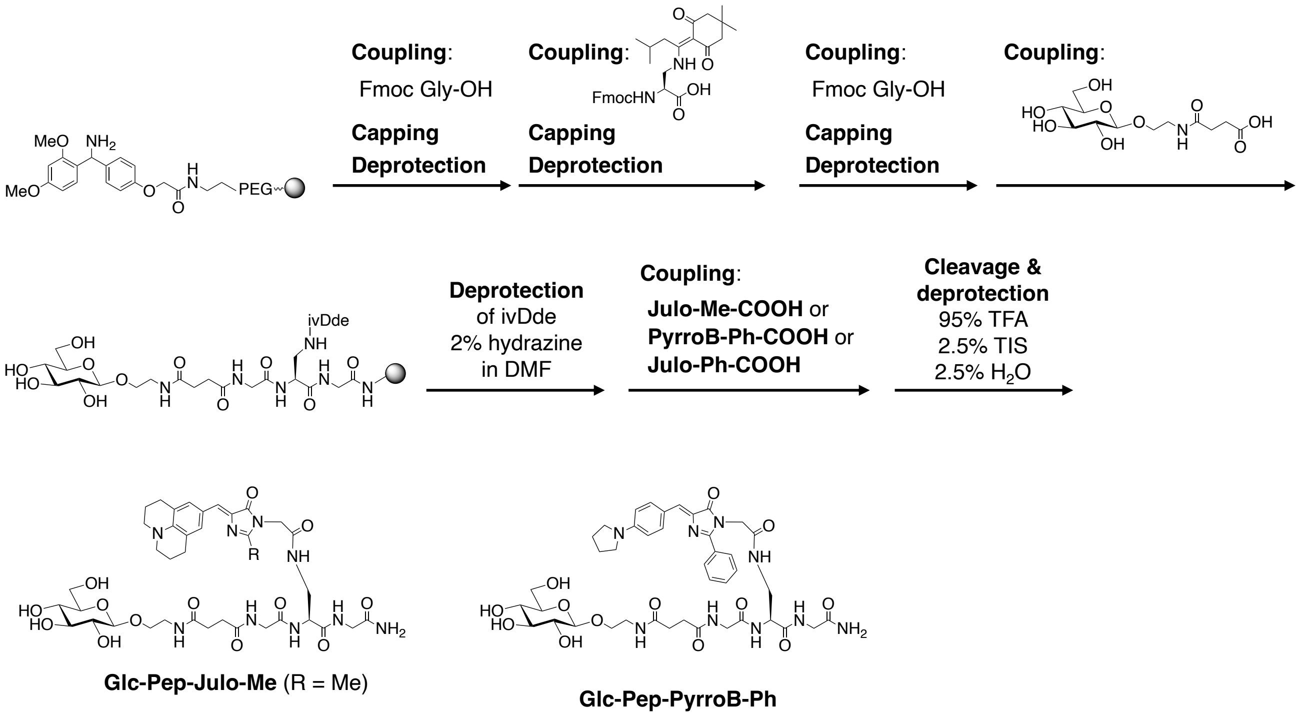

Scheme S3. Synthetic scheme for SPPS for Glucose-peptide probes (**Glc-Pep-Julo-Me**, **Glc-Pep-PyrroB-Ph**, and **Glc-Pep-Julo-Ph**).

Confocal imaging

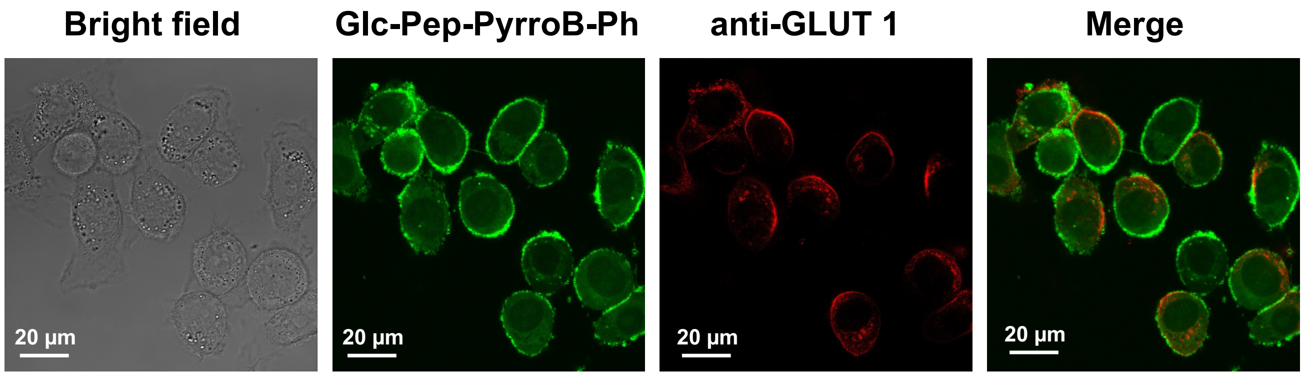

Figure S4. Fluorescence confocal laser scanning microscope (CLSM) images of the immunostaining of the **Glc-Pep-PyrroB-Ph**-treated PC-3 with an anti-GLUT1 antibody. Green channel represents **Glc-Pep-PyrroB-Ph** probe and red channel represents anti-GLUT1 antibody. Anti-GLUT1 antibody-treated cells were further stained with fluorescent labeled second antibody. The gray scale bars correspond to 20 μm.

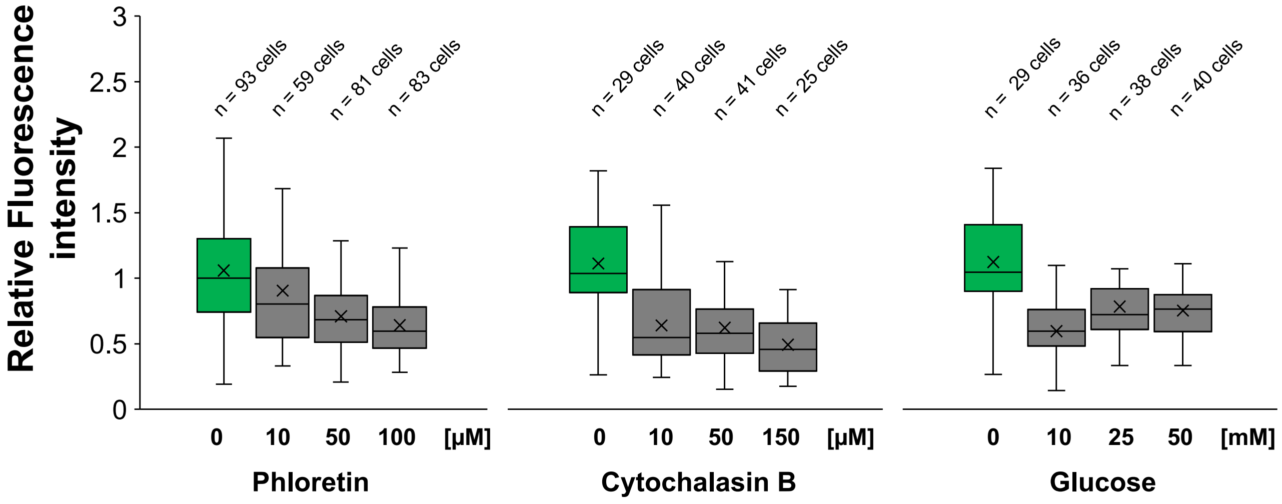

Figure S5. Quantification of relative fluorescence intensity of GLUT inhibitor-treated cells. Fluorescence intensities were quantified for individual cells, with a total of n cells analyzed from three fields of view for each condition. Error bars represent standard deviation.

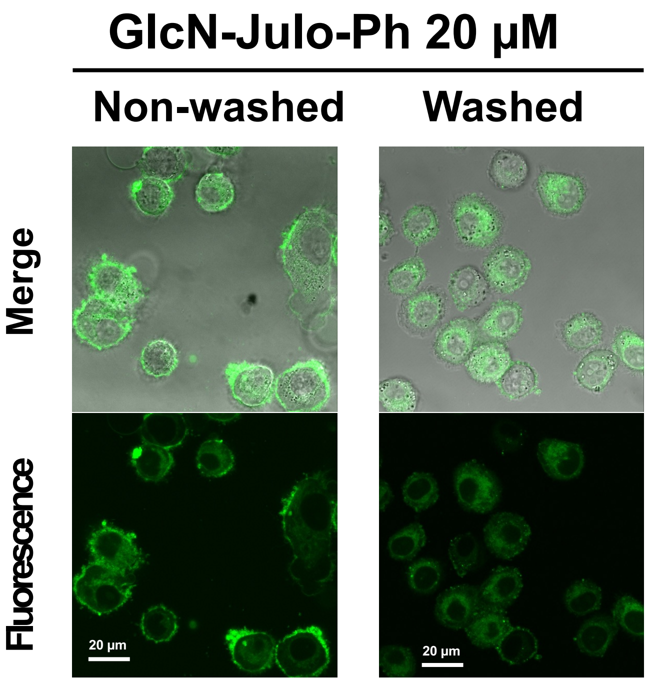

Figure S6. Fluorescence confocal laser scanning microscopy (CLSM) images of PC-3 cells stained with **GlcN-Julo-Ph** after washing. The membrane-associated fluorescence emissions of **GlcN-Julo-Ph** disappeared after washing. The gray scale bars correspond to 20 μm.

Relationship between viscosity and fluorescence quantum yield

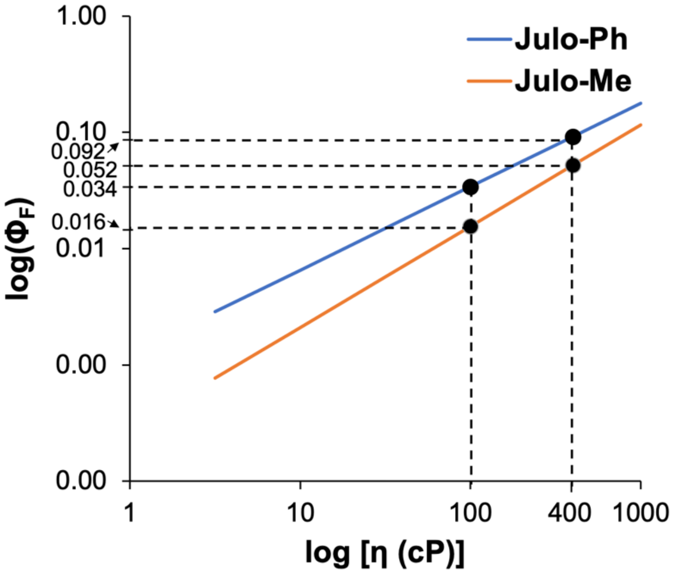

Figure S7. Log–log plot of the relationship between viscosity (cP) and calculated fluorescence quantum yield for **Julo-Ph** and **Julo-Me** in glycerol/methanol mixtures. The straight lines were calculated using χ value (obtained from **Figure** 5) and the fluorescence quantum yield of each dye measured in glycerol (945 cP, **Table** 1).

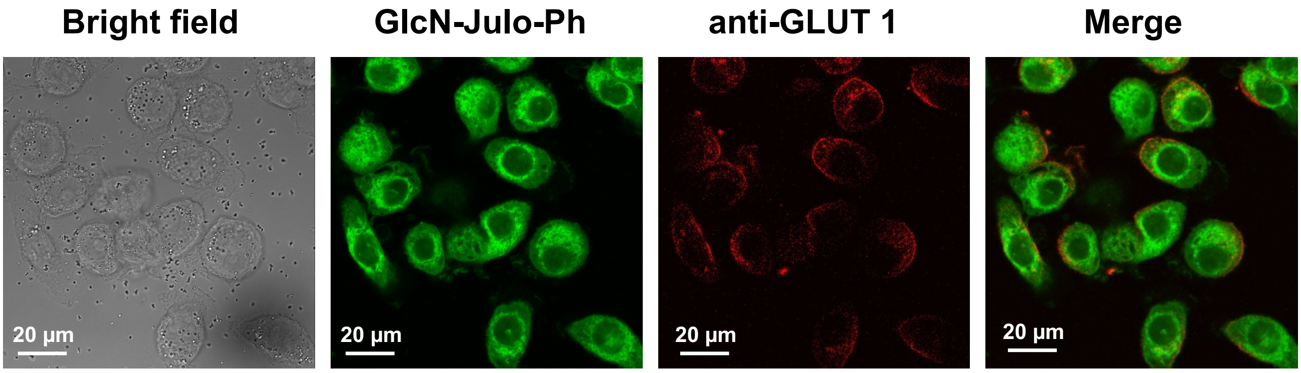

Figure S8. Fluorescence confocal laser scanning microscope (CLSM) images of the immunostaining of the **GlcN-Julo-Ph**-treated PC-3 with an anti-GLUT1 antibody. Green channel represents **GlcN-Julo-Ph** probe and red channel represents anti-GLUT1 antibody. Anti-GLUT1 antibody-treated cells were further stained with fluorescent labeled second antibody. The gray scale bars correspond to 20 μm.

Cytotoxicity

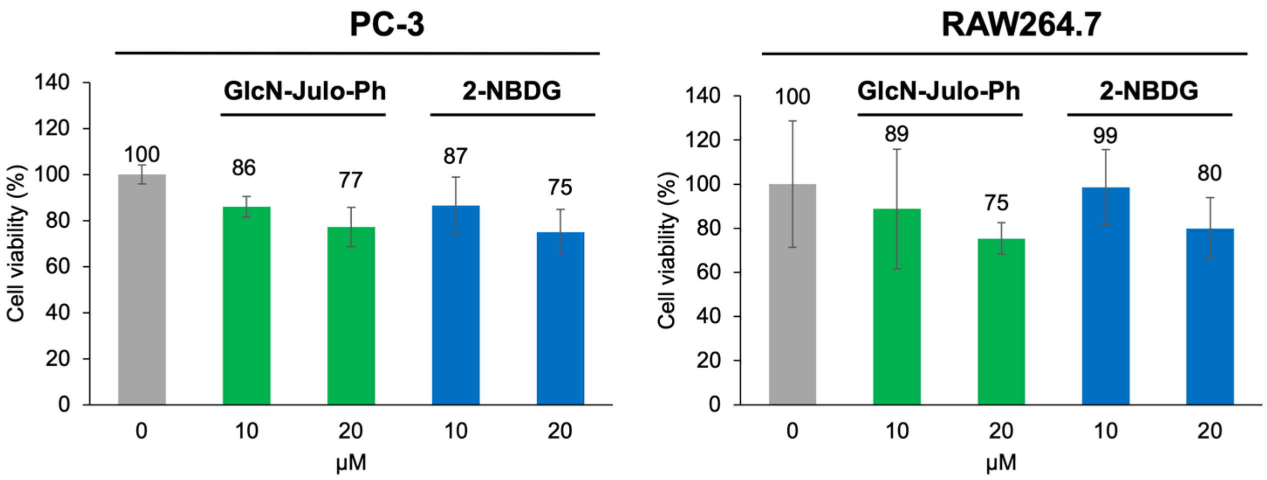

Figure S9. Cytotoxicity of **GlcN-Julo-Ph** and **2-NBDG** toward PC-3 and RAW264.7 cells assessed by the MTT assay (n = 3 ~ 4).

^{1}H NMR, ^{13}C NMR, and ESI-TOF-MS spectra of compounds

Methyl 2-(1-ethoxyethylidene)aminoethanoate

1H-NMR

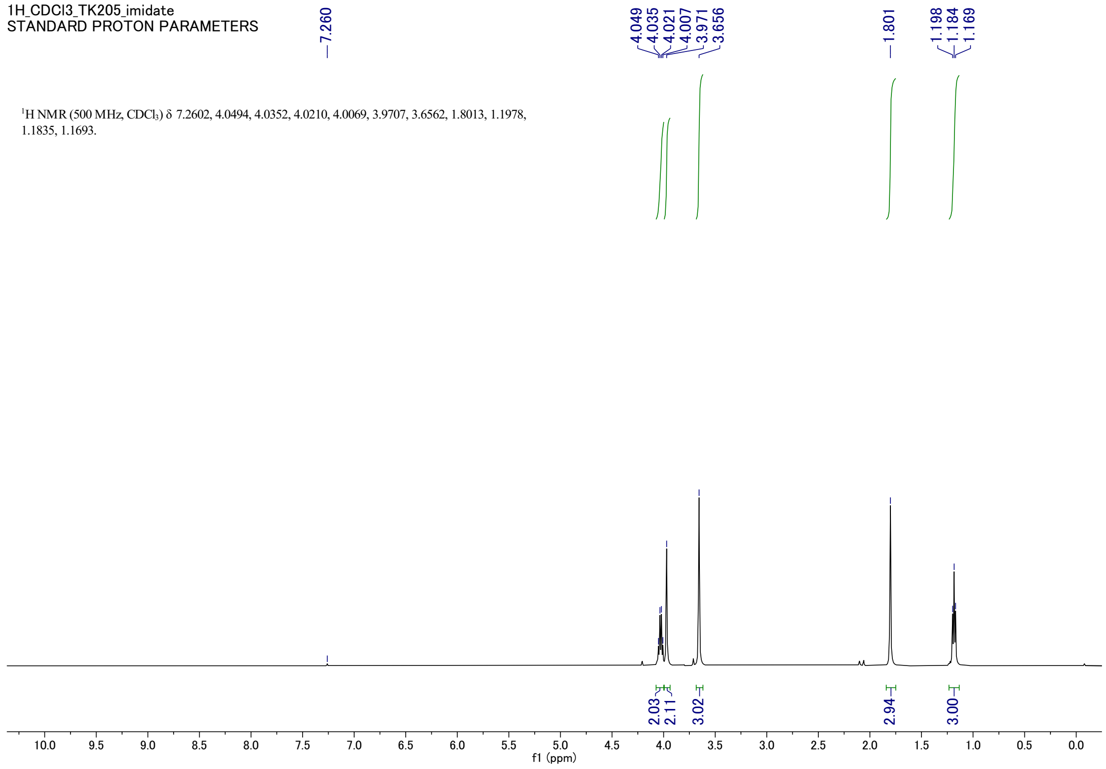

DMAB-Me

1H-NMR

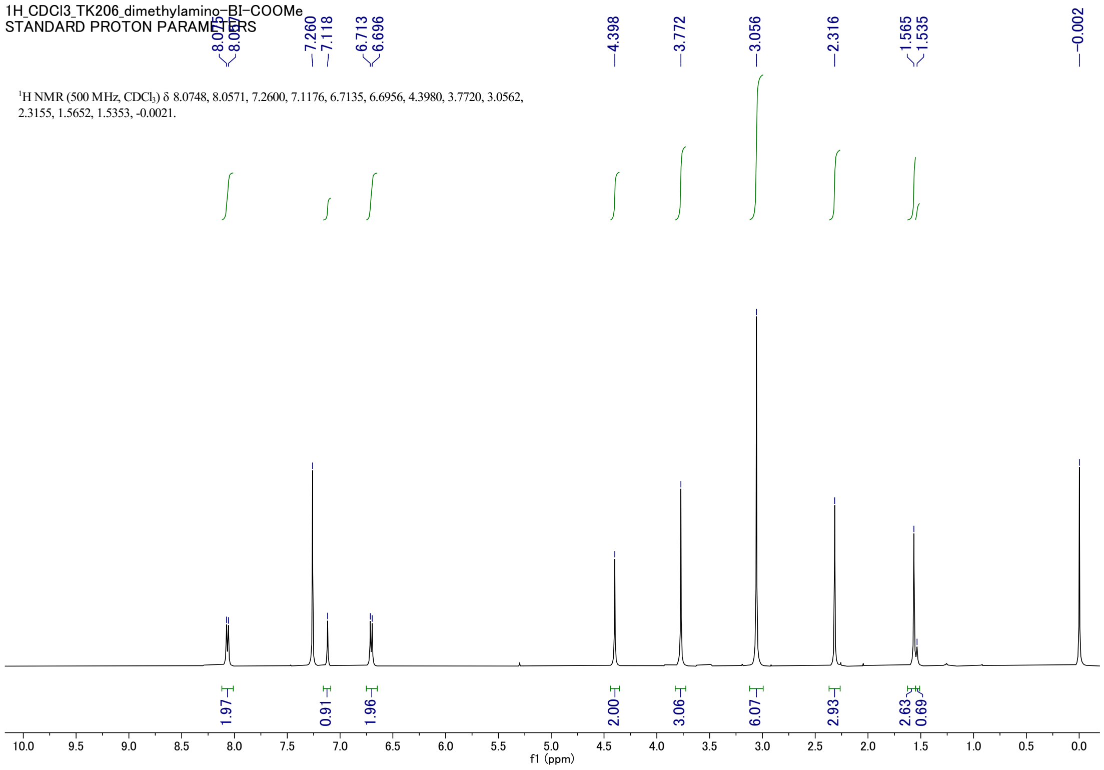

AzeB-Me

1H-NMR

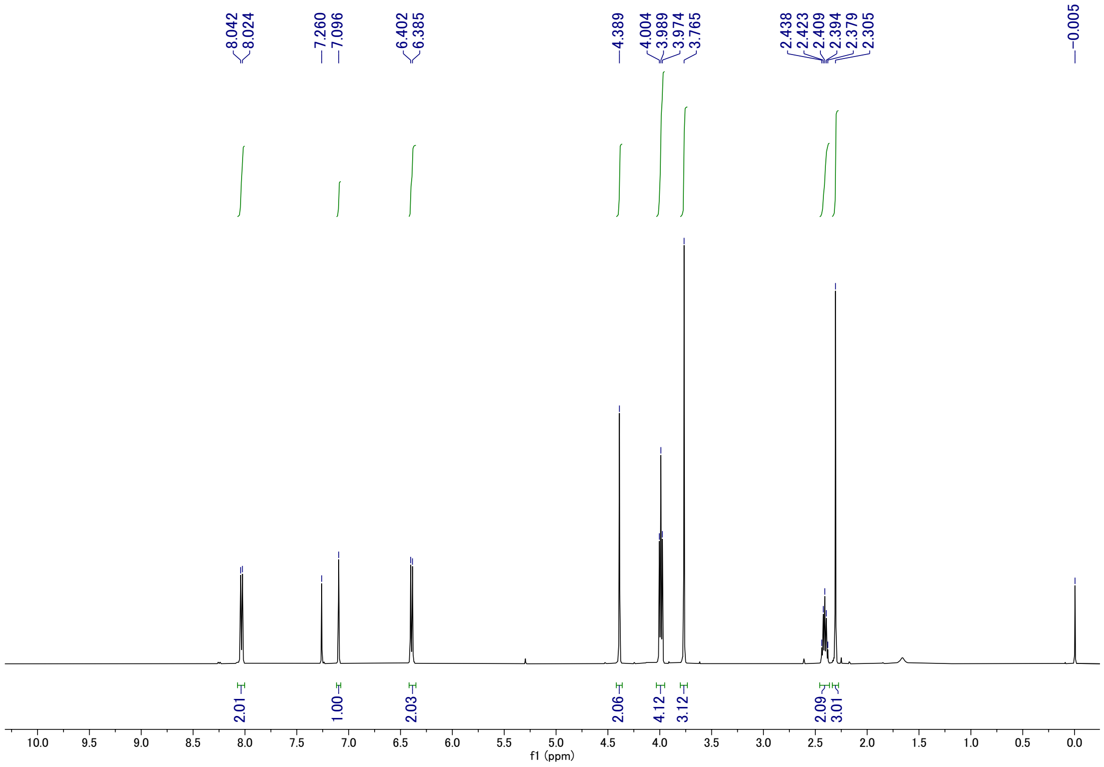

13C-NMR

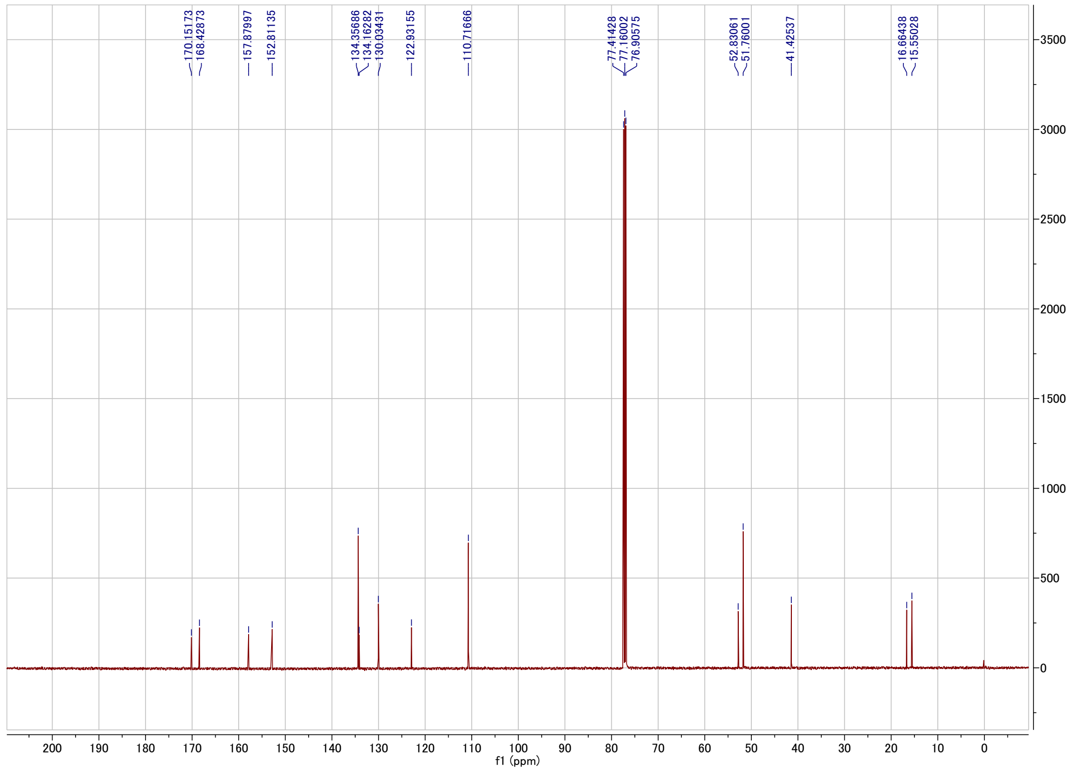

ESI-TOF-MS

Analysis Info

Analysis Name	D:\Data\yuasa_lab\hida\210902\TK230-000001.d	Acquisition Date	2021/09/02 9:01:55
Method	esi_posi_low.m	Operator	BDAL@DE
Sample Name	TK230-	Instrument / Ser#	micrOTOF 213750.10321
Comment			

Acquisition Parameter

Source Type	ESI	Ion Polarity	Positive	Set Nebulizer	0.3 Bar
Focus	Not active			Set Dry Heater	180 ‹C
Scan Begin	50 m/z	Set Capillary	4500 V	Set Dry Gas	4.0 l/min
Scan End	1000 m/z	Set End Plate Offset	-500 V	Set Divert Valve	Waste

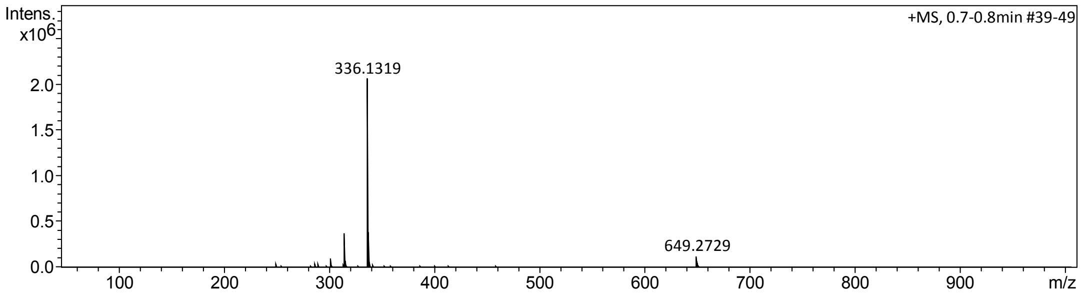

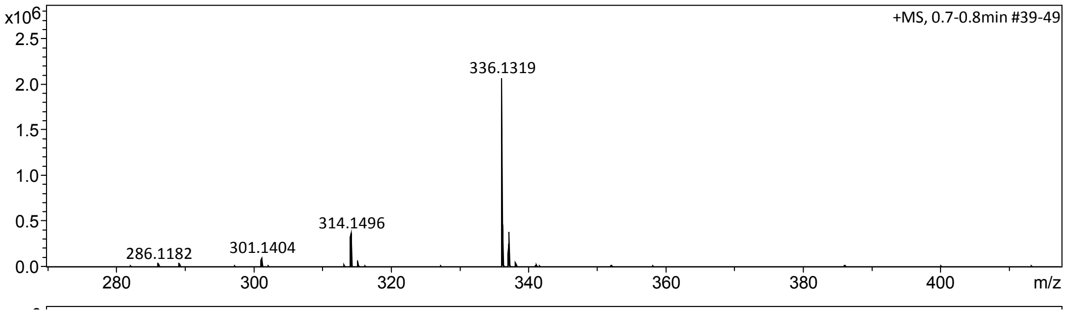

PyrroB-Me

1H-NMR

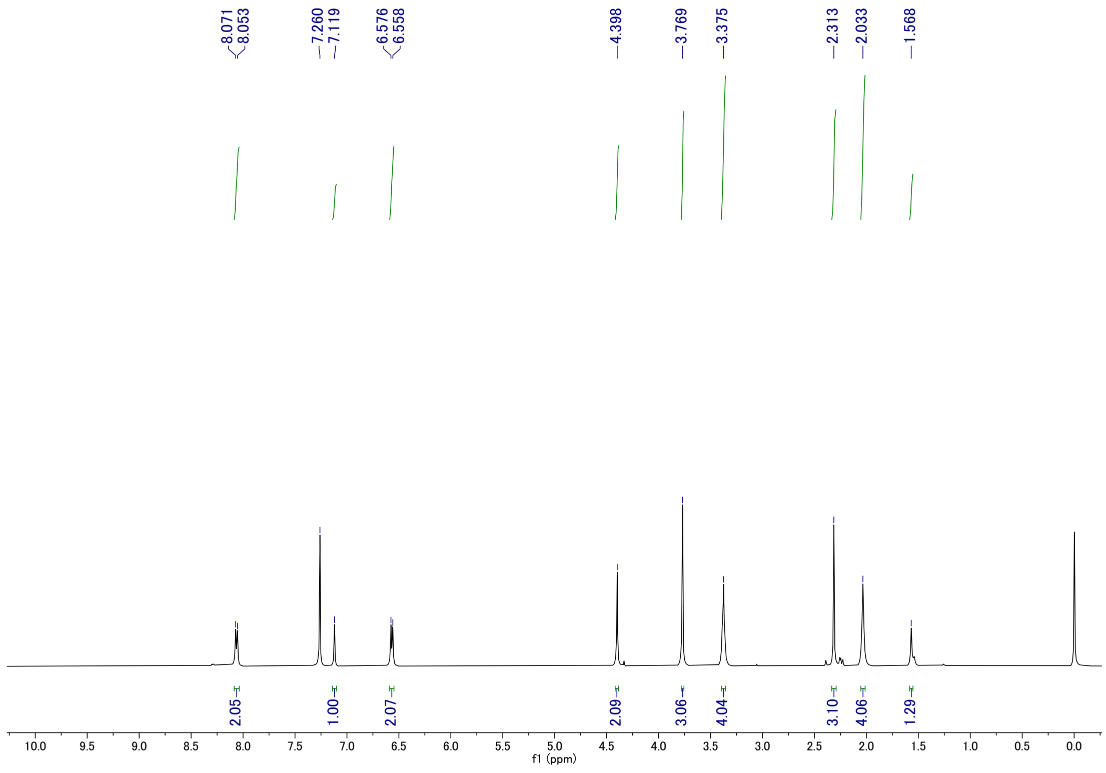

13C-NMR

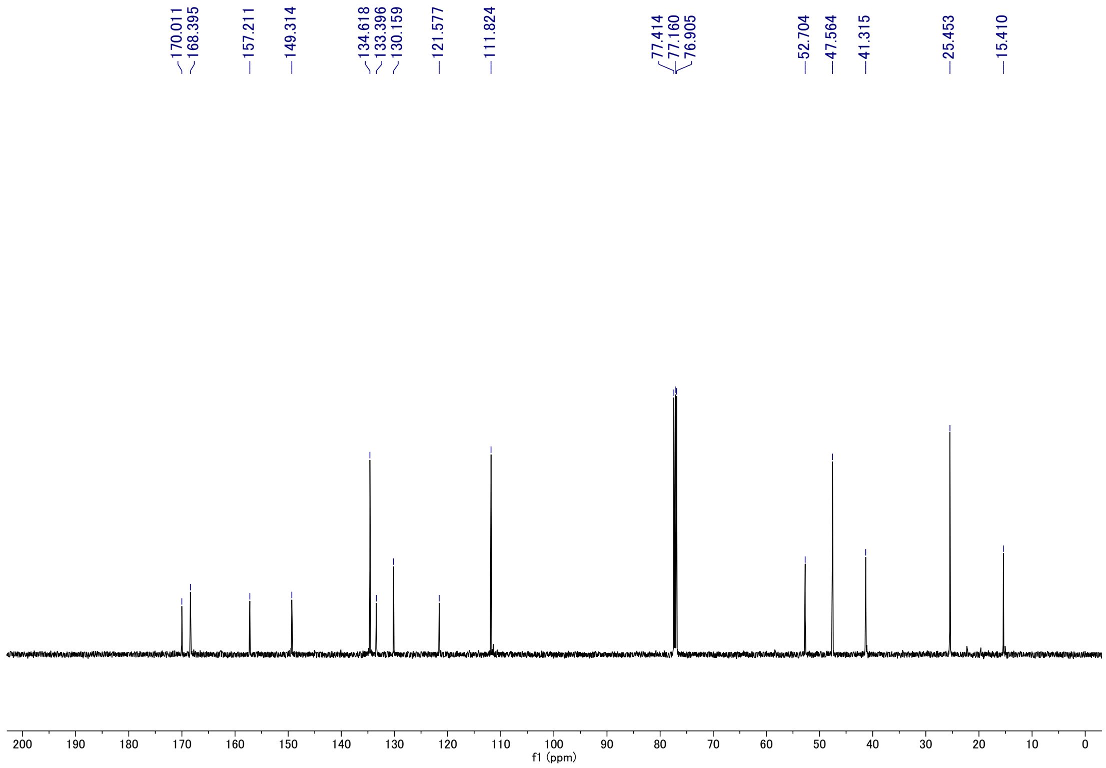

ESI-TOF-MS

Analysis Info

Analysis Name	D:\Data\yuasa_lab\tsuduki\181212\TK208-000001.d	Acquisition Date	2018/12/11 9:51:08	
Method	esi_posi_low.m	Operator	BDAL@DE	
Sample Name	TK208-	Instrument / Ser#	micrOTOF	213750.10321
Comment				

Acquisition Parameter

Source Type	ESI	Ion Polarity	Positive	Set Nebulizer	0.3 Bar
Focus	Not active			Set Dry Heater	180 ‹C
Scan Begin	50 m/z	Set Capillary	4500 V	Set Dry Gas	4.0 l/min
Scan End	1200 m/z	Set End Plate Offset	-500 V	Set Divert Valve	Waste

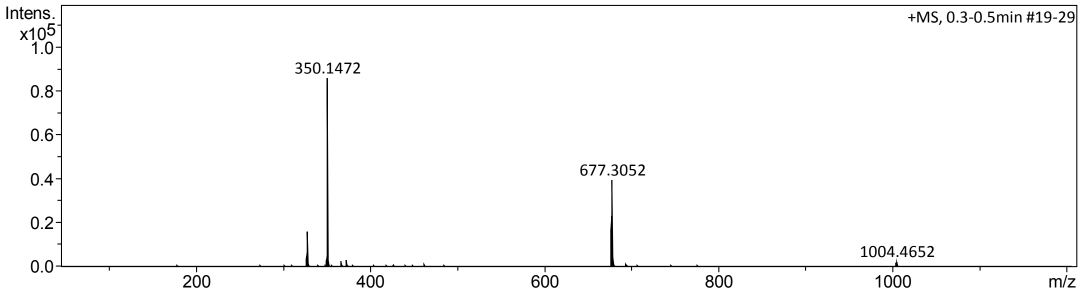

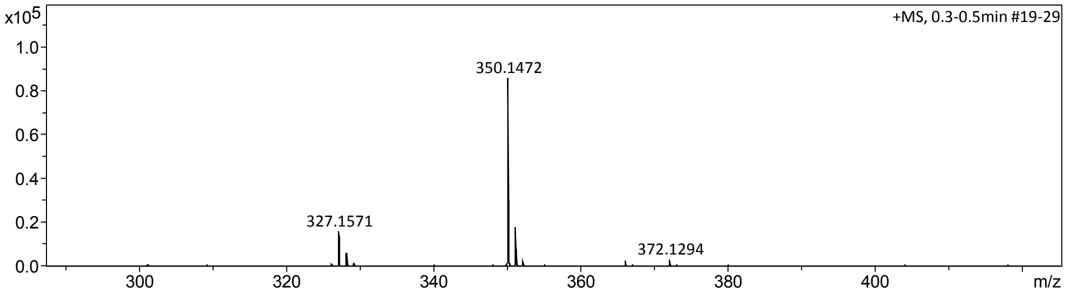

PiperiB-Me

1H-NMR

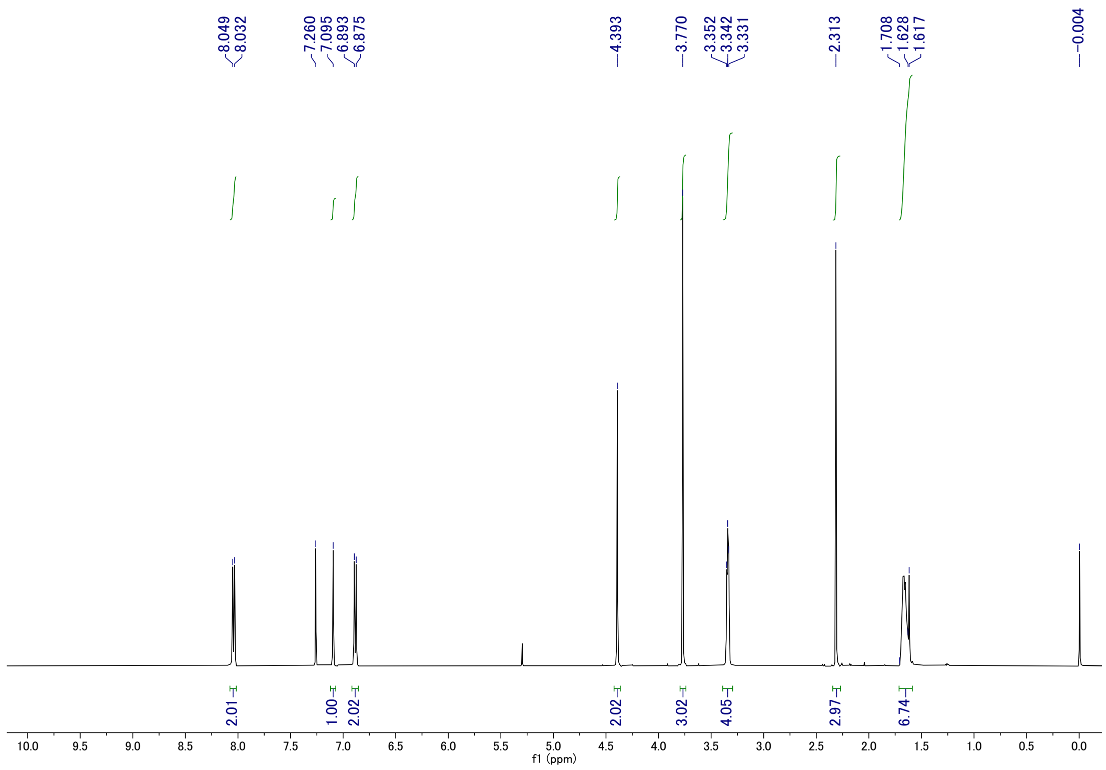

13C-NMR

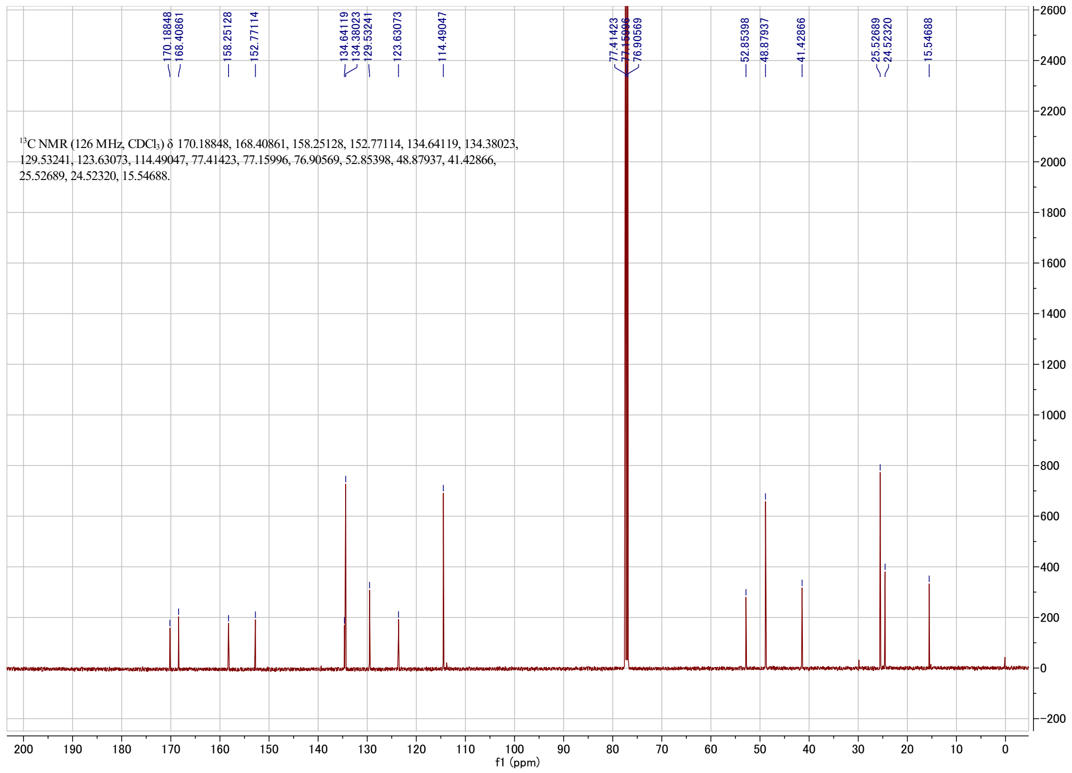

ESI-TOF-MS

Analysis Info

Analysis Name	D:\Data\yuasa_lab\hida\210902\TK231-000001.d	Acquisition Date	2021/09/01 9:31:17
Method	esi_posi_low.m	Operator	BDAL@DE
Sample Name	TK231-	Instrument / Ser#	micrOTOF 213750.10321
Comment			

Acquisition Parameter

Source Type	ESI	Ion Polarity	Positive	Set Nebulizer	0.3 Bar
Focus	Not active			Set Dry Heater	180 ‹C
Scan Begin	50 m/z	Set Capillary	4500 V	Set Dry Gas	4.0 l/min
Scan End	1000 m/z	Set End Plate Offset	-500 V	Set Divert Valve	Waste

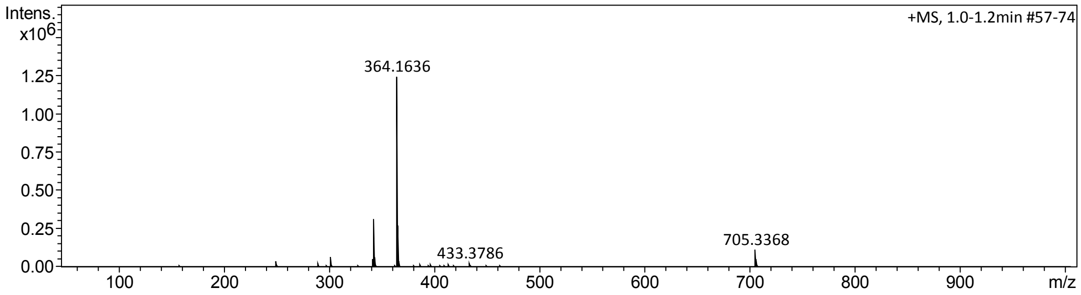

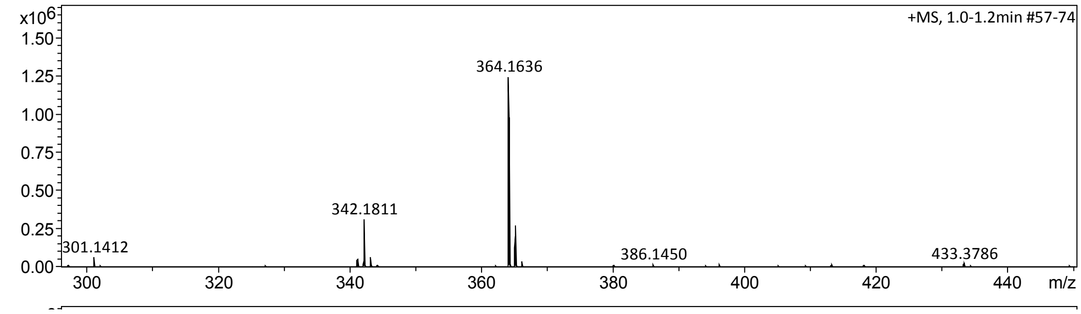

MorphoB-Me

1H-NMR

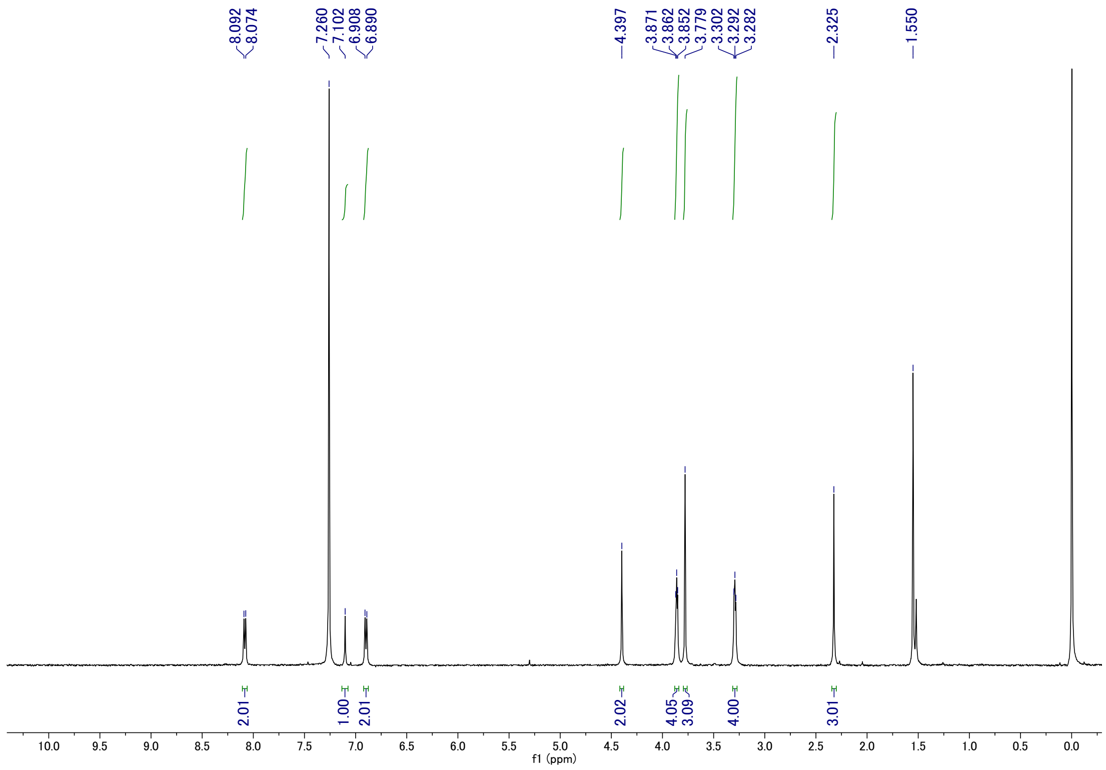

13C-NMR

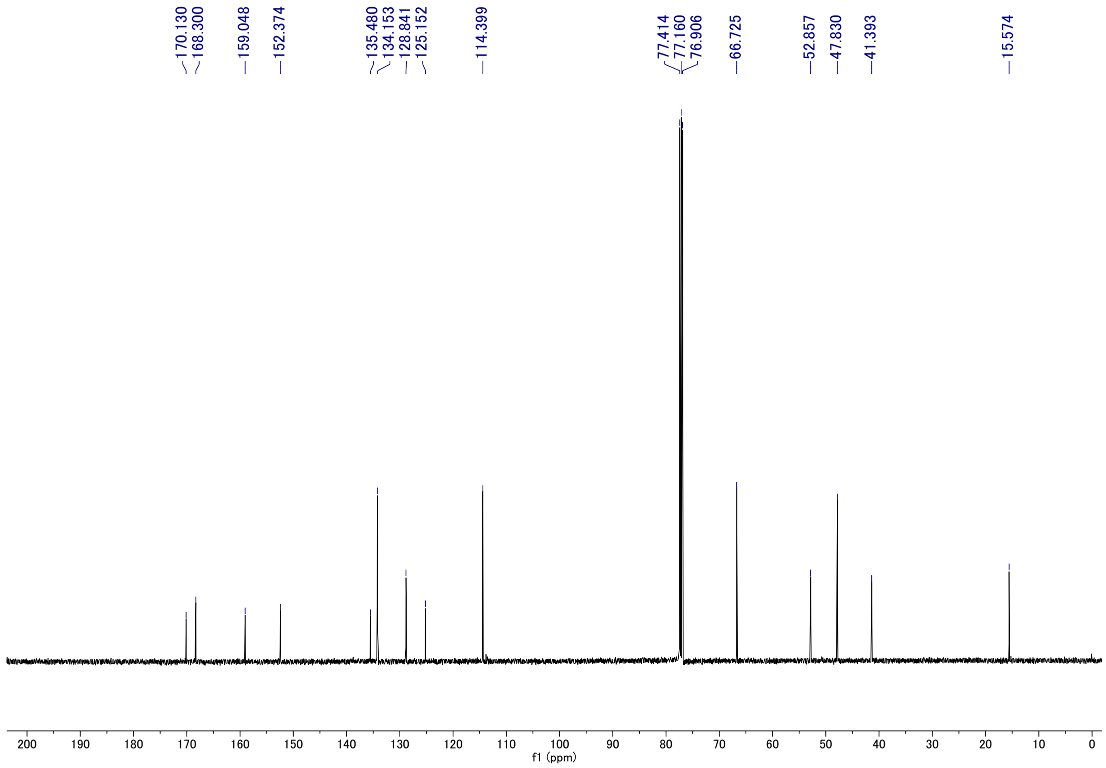

ESI-TOF-MS

Analysis Info

Analysis Name	D:\Data\yuasa_lab\tsuduki\181212\TK209-000001.d	Acquisition Date	2018/12/11 10:07:15
Method	esi_posi_low.m	Operator	BDAL@DE
Sample Name	TK209-	Instrument / Ser#	micrOTOF 213750.10321
Comment			

Acquisition Parameter

Source Type	ESI	Ion Polarity	Positive	Set Nebulizer	0.3 Bar
Focus	Not active			Set Dry Heater	180 ‹C
Scan Begin	50 m/z	Set Capillary	4500 V	Set Dry Gas	4.0 l/min
Scan End	1200 m/z	Set End Plate Offset	-500 V	Set Divert Valve	Waste

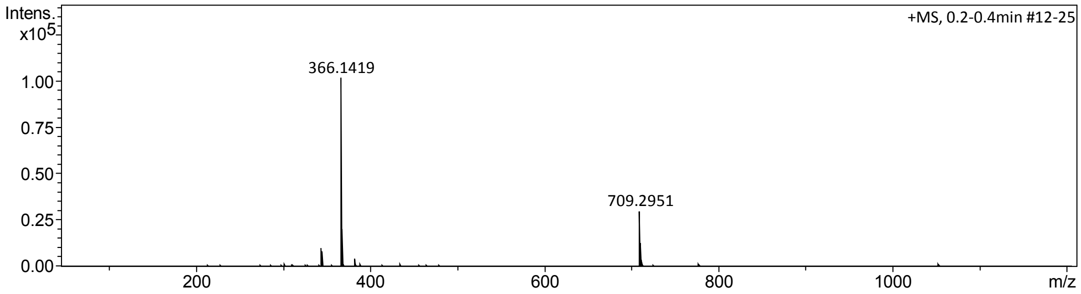

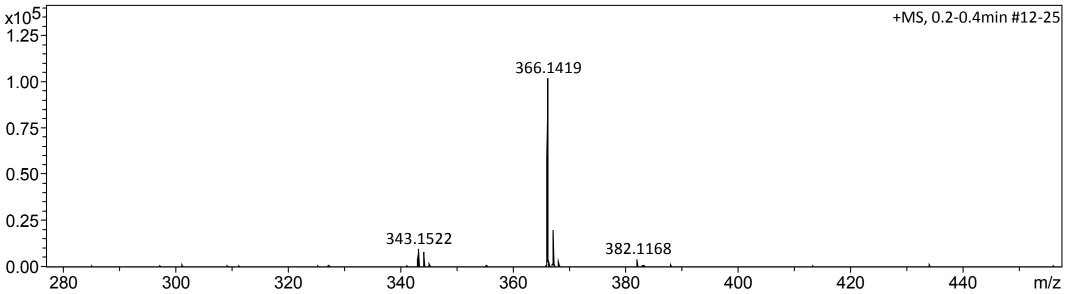

Julo-Me

1H-NMR

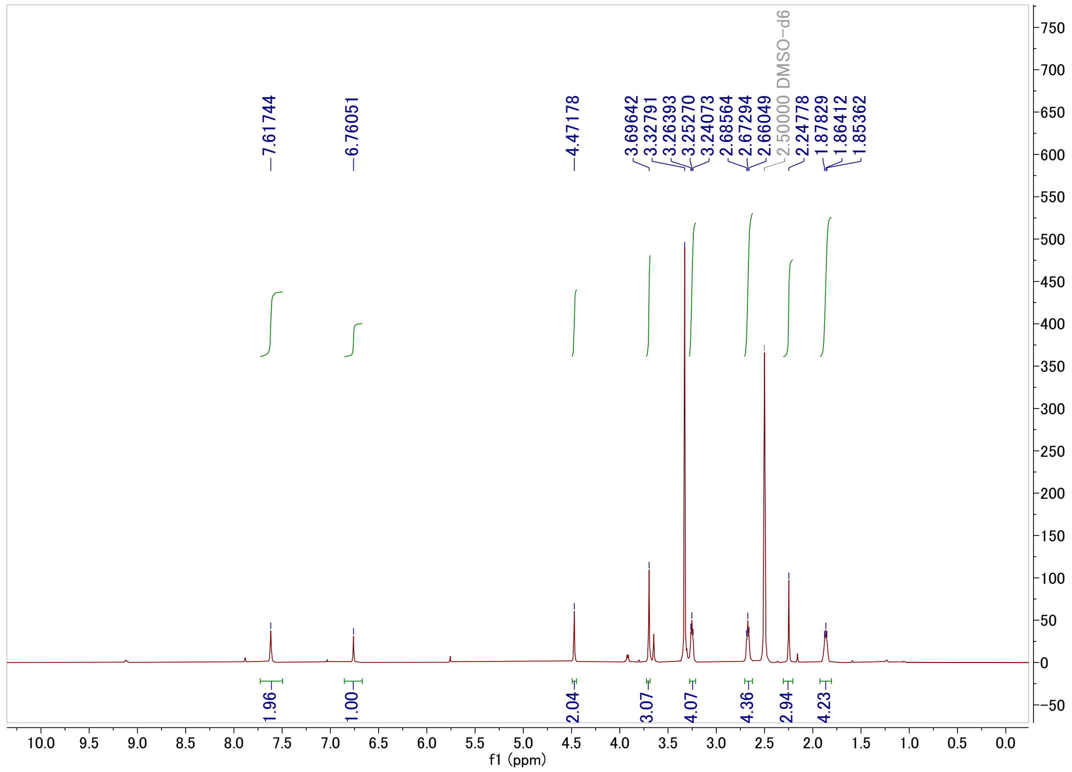

13C-NMR

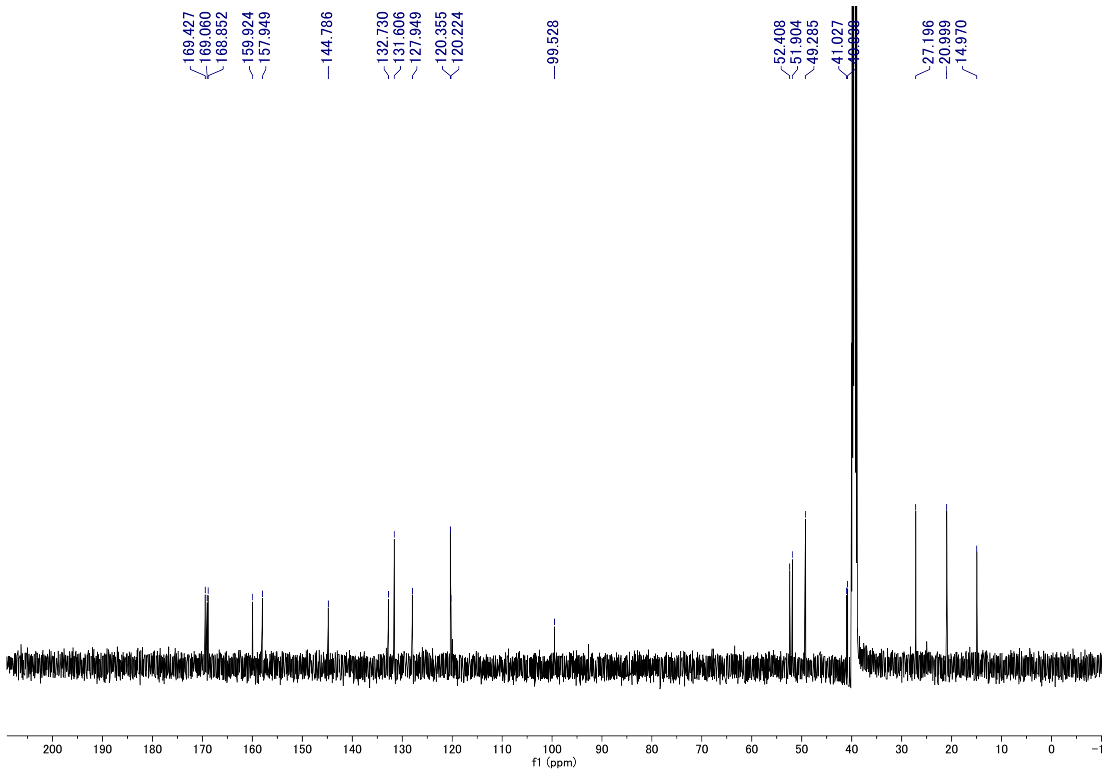

ESI-TOF-MS

Analysis Info

Analysis Name	D:\Data\yuasa_lab\tsuduki\181212\TK211-000001.d	Acquisition Date	2018/12/11 10:15:27
Method	esi_posi_low.m	Operator	BDAL@DE
Sample Name	TK211-	Instrument / Ser#	micrOTOF 213750.10321
Comment			

Acquisition Parameter

Source Type	ESI	Ion Polarity	Positive	Set Nebulizer	0.3 Bar
Focus	Not active			Set Dry Heater	180 ‹C
Scan Begin	50 m/z	Set Capillary	4500 V	Set Dry Gas	4.0 l/min
Scan End	1200 m/z	Set End Plate Offset	-500 V	Set Divert Valve	Waste

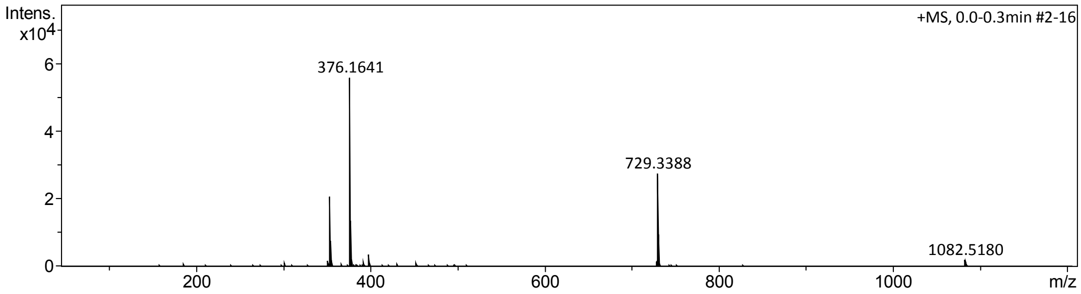

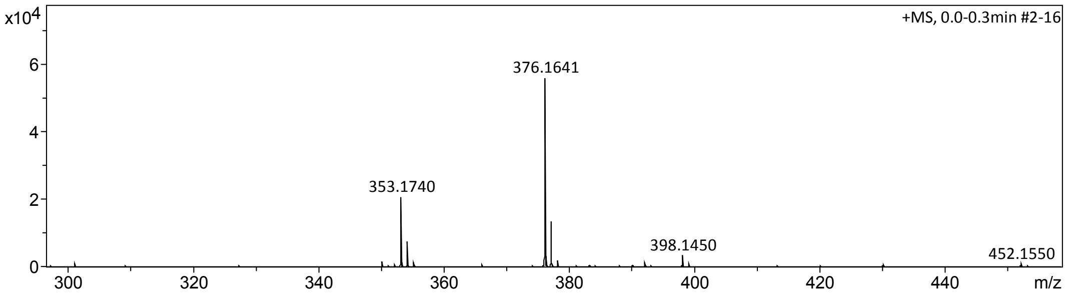

OMeJulo-Me

1H-NMR

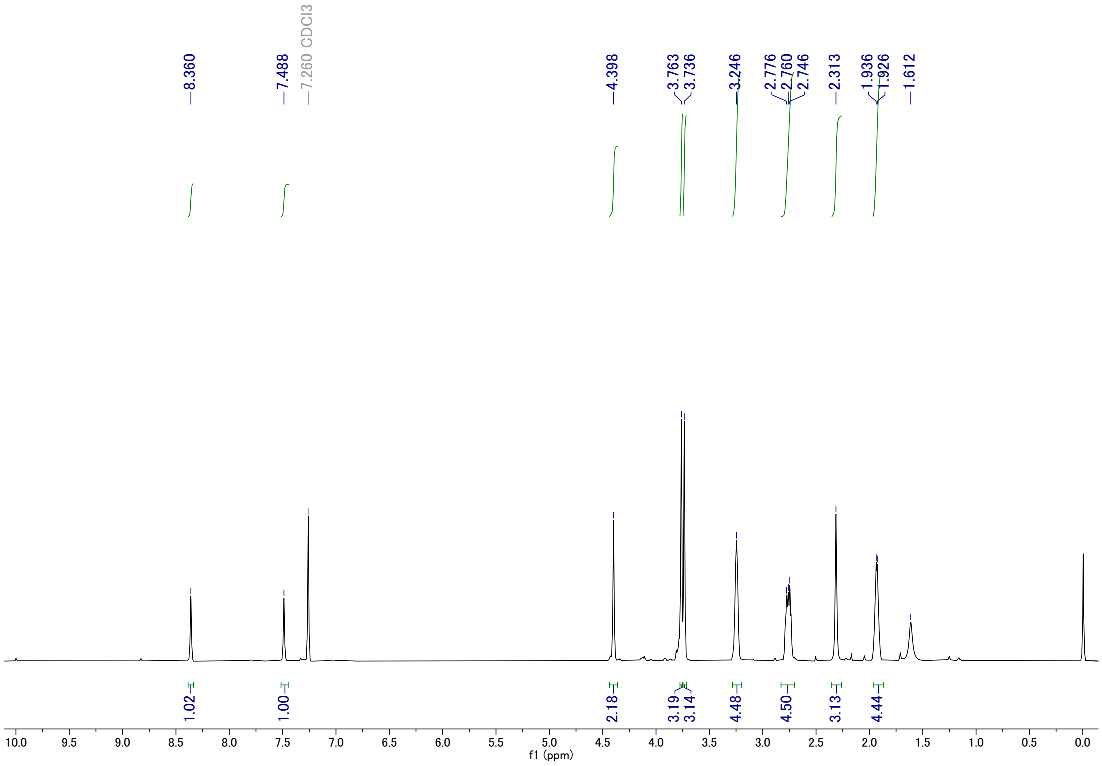

13C-NMR

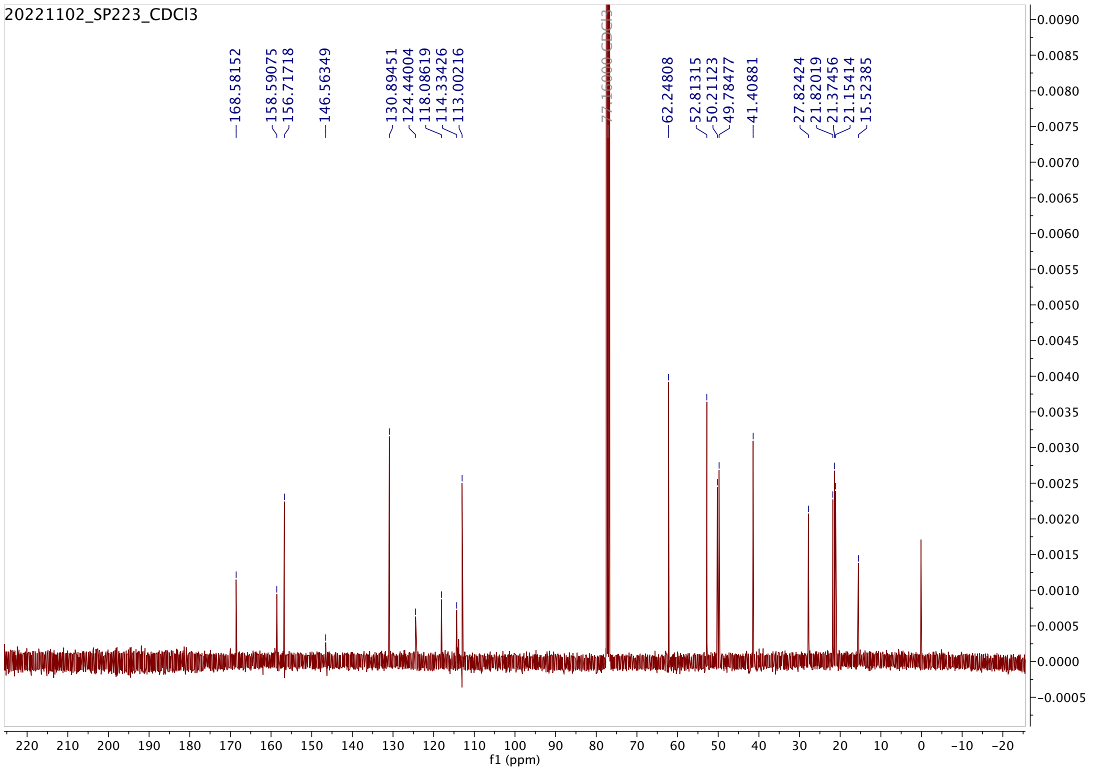

ESI-TOF-MS

Analysis Info

		Acquisition Date	2022/11/15 10:24:18
Analysis Name	D:\Data\ofcbunseki\irai\2022\yuasa_lab\sadai\211116\SP223-000001.d		
Method	esi_posi_low.m	Operator	BDAL@DE
Sample Name	SP223-	Instrument / Ser#	micrOTOF 213750.10321
Comment			

Acquisition Parameter

Source Type	ESI	Ion Polarity	Positive	Set Nebulizer	0.3 Bar
Focus	Not active			Set Dry Heater	180 ‹C
Scan Begin	50 m/z	Set Capillary	4500 V	Set Dry Gas	4.0 l/min
Scan End	1200 m/z	Set End Plate Offset	-500 V	Set Divert Valve	Waste

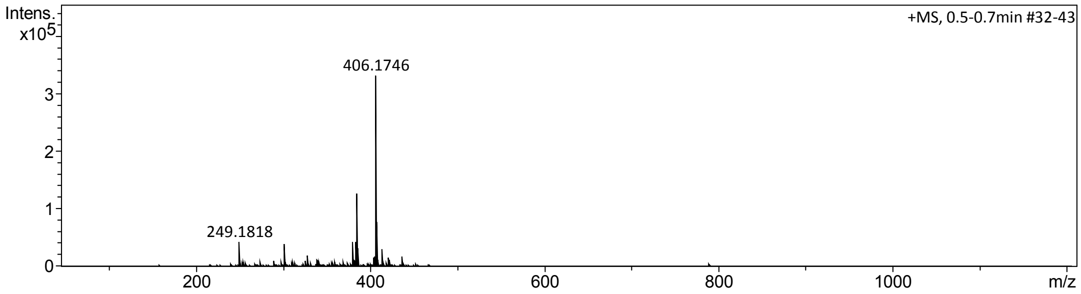

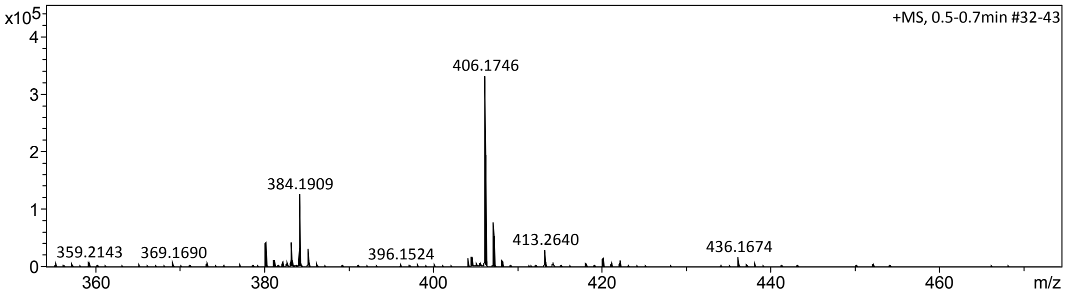

DPhAB-Me

1H-NMR

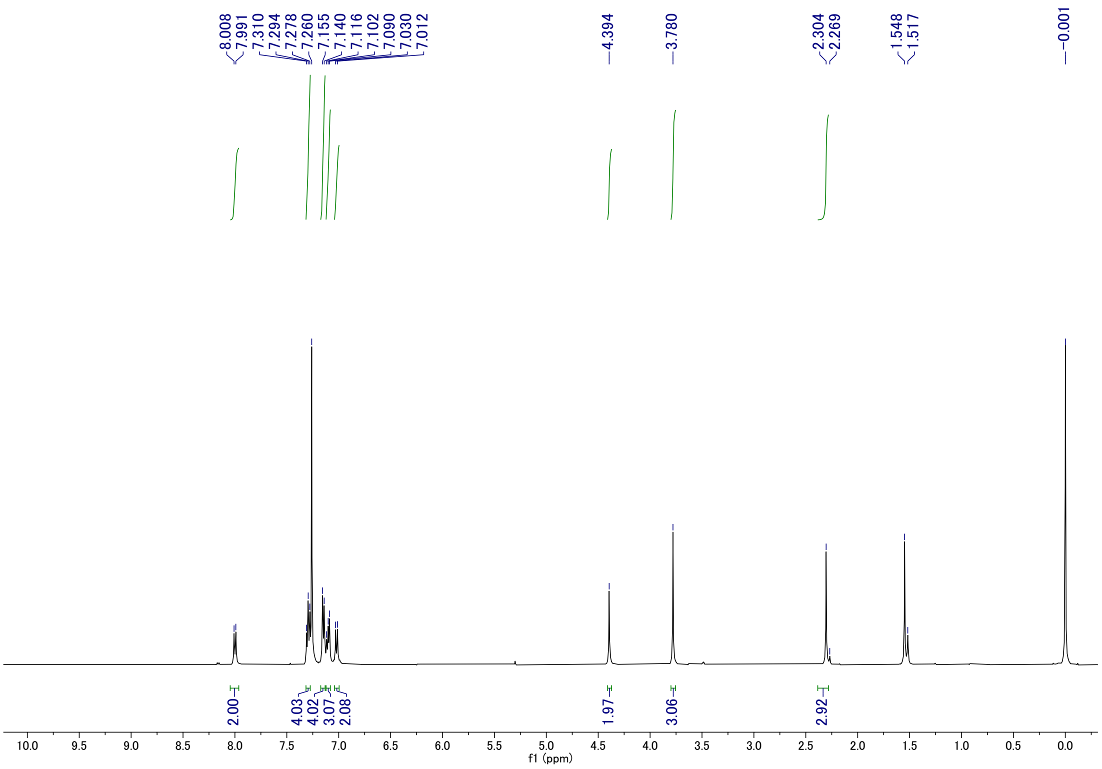

13C-NMR

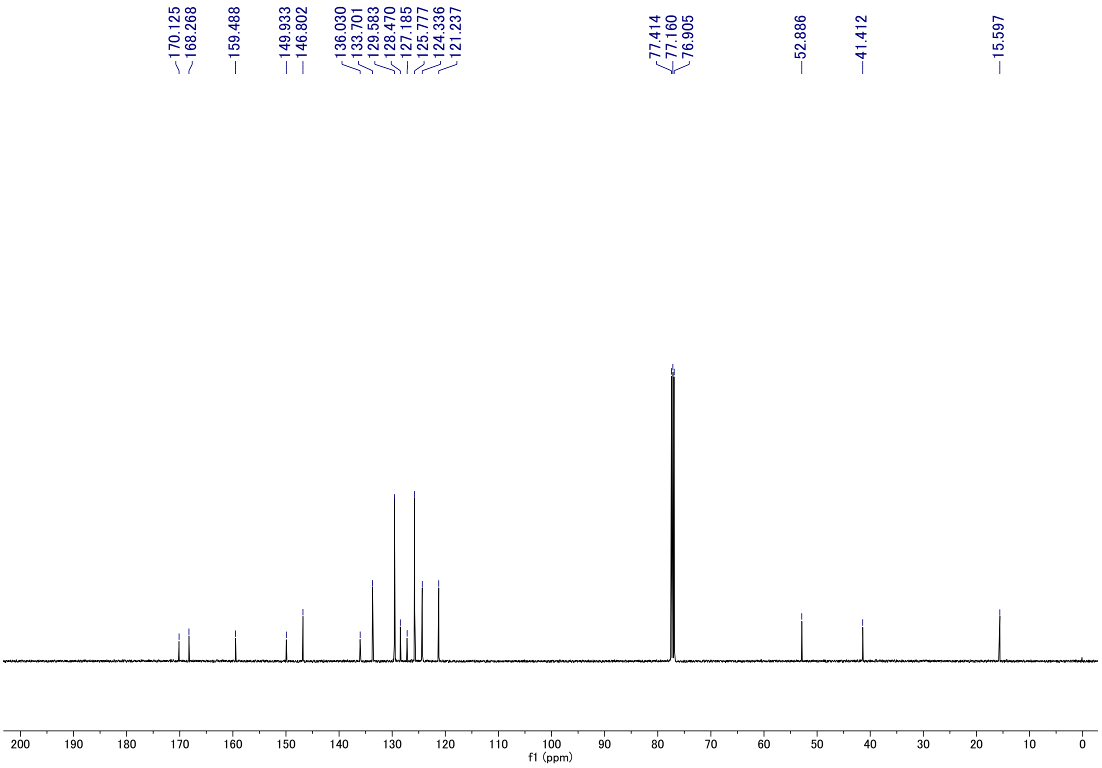

ESI-TOF-MS

**Analysis Info**		Acquisition Date	2018/12/11 9:37:52	
Analysis Name	D:\Data\yuasa_lab\tsuduki\181212\TK207-000001.d			
Method	esi_posi_low.m	Operator	BDAL@DE	
Sample Name	TK207-	Instrument / Ser#	micrOTOF	213750.10321
Comment				

Acquisition Parameter

Source Type	ESI	Ion Polarity	Positive	Set Nebulizer	0.3 Bar
Focus	Not active			Set Dry Heater	180 ‹C
Scan Begin	50 m/z	Set Capillary	4500 V	Set Dry Gas	4.0 l/min
Scan End	1200 m/z	Set End Plate Offset	-500 V	Set Divert Valve	Waste

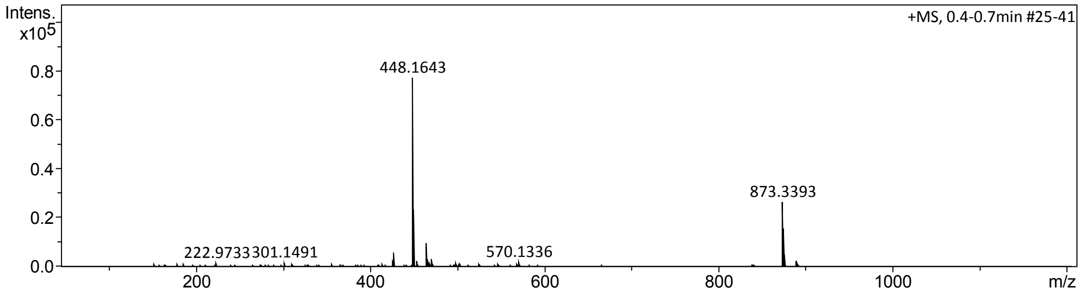

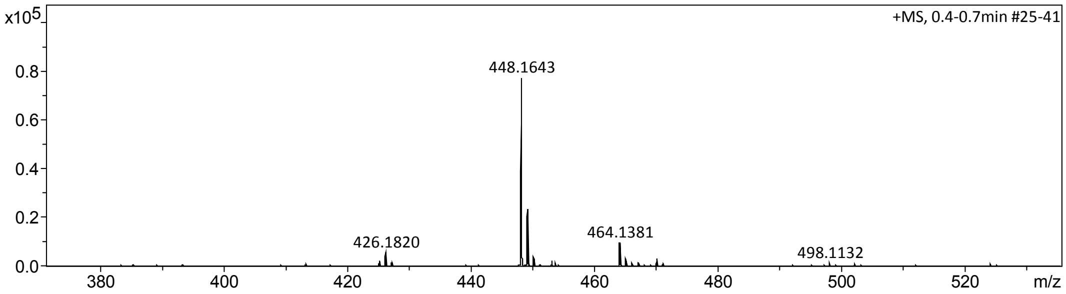

CarbaB-Me

1H-NMR

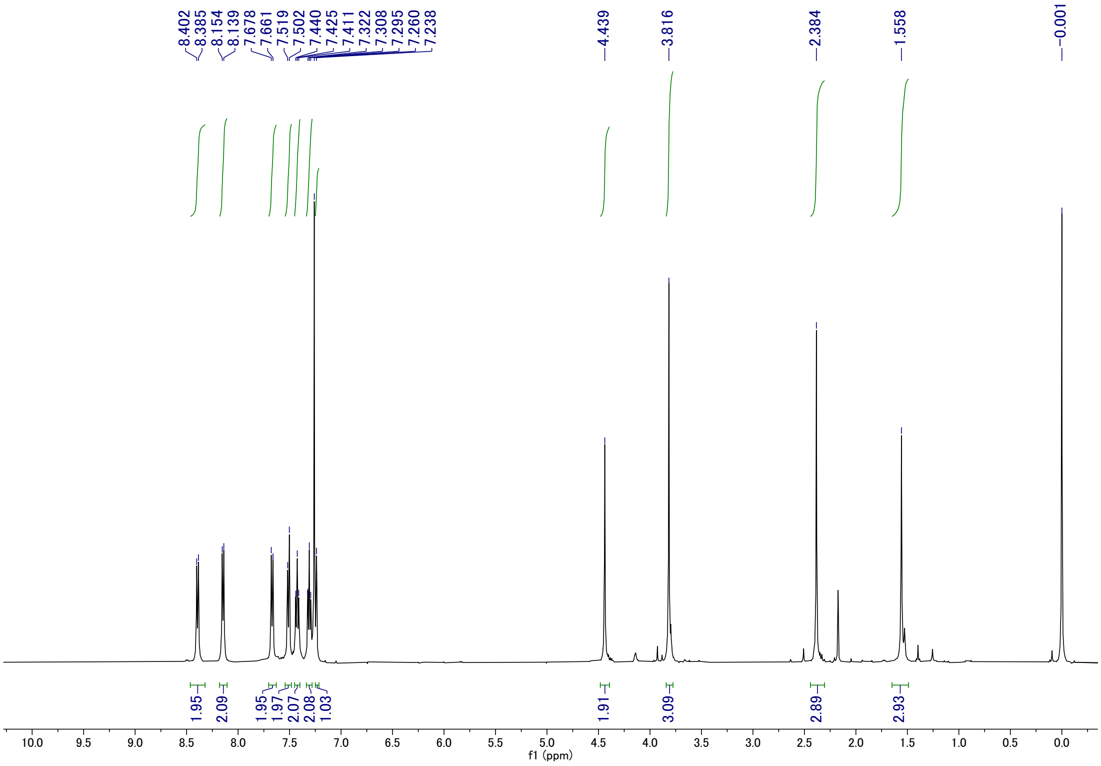

13C-NMR

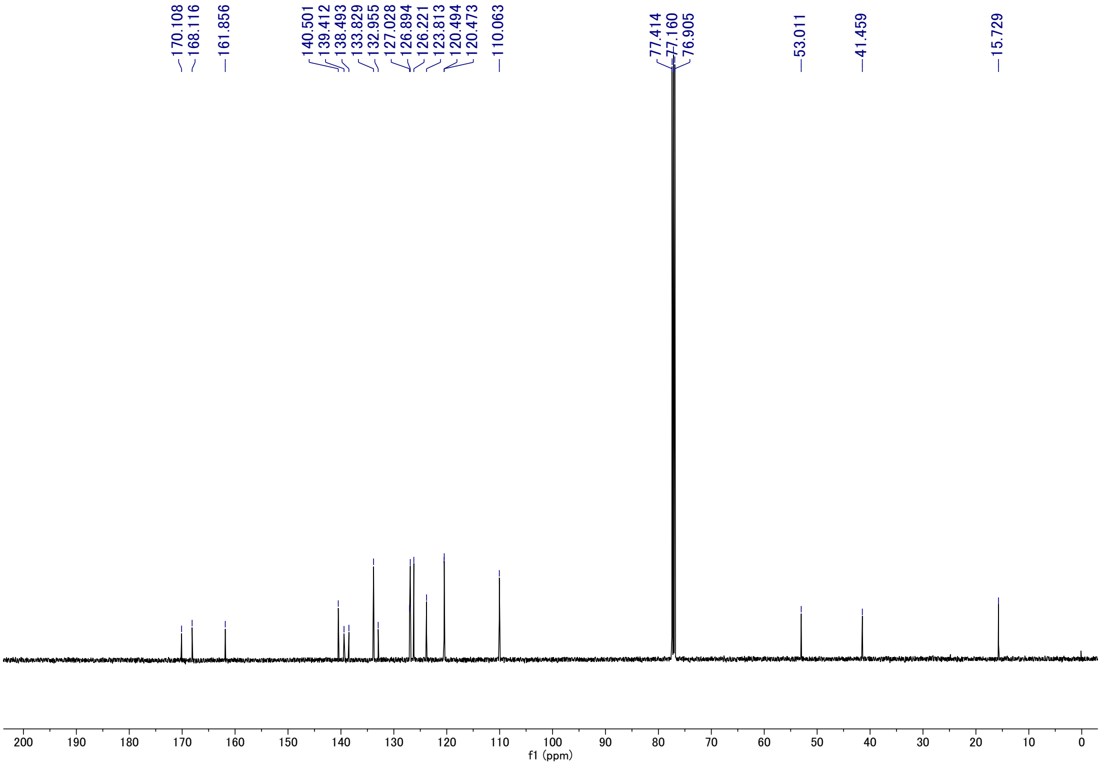

ESI-TOF-MS

Analysis Info

Analysis Name	D:\Data\yuasa_lab\tsuduki\181212\TT02048-000001.d	Acquisition Date	2018/12/11 11:02:12
Method	esi_posi_low.m	Operator	BDAL@DE
Sample Name	TT02048-	Instrument / Ser#	micrOTOF 213750.10321
Comment			

Acquisition Parameter

Source Type	ESI	Ion Polarity	Positive	Set Nebulizer	0.3 Bar
Focus	Not active			Set Dry Heater	180 ‹C
Scan Begin	50 m/z	Set Capillary	4500 V	Set Dry Gas	4.0 l/min
Scan End	1200 m/z	Set End Plate Offset	-500 V	Set Divert Valve	Waste

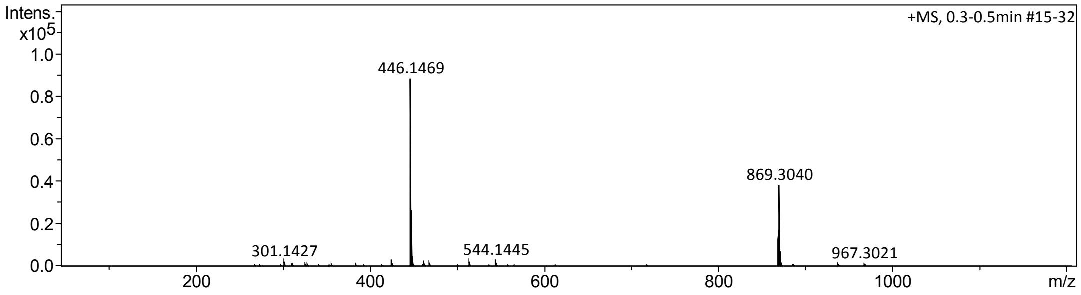

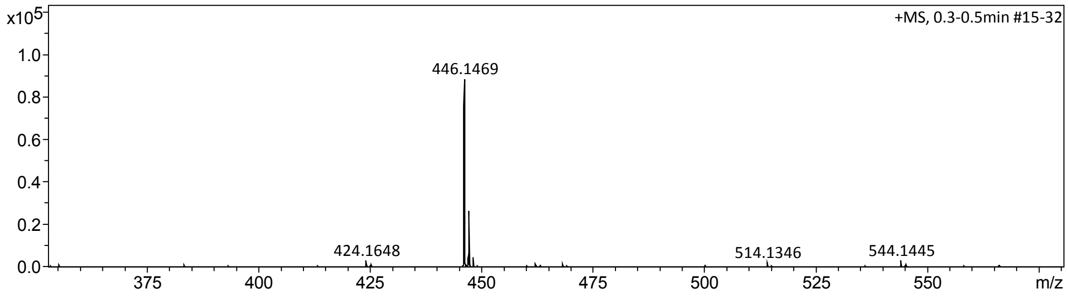

Phcarba-Me

1H-NMR

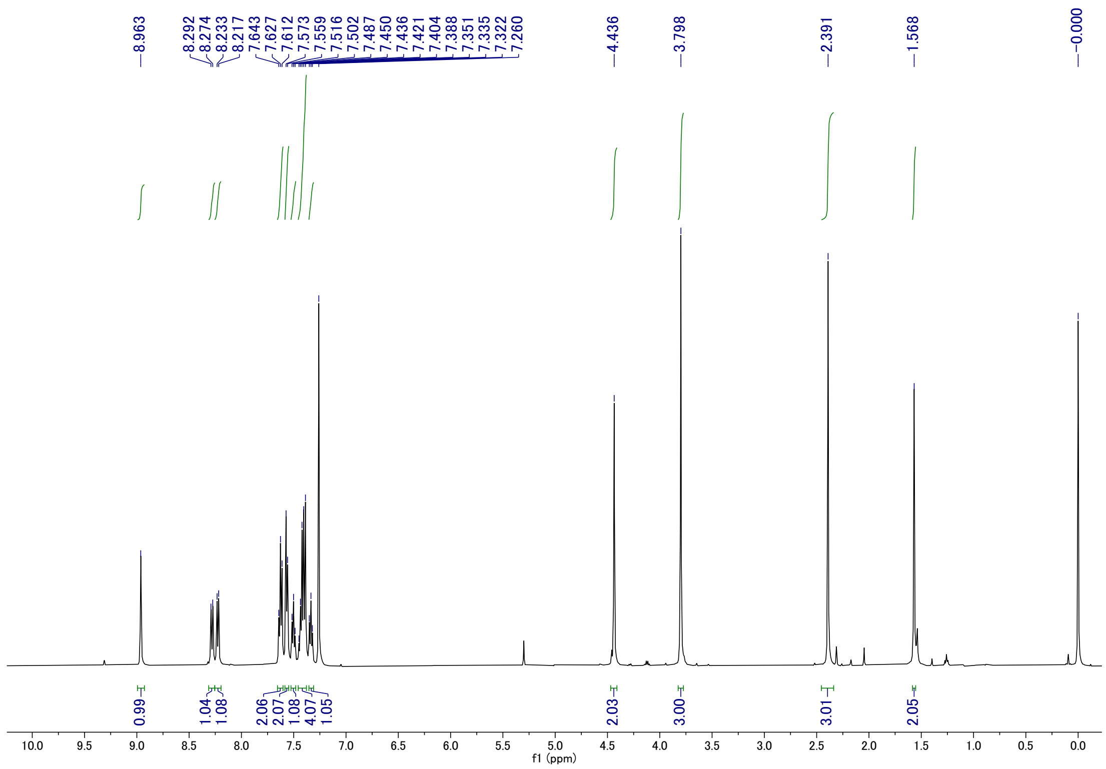

13C-NMR

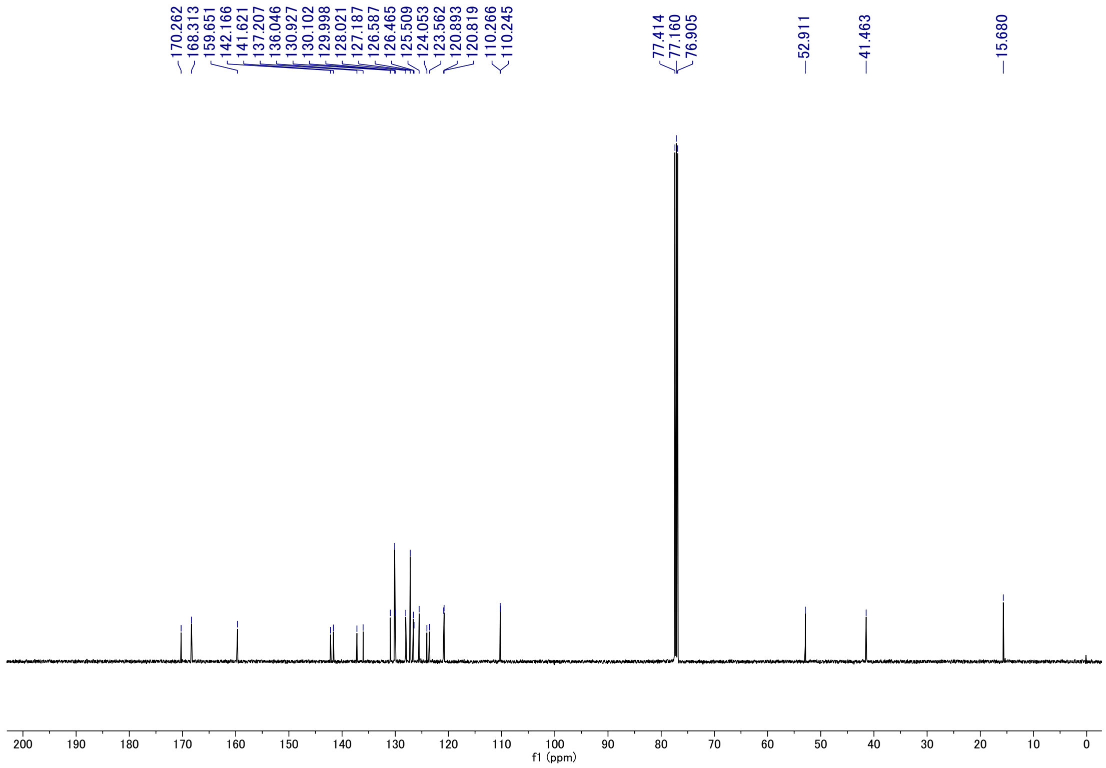

ESI-TOF-MS

Analysis Info

Analysis Name	D:\Data\yuasa_lab\tsuduki\181212\TT02044-000001.d	Acquisition Date	2018/12/11 10:38:26	
Method	esi_posi_low.m	Operator	BDAL@DE	
Sample Name	TT02044-	Instrument / Ser#	micrOTOF	213750.10321
Comment				

Acquisition Parameter

Source Type	ESI	Ion Polarity	Positive	Set Nebulizer	0.3 Bar
Focus	Not active			Set Dry Heater	180 ‹C
Scan Begin	50 m/z	Set Capillary	4500 V	Set Dry Gas	4.0 l/min
Scan End	1200 m/z	Set End Plate Offset	-500 V	Set Divert Valve	Waste

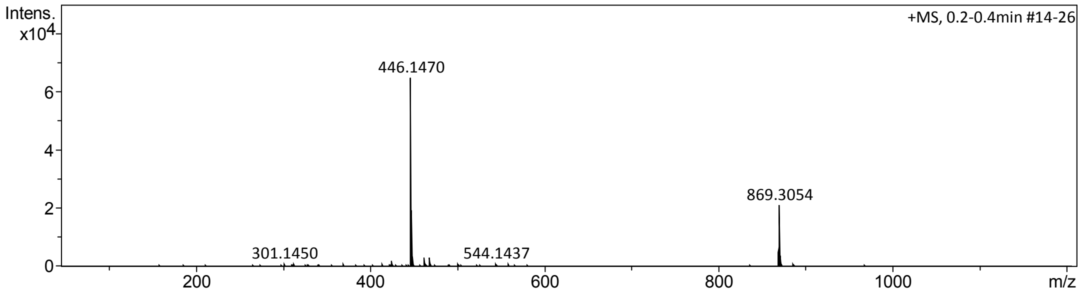

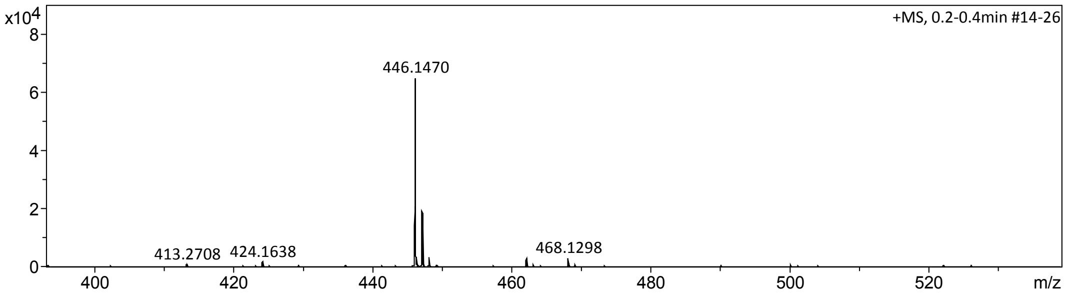

DMeOPhAB-Me

1H-NMR

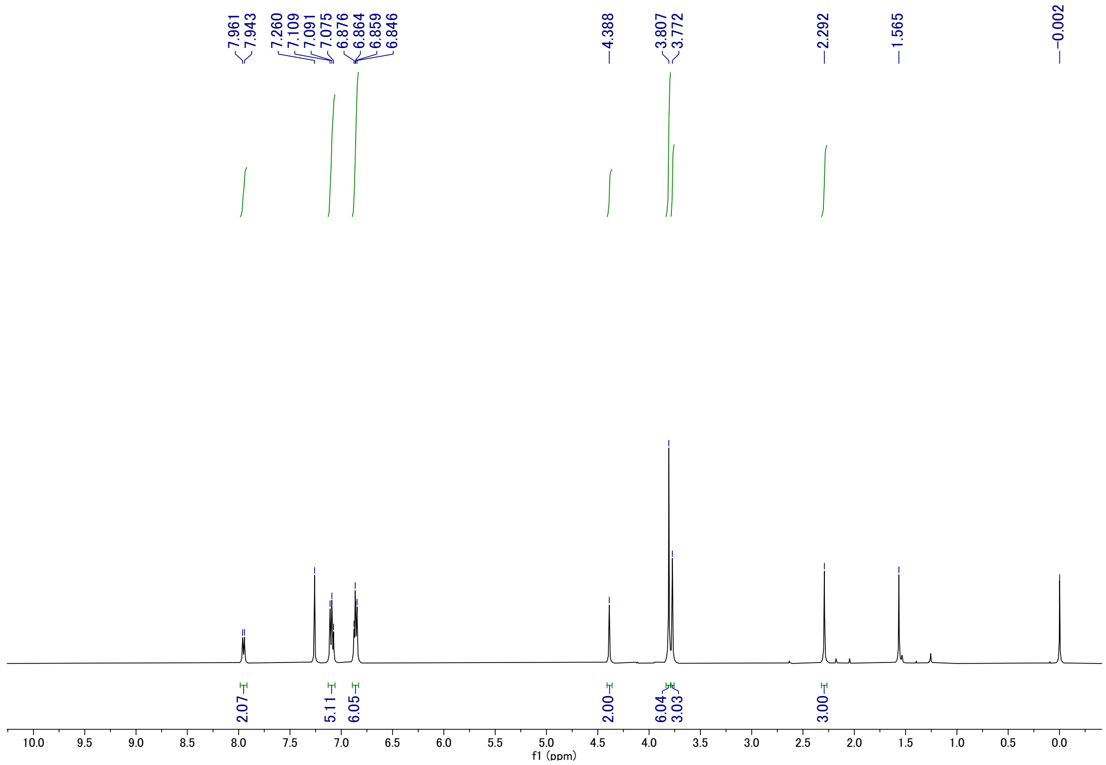

13C-NMR

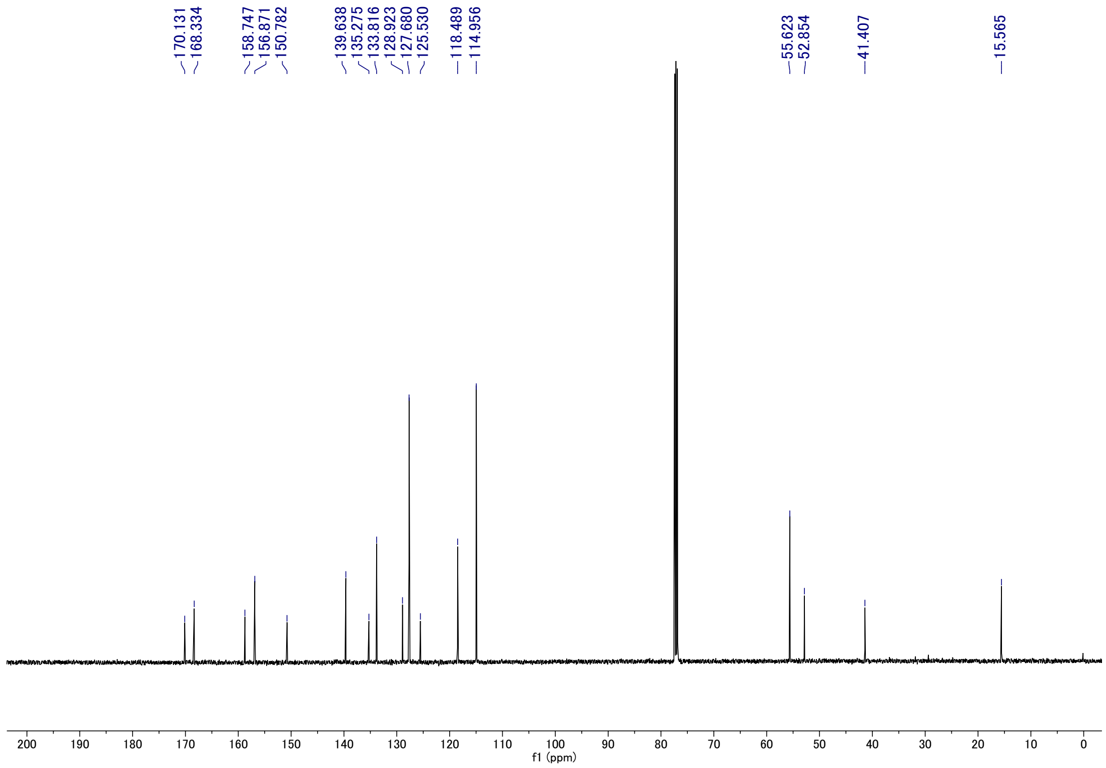

ESI-TOF-MS

Analysis Info

Analysis Name	D:\Data\yuasa_lab\tsuduki\181212\TT02052-000001.d	Acquisition Date	2018/12/11 10:49:07
Method	esi_posi_low.m	Operator	BDAL@DE
Sample Name	TT02052-	Instrument / Ser#	micrOTOF 213750.10321
Comment			

Acquisition Parameter

Source Type	ESI	Ion Polarity	Positive	Set Nebulizer	0.3 Bar
Focus	Not active			Set Dry Heater	180 ‹C
Scan Begin	50 m/z	Set Capillary	4500 V	Set Dry Gas	4.0 l/min
Scan End	1200 m/z	Set End Plate Offset	-500 V	Set Divert Valve	Waste

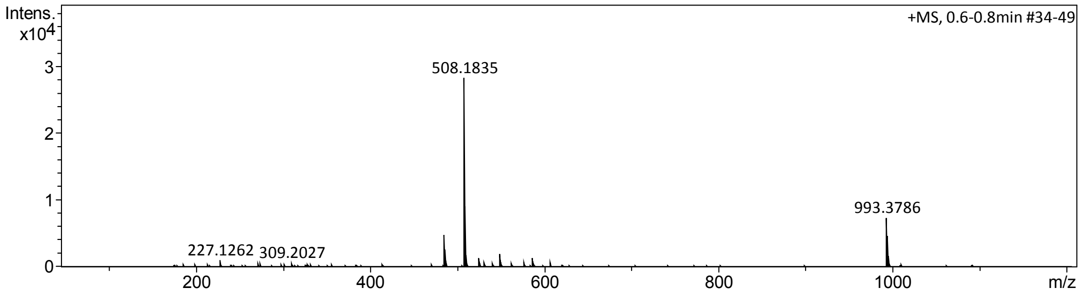

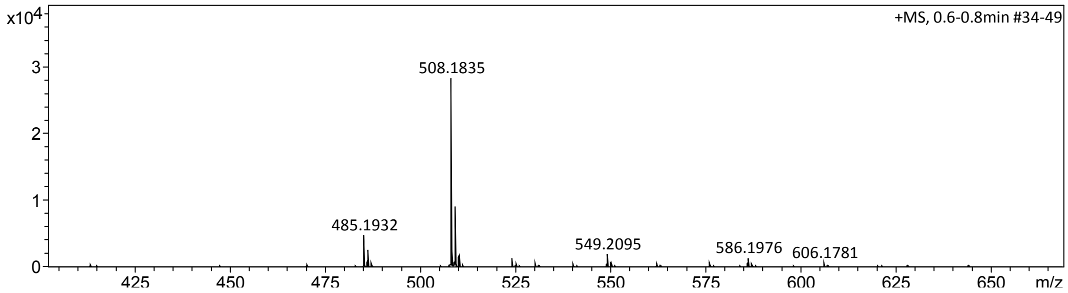

DMAB-Ph-lactone

1H-NMR

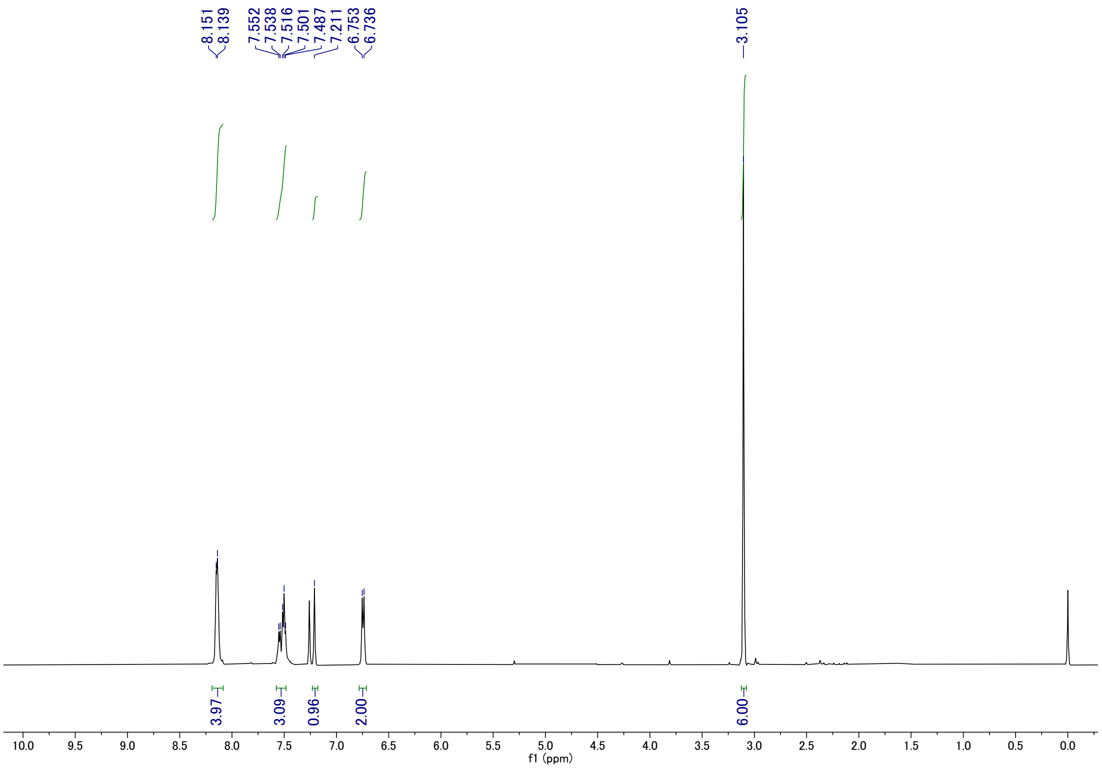

13C-NMR

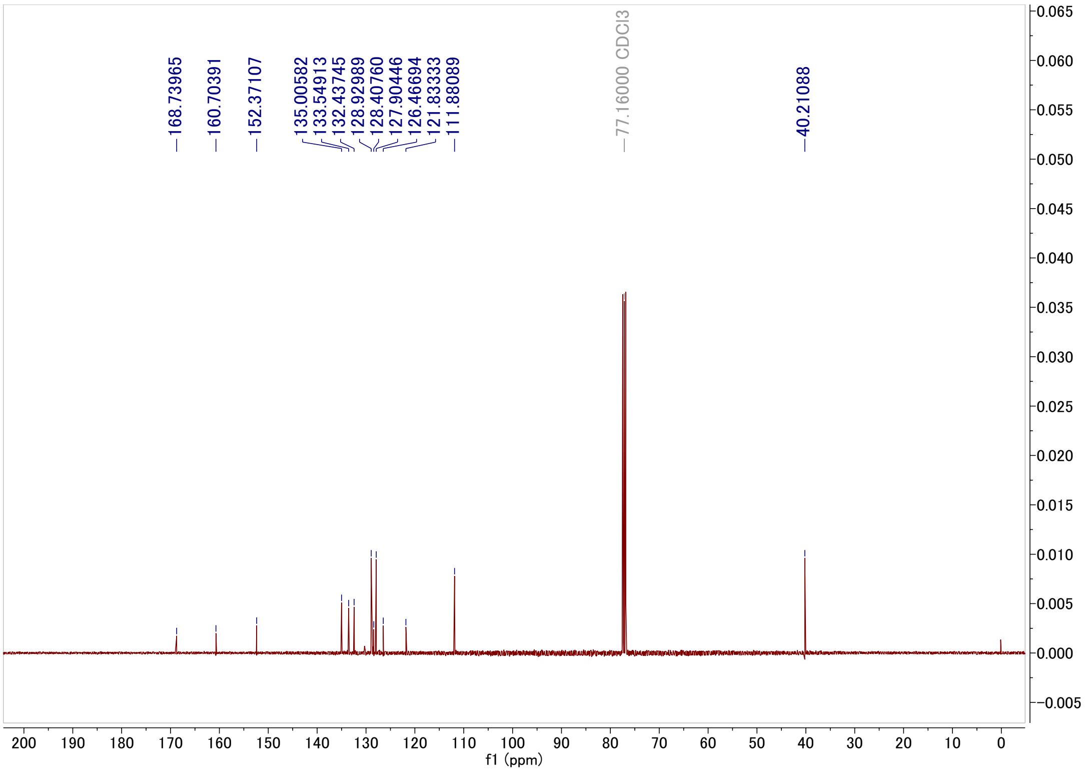

ESI-TOF-MS

TDCMAS ESI-TOF

Analysis Info

Analysis Name	D:\Data\ofcbunseki\irai\2022\yuasa_lab\hida\230216\NOG001-000001.d	Acquisition Date	2023/02/15 8:38:49
Method	esi_posi_low.m	Operator	BDAL@DE
Sample Name	NOG001-	Instrument / Ser#	micrOTOF 213750.10321
Comment			

Acquisition Parameter

Source Type	ESI	Ion Polarity	Positive	Set Nebulizer	0.3 Bar
Focus	Not active			Set Dry Heater	180 ‹C
Scan Begin	50 m/z	Set Capillary	4500 V	Set Dry Gas	4.0 l/min
Scan End	1000 m/z	Set End Plate Offset	-500 V	Set Divert Valve	Waste

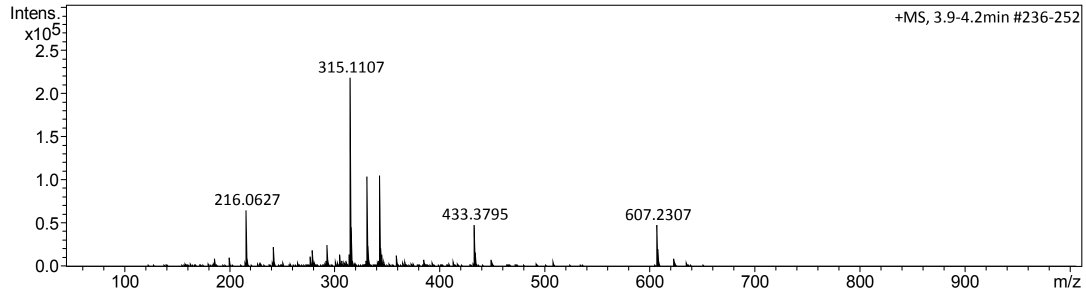

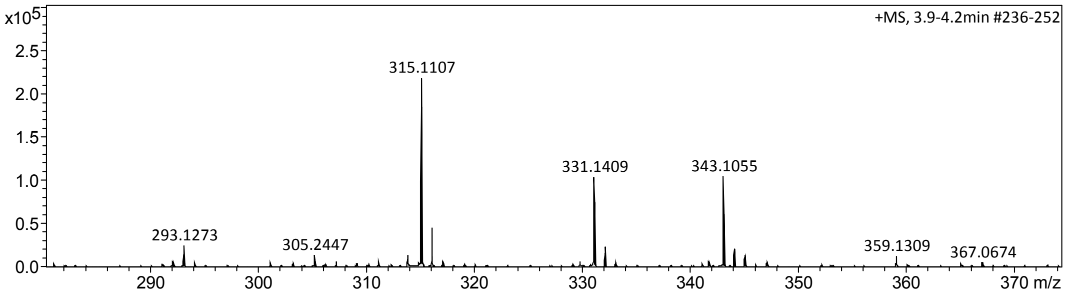

PyrroB-Ph-lactone

1H-NMR

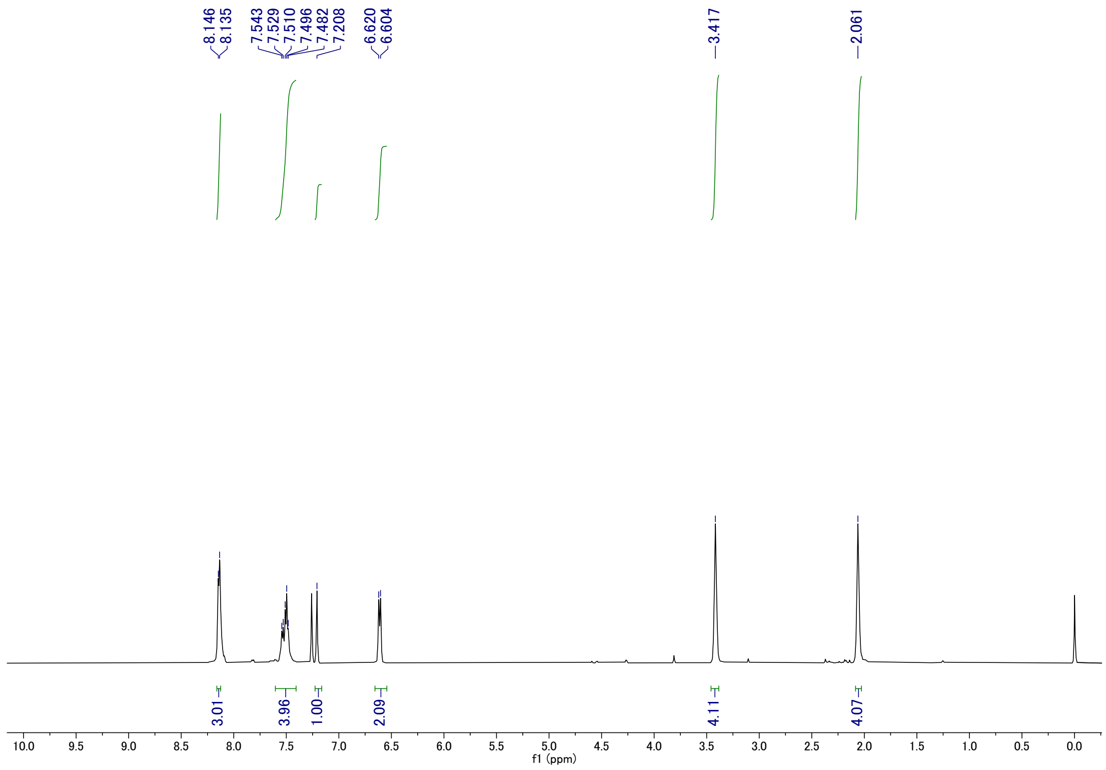

13C-NMR

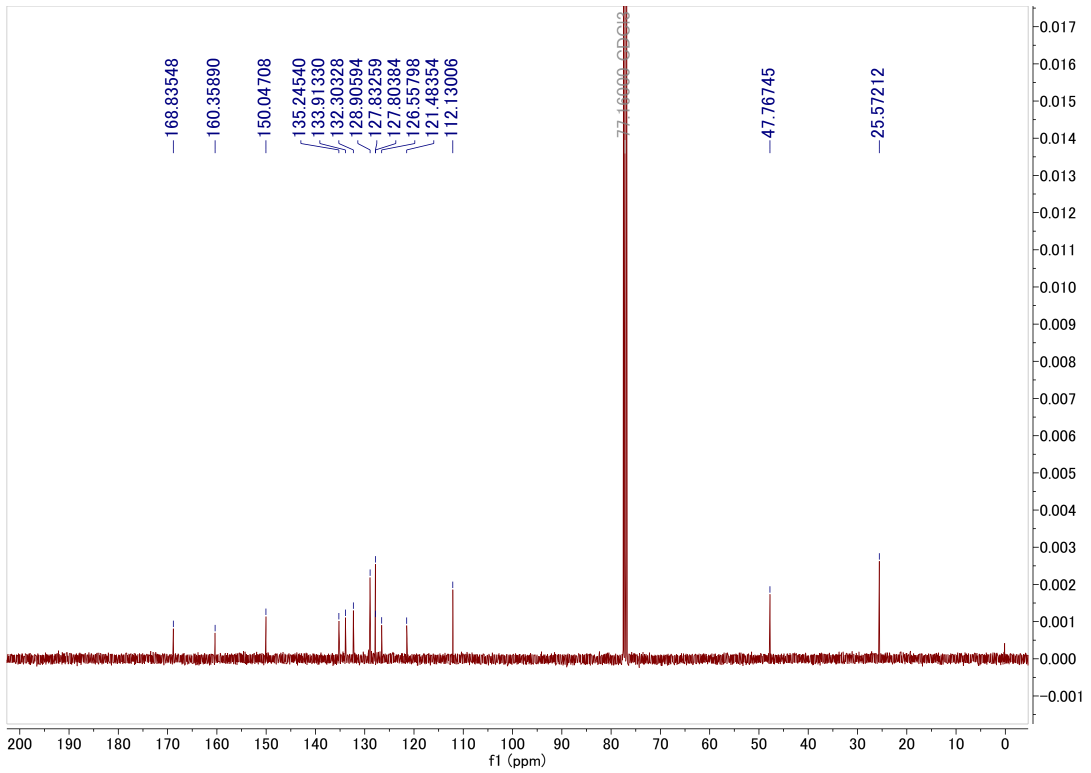

ESI-TOF-MS

Analysis Info

Analysis Name	D:\Data\ofcbunseki\irai\2022\yuasa_lab\sadai\211116\HID108-000001.d	Acquisition Date	2022/11/15 10:44:46
Method	esi_posi_low.m	Operator	BDAL@DE
Sample Name	HID108-	Instrument / Ser#	micrOTOF 213750.10321
Comment			

Acquisition Parameter

Source Type	ESI	Ion Polarity	Positive	Set Nebulizer	0.3 Bar
Focus	Not active			Set Dry Heater	180 ‹C
Scan Begin	50 m/z	Set Capillary	4500 V	Set Dry Gas	4.0 l/min
Scan End	1200 m/z	Set End Plate Offset	-500 V	Set Divert Valve	Waste

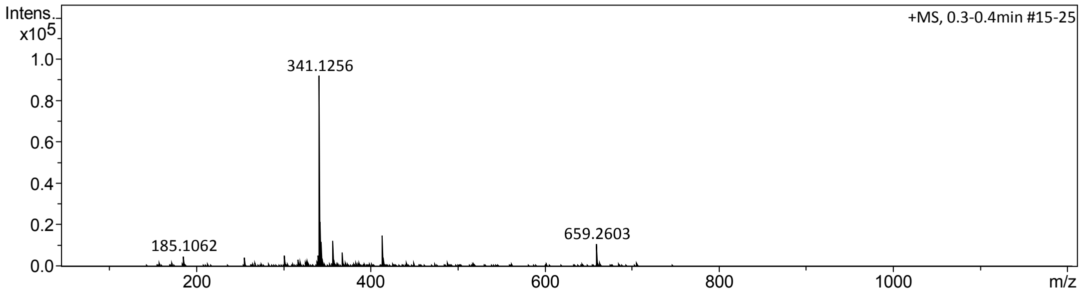

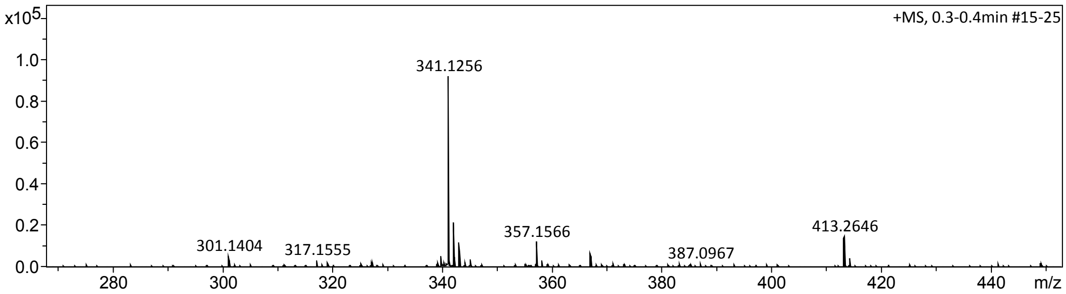

Julo-Ph-lactone

1H-NMR

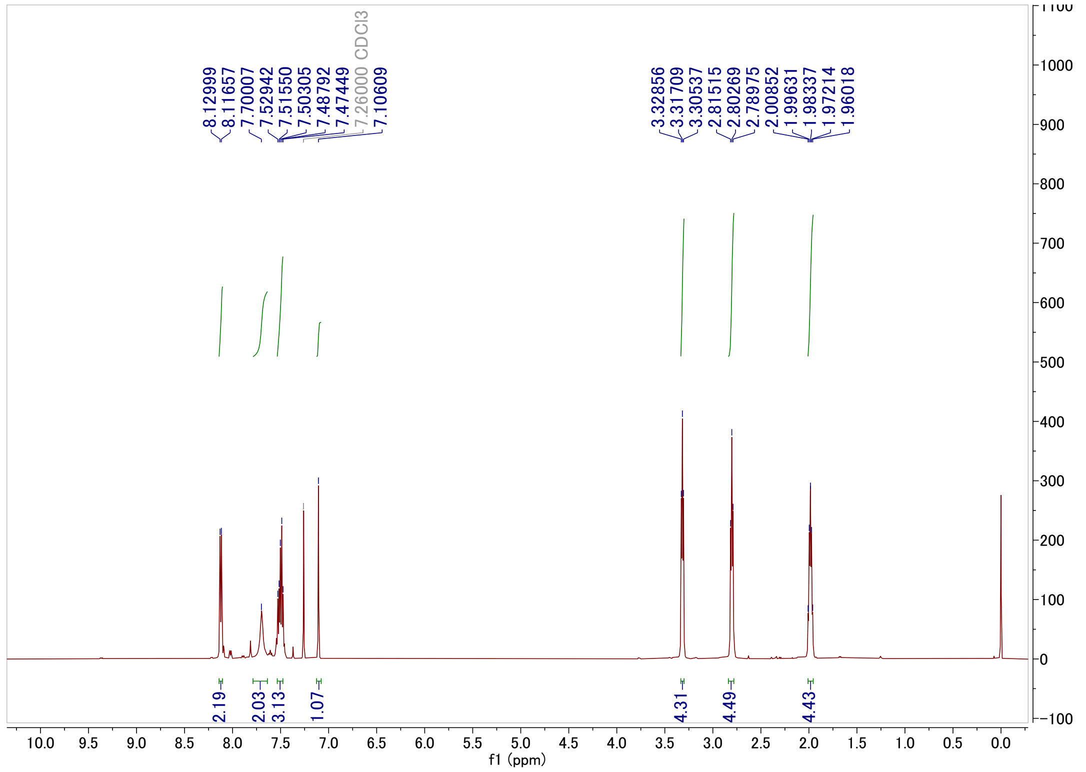

13C-NMR

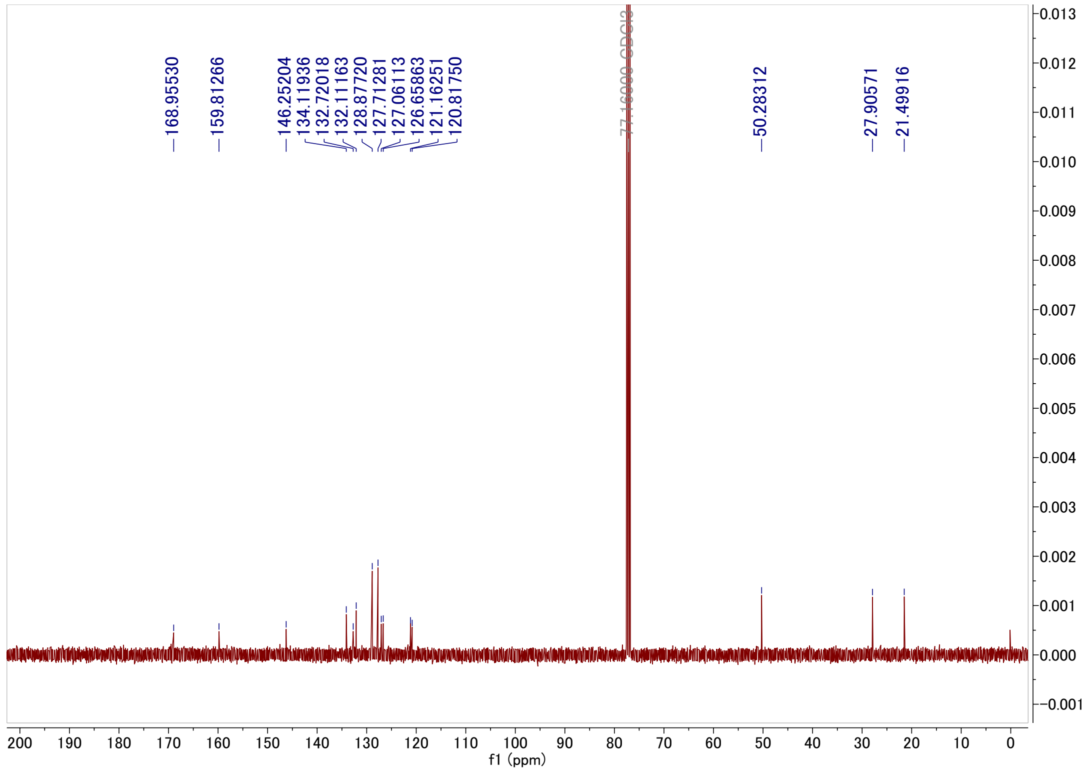

ESI-TOF-MS

Analysis Info

		Acquisition Date	2022/11/16 9:11:56	
Analysis Name	D:\Data\ofcbunseki\irai\2022\yuasa_lab\sadai\211116\SDIO28-apci-000003.d			
Method	APCI_II_pos_microTOF.m	Operator	BDAL@DE	
Sample Name	SDIO28-apci-	Instrument / Ser#	micrOTOF	213750.10321
Comment				

Acquisition Parameter

Source Type	APCI	Ion Polarity	Positive	Set Nebulizer	2.0 Bar
Focus	Not active			Set Dry Heater	200 ‹C
Scan Begin	50 m/z	Set Capillary	4500 V	Set Dry Gas	3.5 l/min
Scan End	1800 m/z	Set End Plate Offset	-500 V	Set Divert Valve	Waste

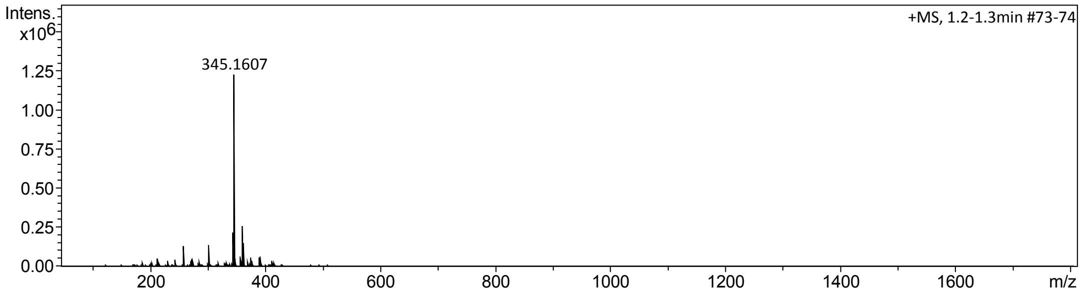

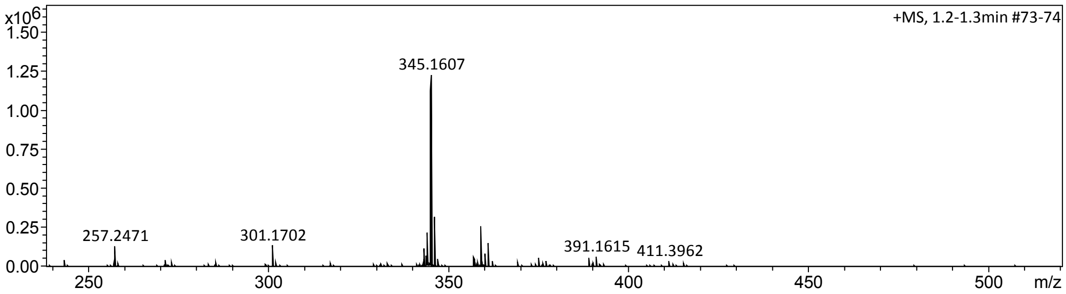

DMAB-Ph

1H-NMR

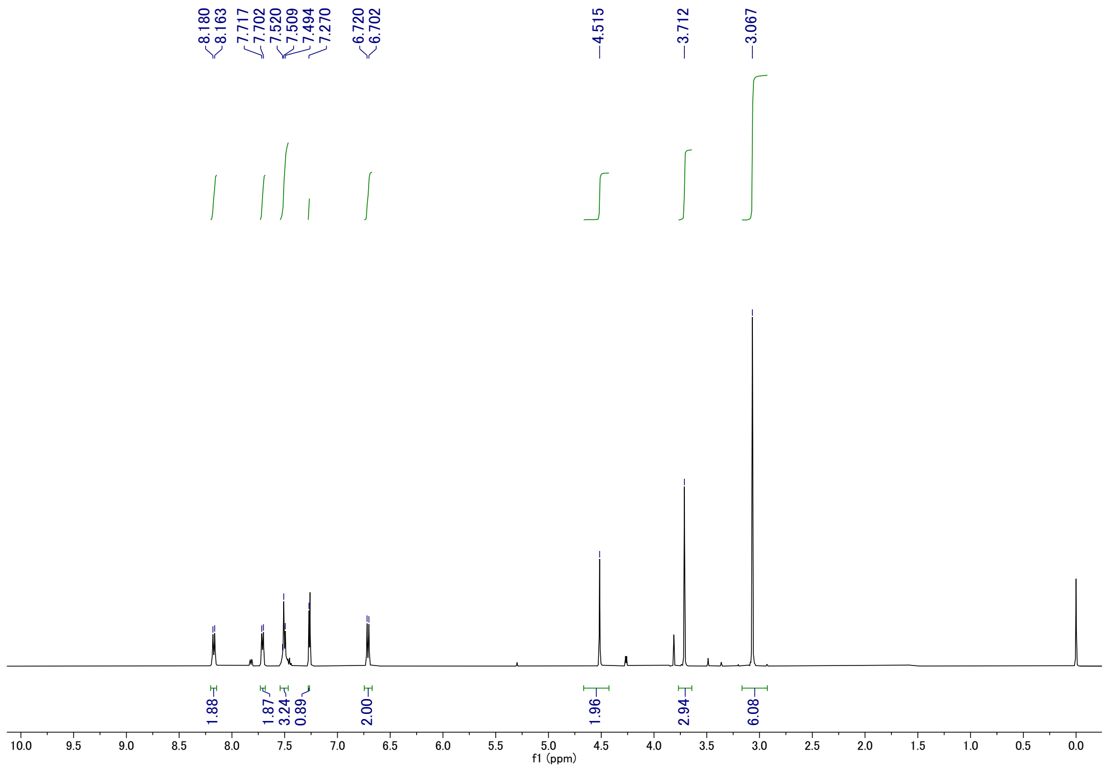

13C-NMR

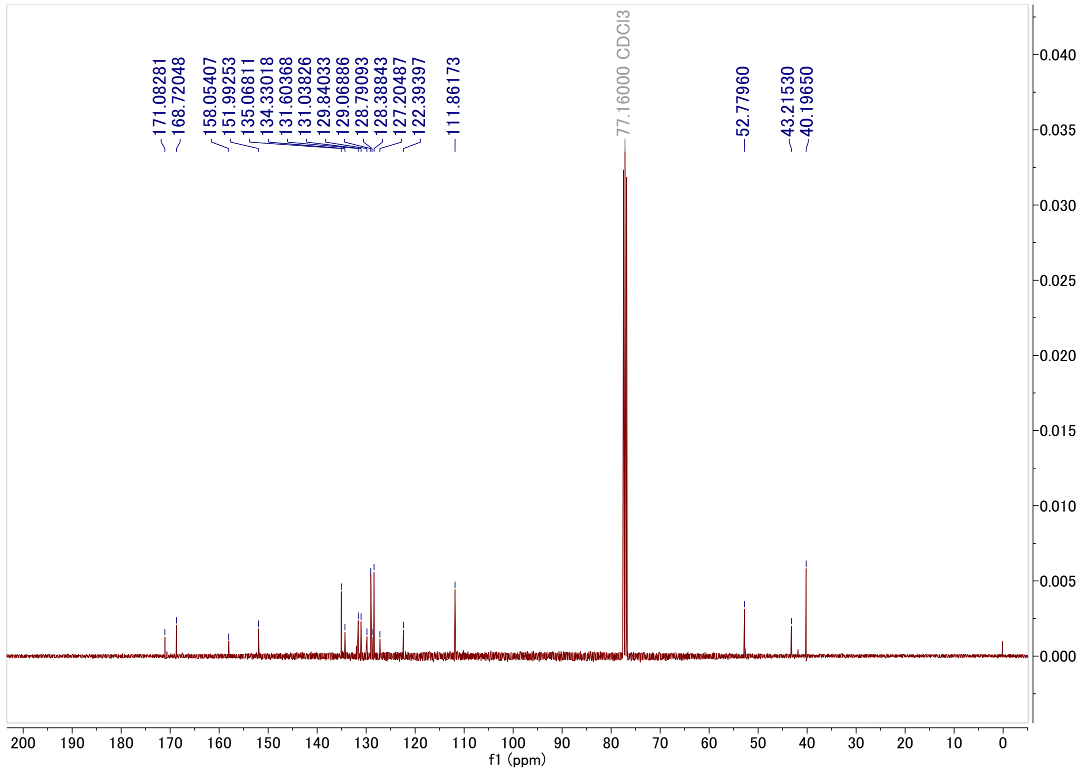

ESI-TOF-MS

Analysis Info

Analysis Name	D:\Data\ofcbunseki\irai\2022\yuasa_lab\hida\230216\NOG002-000001.d	Acquisition Date	2023/02/15 8:56:21
Method	esi_posi_low.m	Operator	BDAL@DE
Sample Name	NOG002-	Instrument / Ser#	micrOTOF 213750.10321
Comment			

Acquisition Parameter

Source Type	ESI	Ion Polarity	Positive	Set Nebulizer	0.3 Bar
Focus	Not active			Set Dry Heater	180 ‹C
Scan Begin	50 m/z	Set Capillary	4500 V	Set Dry Gas	4.0 l/min
Scan End	1000 m/z	Set End Plate Offset	-500 V	Set Divert Valve	Waste

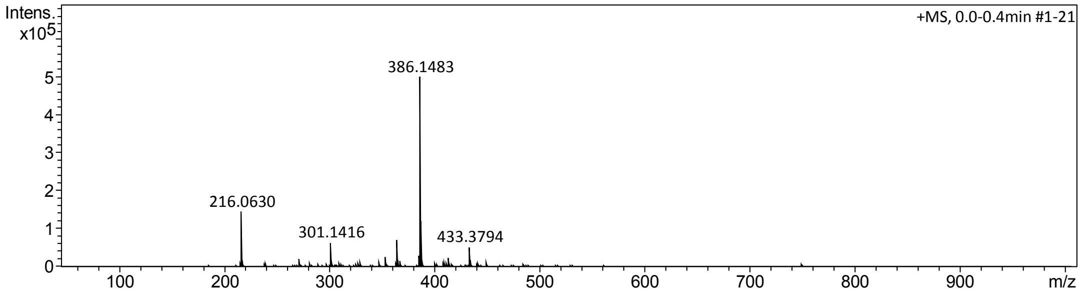

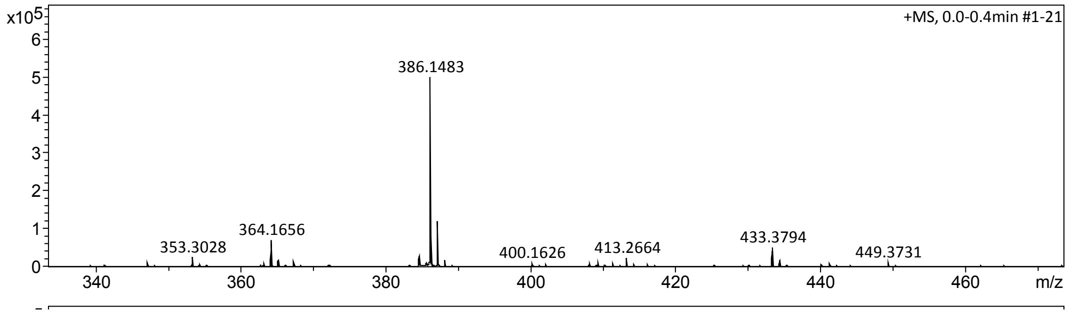

PyrroB-Ph

1H-NMR

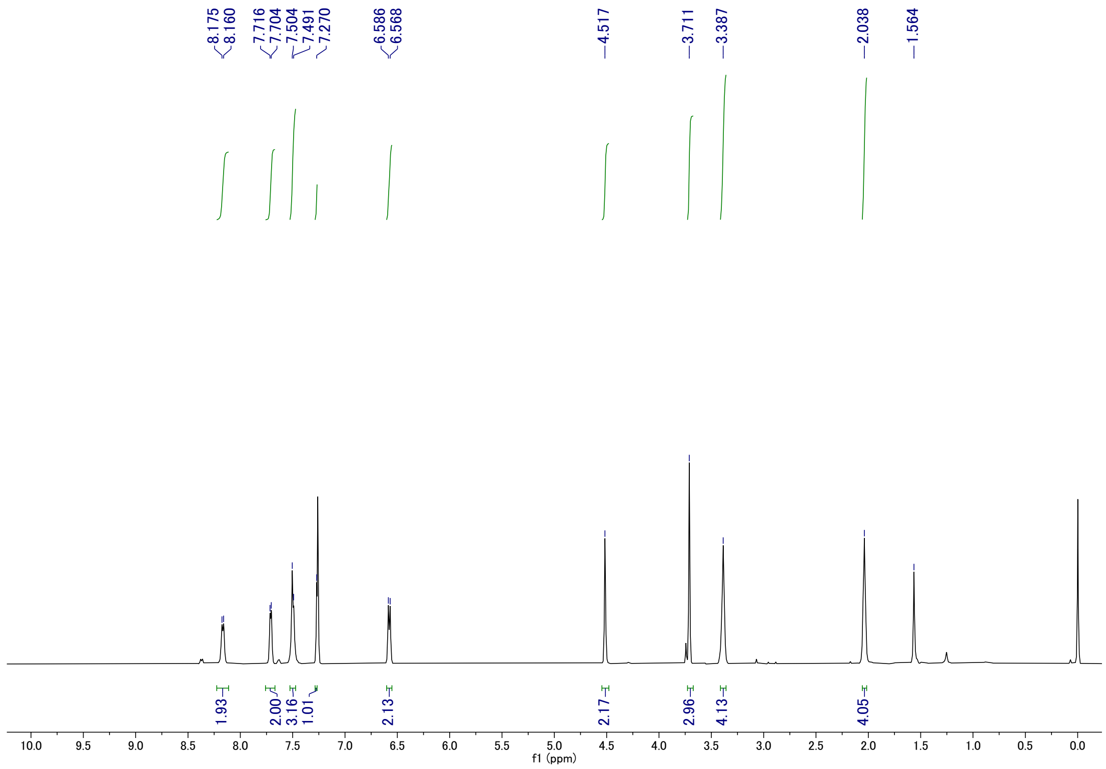

13C-NMR

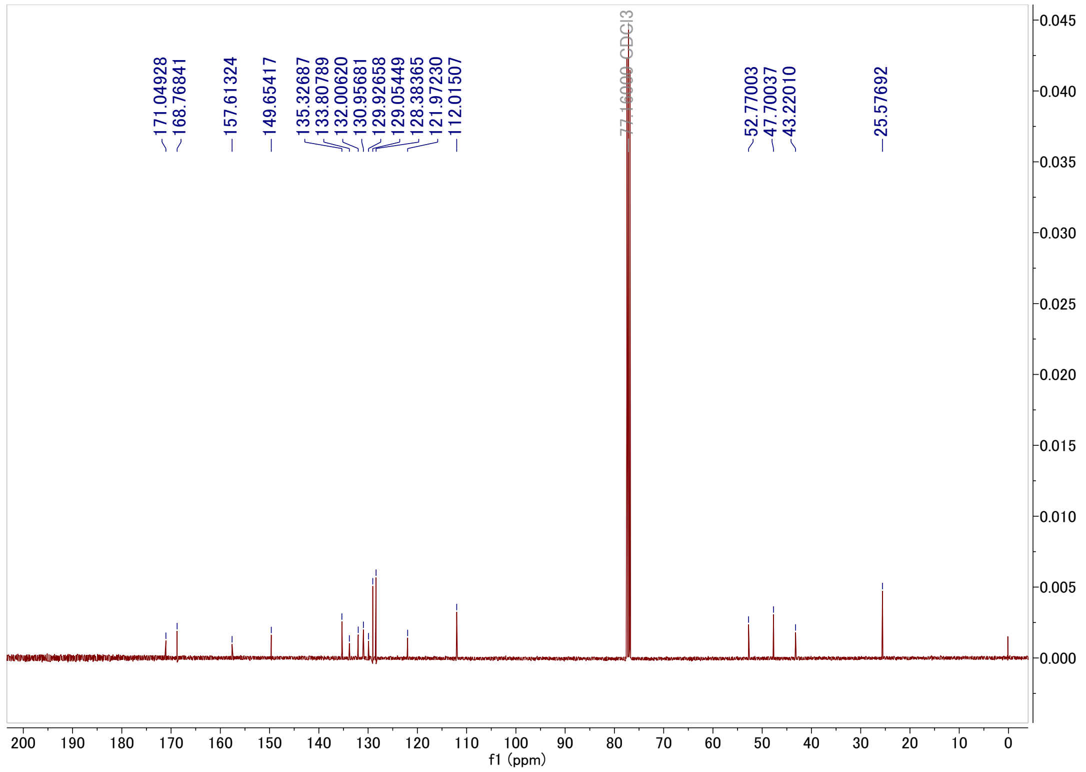

ESI-TOF-MS

Analysis Info

Analysis Name	D:\Data\ofcbunseki\irai\2022\yuasa_lab\sadai\211116\HID120-000001.d	Acquisition Date	2022/11/15 10:33:58
Method	esi_posi_low.m	Operator	BDAL@DE
Sample Name	HID120-	Instrument / Ser#	micrOTOF 213750.10321
Comment			

Acquisition Parameter

Source Type	ESI	Ion Polarity	Positive	Set Nebulizer	0.3 Bar
Focus	Not active			Set Dry Heater	180 ‹C
Scan Begin	50 m/z	Set Capillary	4500 V	Set Dry Gas	4.0 l/min
Scan End	1200 m/z	Set End Plate Offset	-500 V	Set Divert Valve	Waste

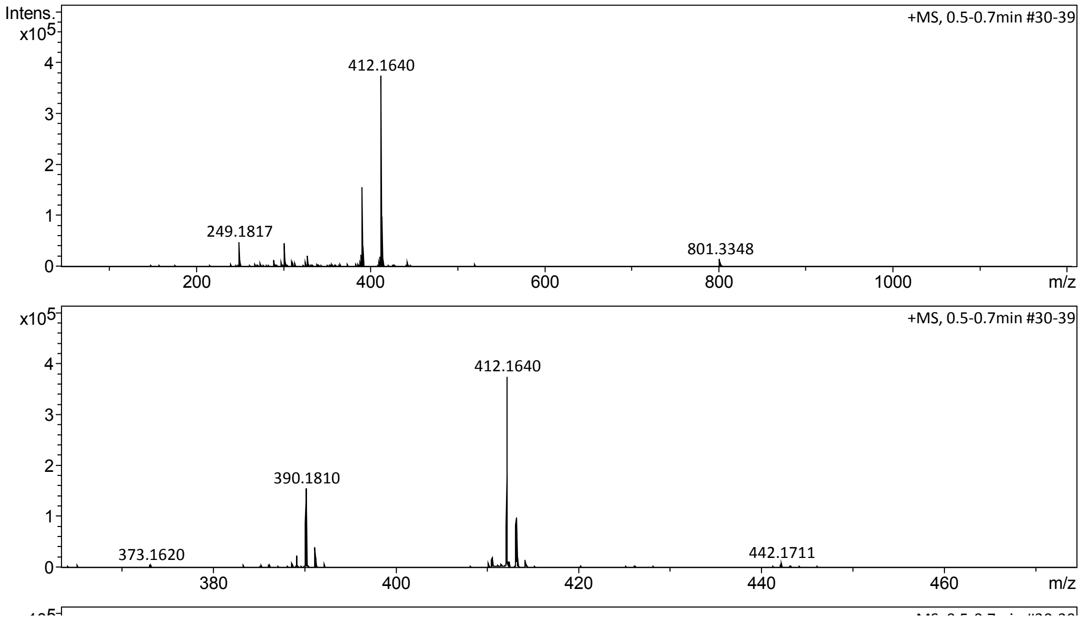

Julo-Ph

1H-NMR

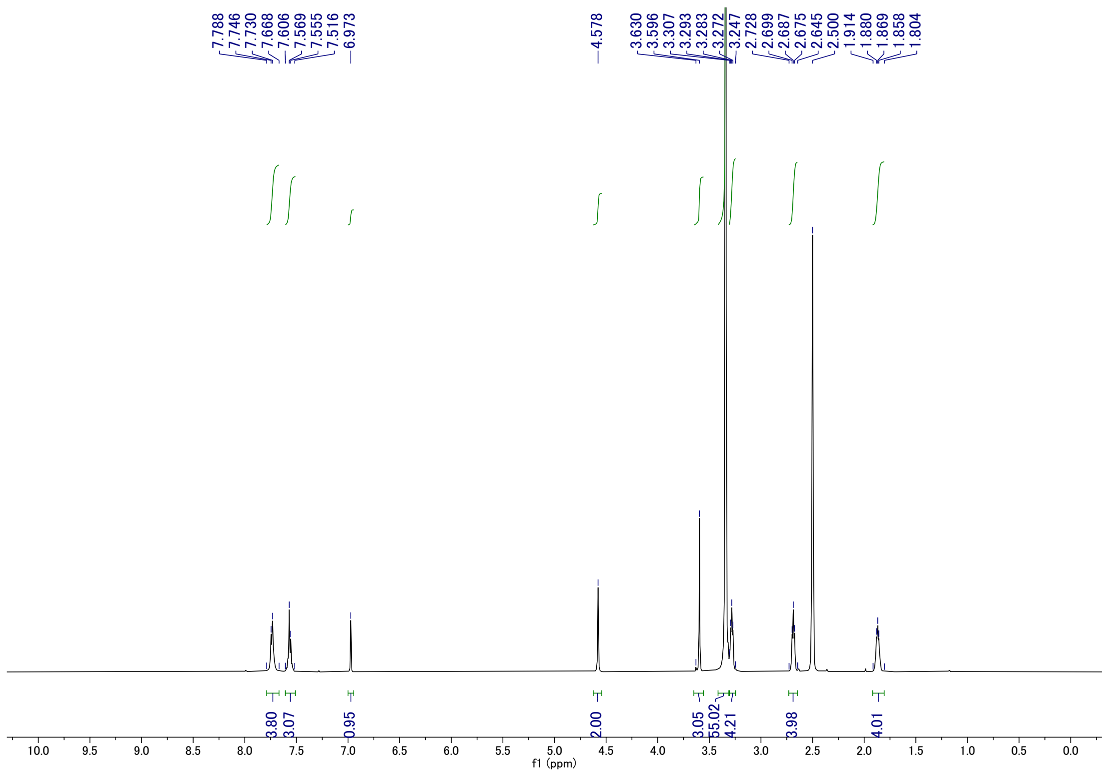

13C-NMR

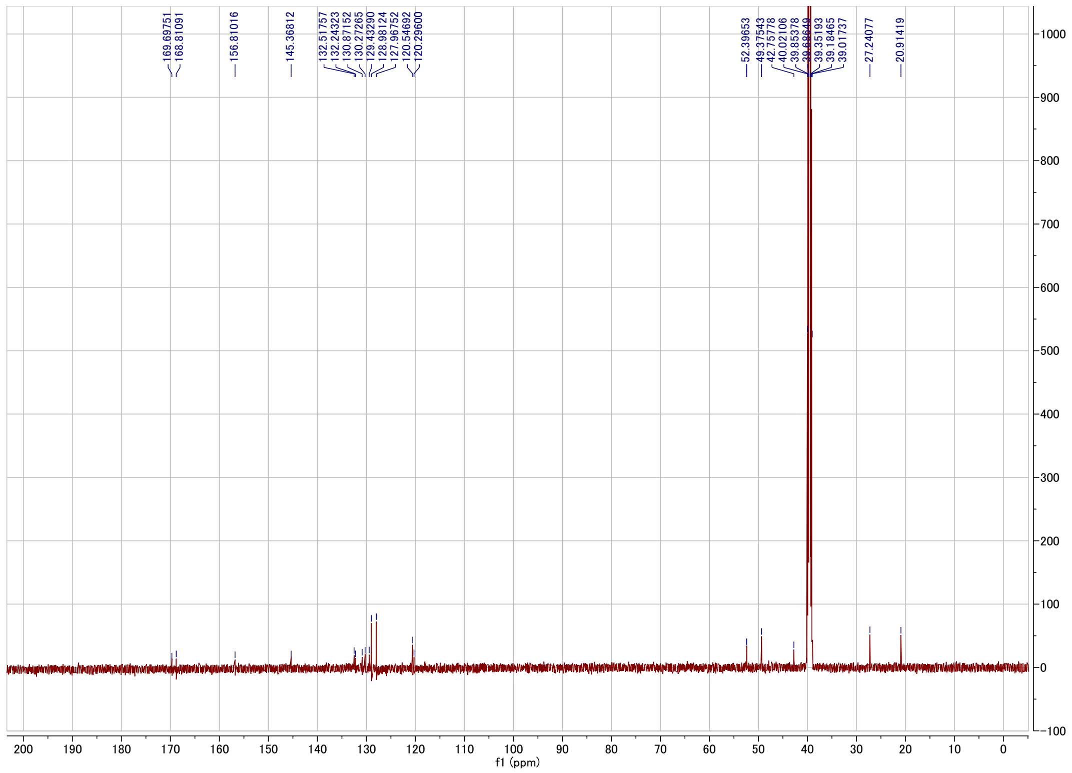

ESI-TOF-MS

**Analysis Info**		Acquisition Date	2022/11/15 10:14:37	
Analysis Name	D:\Data\ofcbunseki\irai\2022\yuasa_lab\sadai\211116\HID124-000001.d			
Method	esi_posi_low.m	Operator	BDAL@DE	
Sample Name	HID124-	Instrument / Ser#	micrOTOF	213750.10321
Comment				

**Acquisition Parameter**					
Source Type	ESI	Ion Polarity	Positive	Set Nebulizer	0.3 Bar
Focus	Not active			Set Dry Heater	180 ‹C
Scan Begin	50 m/z	Set Capillary	4500 V	Set Dry Gas	4.0 l/min
Scan End	1200 m/z	Set End Plate Offset	-500 V	Set Divert Valve	Waste

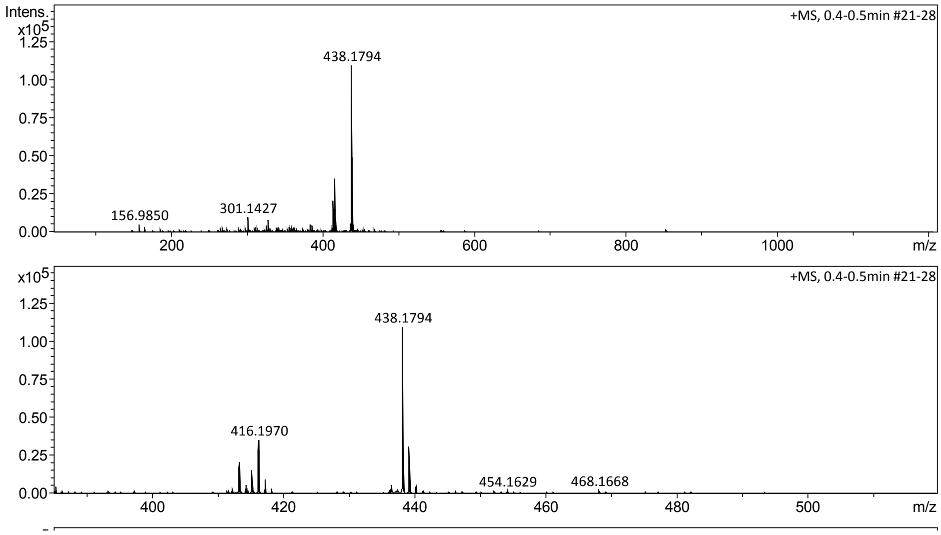

Julo-Me-COOH

ESI-TOF-MS

**Analysis Info**		Acquisition Date	2021/09/01 9:12:40	
Analysis Name	D:\Data\yuasa_lab\hida\210902\TT2058-000001.d			
Method	esi_posi_low.m	Operator	BDAL@DE	
Sample Name	TT2058-	Instrument / Ser#	micrOTOF	213750.10321
Comment				

**Acquisition Parameter**					
Source Type	ESI	Ion Polarity	Negative	Set Nebulizer	0.3 Bar
Focus	Not active			Set Dry Heater	180 ‹C
Scan Begin	50 m/z	Set Capillary	2600 V	Set Dry Gas	4.0 l/min
Scan End	1000 m/z	Set End Plate Offset	-500 V	Set Divert Valve	Waste

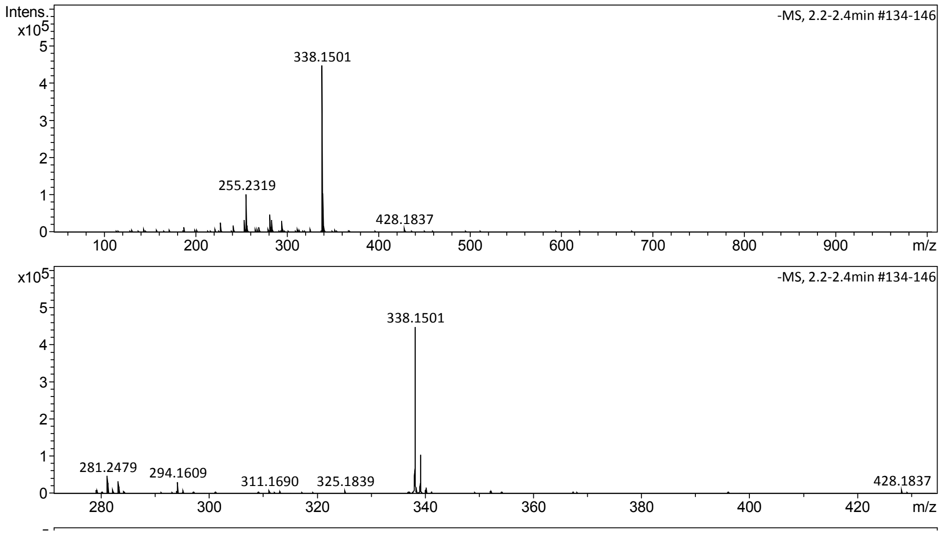

PyrroB-Ph-COOH

ESI-TOF-MS

Analysis Info

		Acquisition Date	2023/12/12 9:57:29
Analysis Name	D:\Data\ofcbunseki\irai\2023\yuasa_lab\sadai\231213\SDI059-000001.d		
Method	esi_posi_low.m	Operator	BDAL@DE
Sample Name	SDI059-	Instrument	micrOTOF 213750.10321
Comment			

Acquisition Parameter

Source Type	ESI	Ion Polarity	Negative	Set Nebulizer	0.3 Bar
Focus	Not active			Set Dry Heater	180 ‹C
Scan Begin	50 m/z	Set Capillary	2600 V	Set Dry Gas	4.0 l/min
Scan End	1200 m/z	Set End Plate Offset	-500 V	Set Divert Valve	Waste

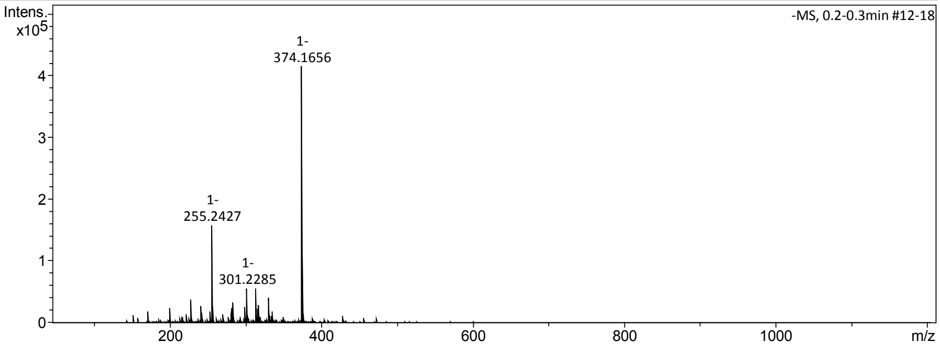

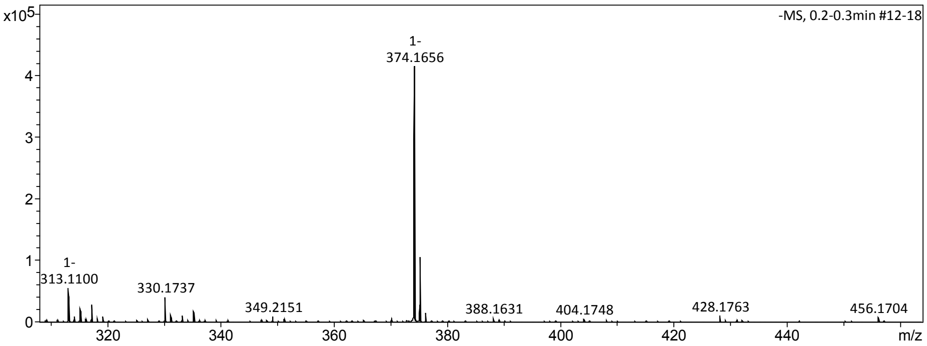

Julo-Ph-COOH

ESI-TOF-MS

Analysis Info

Analysis Name	D:\Data\ofcbunseki\irai\2022\yuasa_lab\sadai\211116\SDIO25-000001.d	Acquisition Date	2022/11/15 9:47:56
Method	esi_posi_low.m	Operator	BDAL@DE
Sample Name	SDIO25-	Instrument / Ser#	micrOTOF 213750.10321
Comment			

Acquisition Parameter

Source Type	ESI	Ion Polarity	Negative	Set Nebulizer	0.3 Bar
Focus	Not active			Set Dry Heater	180 ‹C
Scan Begin	50 m/z	Set Capillary	2600 V	Set Dry Gas	4.0 l/min
Scan End	1200 m/z	Set End Plate Offset	-500 V	Set Divert Valve	Waste

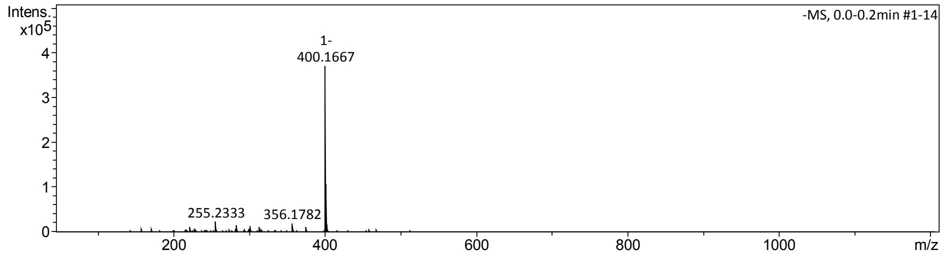

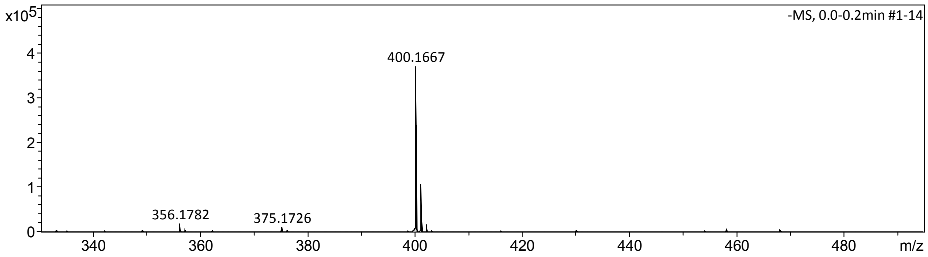

Glc-COOH

1H-NMR

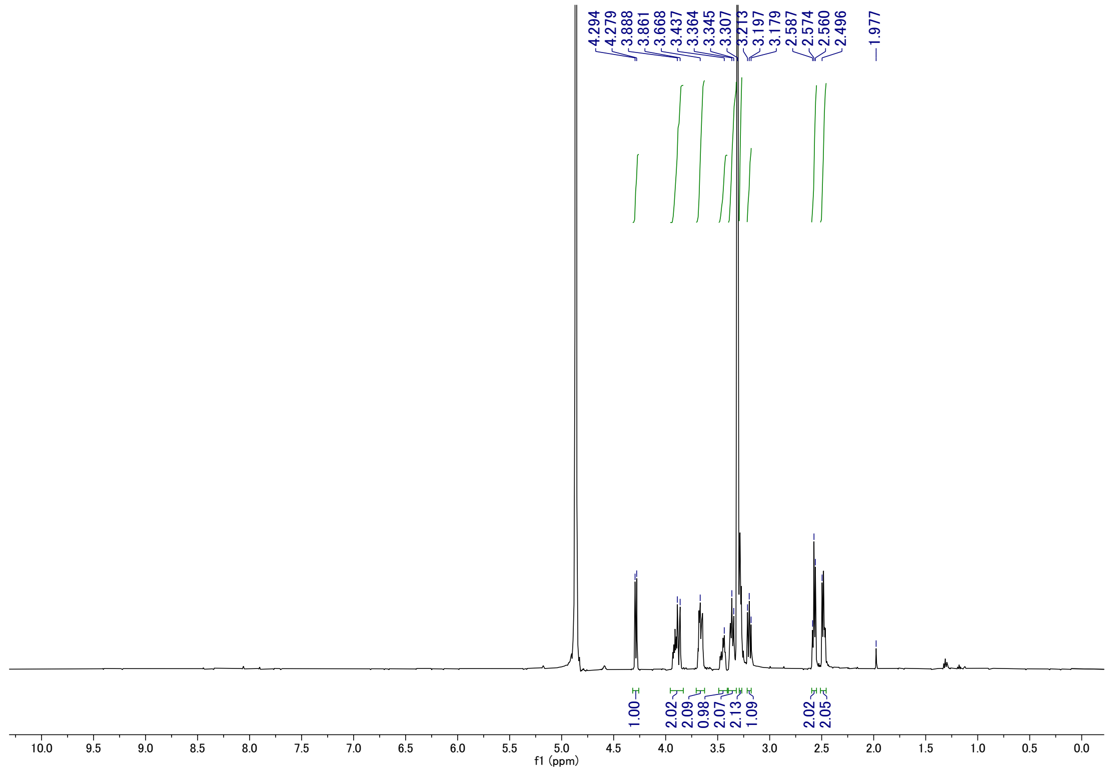

13C-NMR

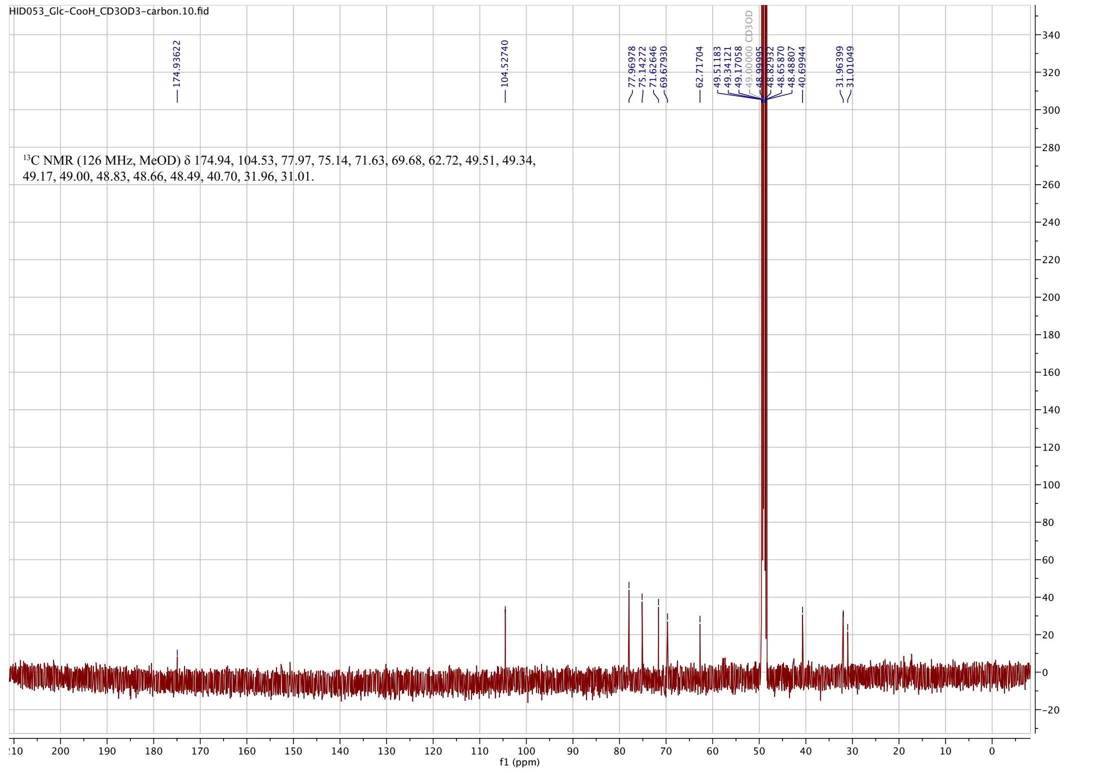

ESI-TOF-MS

TDCMAS ESI-TOF

Analysis Info

Analysis Name	D:\Data\yuasa_lab\hida\210804\HID053-000002.d	Acquisition Date	2021/08/04 9:43:26
Method	esi_posi_low.m	Operator	BDAL@DE
Sample Name	HID053-	Instrument / Ser#	micrOTOF 213750.10321
Comment			

Acquisition Parameter

Source Type	ESI	Ion Polarity	Positive	Set Nebulizer	0.3 Bar
Focus	Not active			Set Dry Heater	180 ‹C
Scan Begin	50 m/z	Set Capillary	4500 V	Set Dry Gas	4.0 l/min
Scan End	1000 m/z	Set End Plate Offset	-500 V	Set Divert Valve	Waste

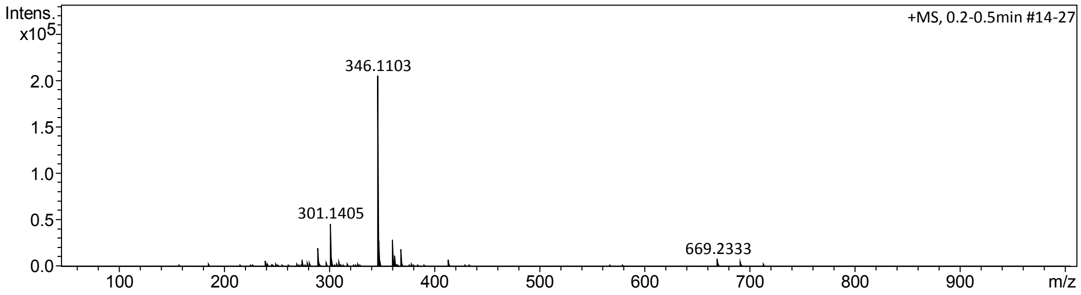

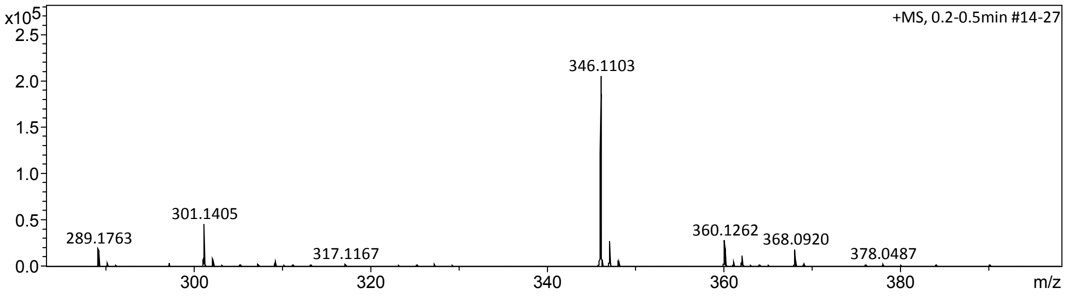

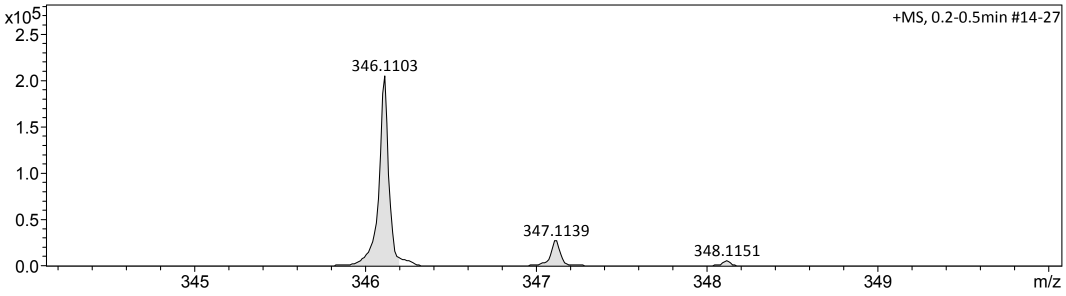

HPLC charts and ESI-TOF-MS spectra of probes

HPLC chart of Glc-Pep-Julo-Me (at 478 nm)

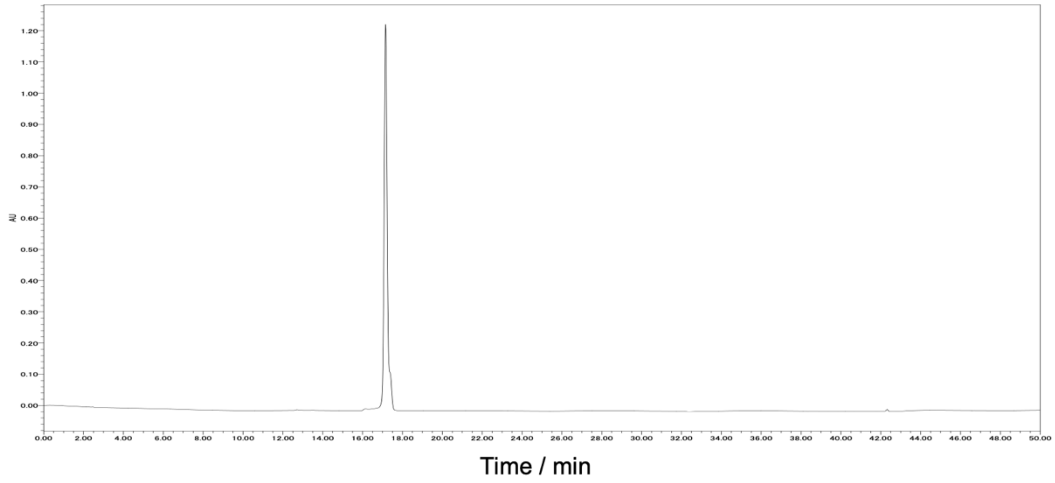

ESI-TOF-MS spectra of Glc-Pep-Julo-Me

TDCMAS ESI-TOF

Analysis Info

Analysis Name	D:\Data\yuasa_lab\sadai\220621\SDISP001-2-000001.d	Acquisition Date	2022/06/21 9:49:42
Method	esi_posi_low.m	Operator	BDAL@DE
Sample Name	SDISP001-2-	Instrument / Ser#	micrOTOF 213750.10321
Comment			

Acquisition Parameter

Source Type	ESI	Ion Polarity	Positive	Set Nebulizer	0.3 Bar
Focus	Not active			Set Dry Heater	180 ‹C
Scan Begin	50 m/z	Set Capillary	4500 V	Set Dry Gas	4.0 l/min
Scan End	2000 m/z	Set End Plate Offset	-500 V	Set Divert Valve	Waste

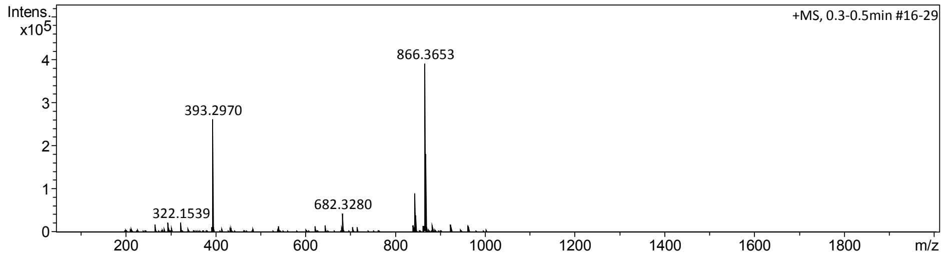

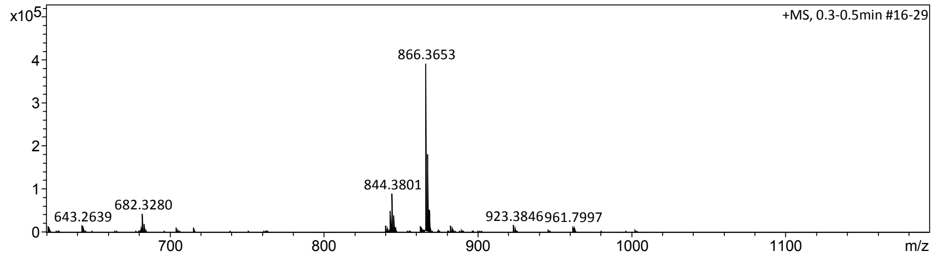

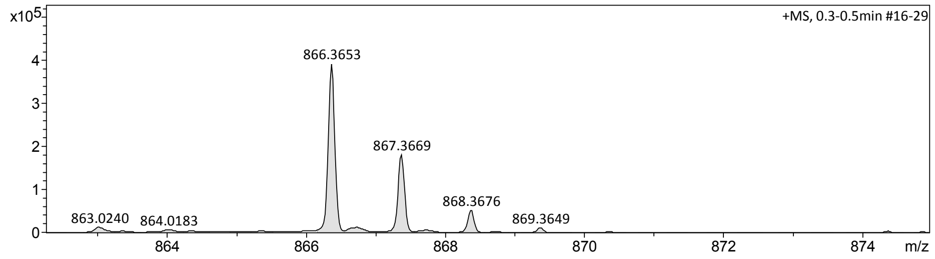

HPLC chart of Glc-Pep-PyrroB-Ph (at 480 nm)

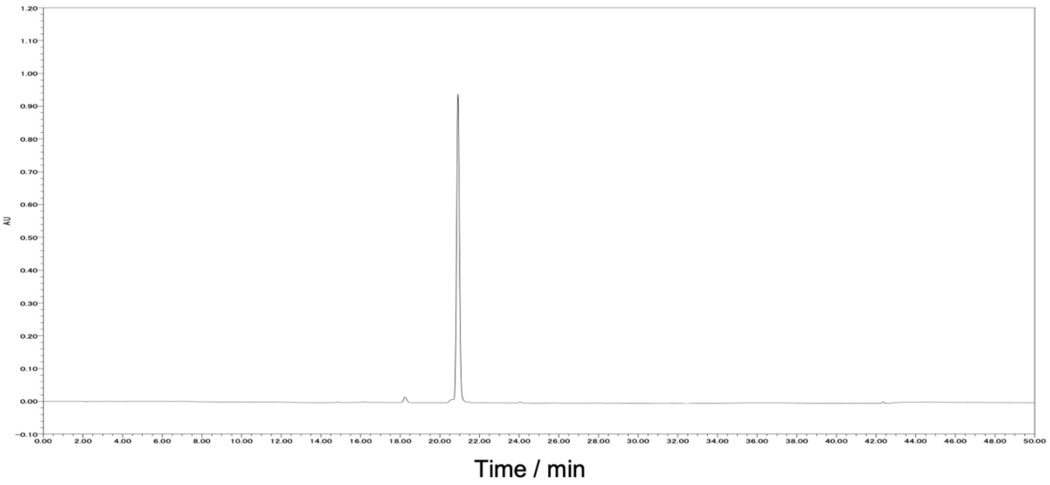

ESI-TOF-MS spectra of Glc-Pep-PyrroB-Ph

TDCMAS ESI-TOF

Analysis Info

		Acquisition Date	2024/12/12 9:21:19
Analysis Name	D:\Data\ofcbunseki\irai\2024\yuasa_lab\sadai\241213\Glc-Pep-Pyrro-Ph-000003.d		
Method	esi_posi_wide.m	Operator	BDAL@DE
Sample Name	Glc-Pep-Pyrro-Ph-	Instrument / Ser#	micrOTOF 213750.10321
Comment			

Acquisition Parameter

Source Type	ESI	Ion Polarity	Positive	Set Nebulizer	0.3 Bar
Focus	Not active			Set Dry Heater	180 ‹C
Scan Begin	50 m/z	Set Capillary	4500 V	Set Dry Gas	4.0 l/min
Scan End	2200 m/z	Set End Plate Offset	-500 V	Set Divert Valve	Waste

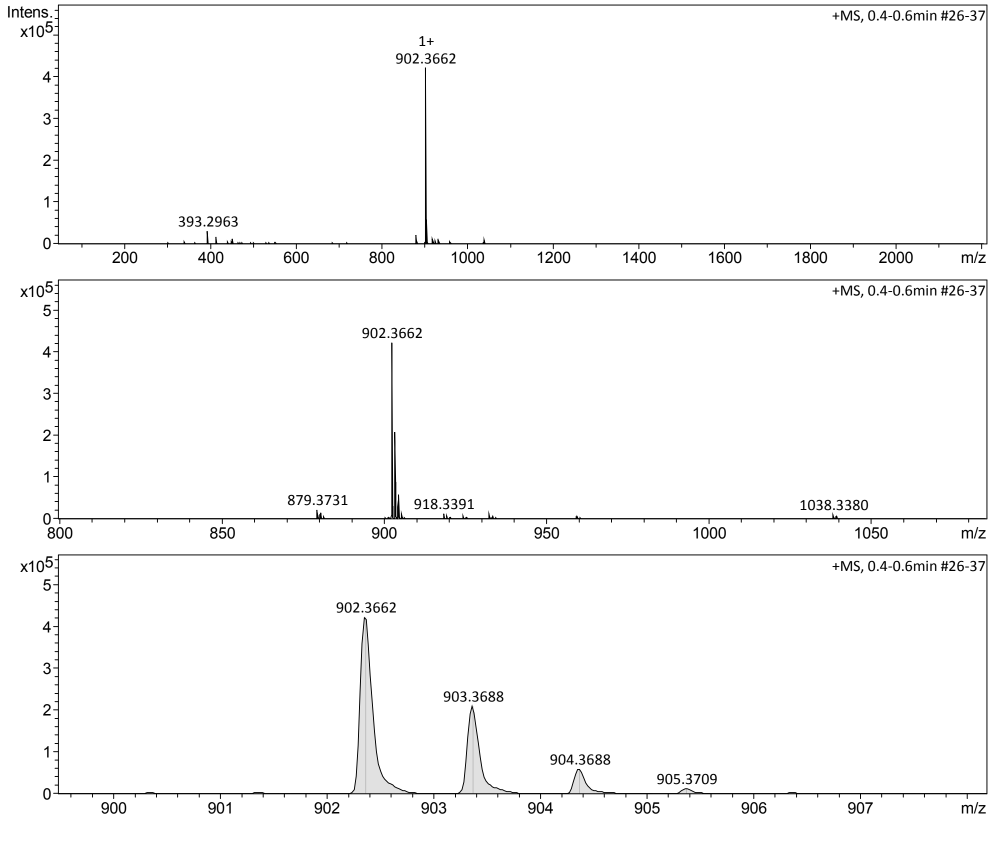

HPLC chart of Glc-Pep-Julo-Ph (at 500 nm)

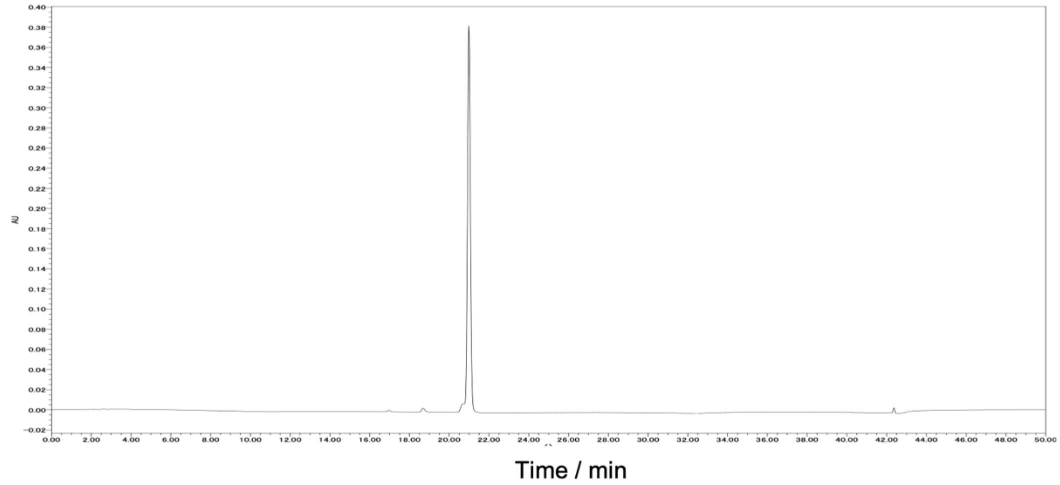

ESI-TOF-MS spectra of Glc-Pep-Julo-Ph

TDCMAS ESI-TOF

Analysis Info

Analysis Name	D:\Data\ofcbunseki\irai\2022\yuasa_lab\sadai\211116\SDISO003-3-000002.d	Acquisition Date	2022/11/15 11:02:21
Method	esi_posi_low.m	Operator	BDAL@DE
Sample Name	SDISO003-3-	Instrument / Ser#	micrOTOF 213750.10321
Comment			

Acquisition Parameter

Source Type	ESI	Ion Polarity	Positive	Set Nebulizer	0.3 Bar
Focus	Not active			Set Dry Heater	180 ‹C
Scan Begin	50 m/z	Set Capillary	4500 V	Set Dry Gas	4.0 l/min
Scan End	2200 m/z	Set End Plate Offset	-500 V	Set Divert Valve	Waste

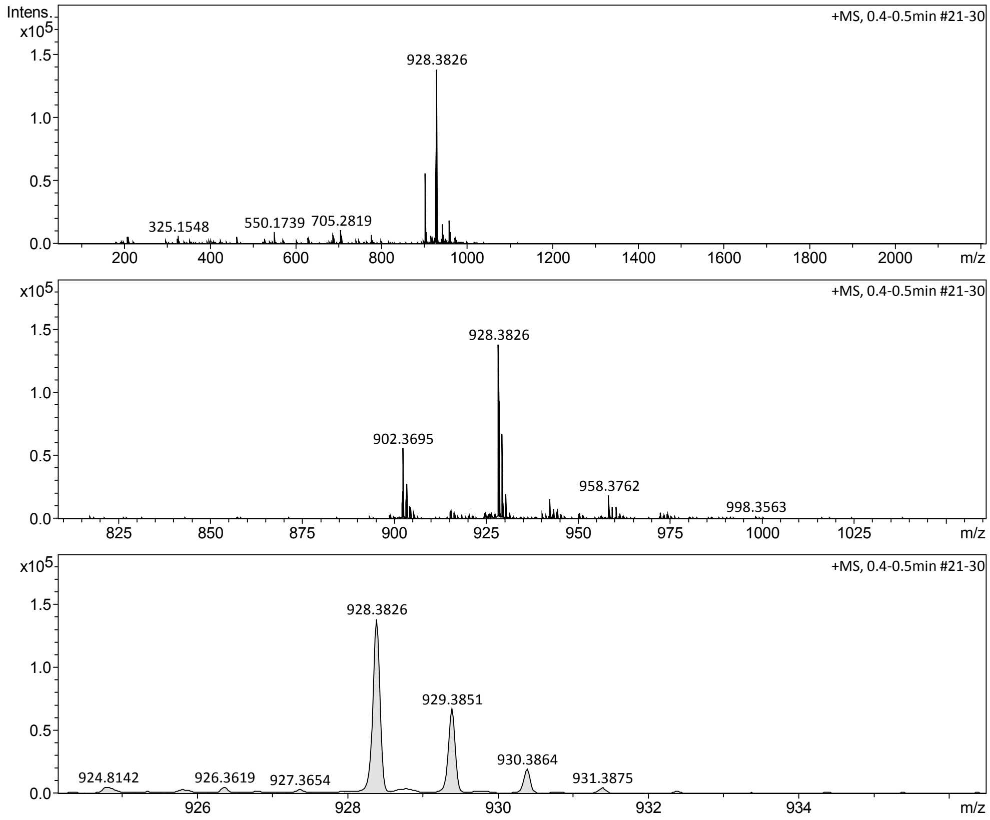

HPLC chart of GlcN-Julo-Me (at 470 nm)

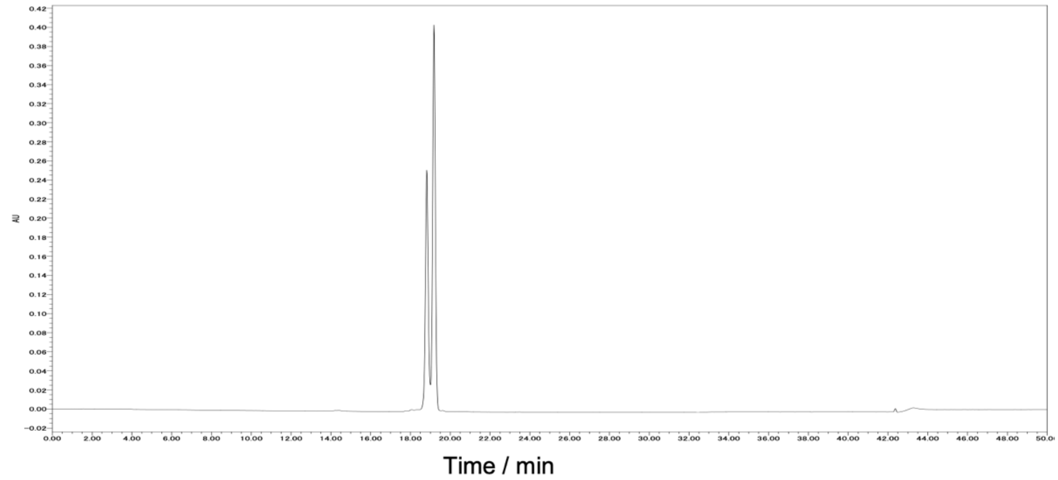

ESI-TOF-MS spectra of GlcN-Julo-Me

TDCMAS ESI-TOF

Analysis Info

		Acquisition Date	2024/07/04 11:53:10
Analysis Name	D:\Data\ofcbunseki\irai\2024\yuasa_lab\sadai\240705\GlcN-Julo-Me-000004.d		
Method	esi_posi_low.m	Operator	BDAL@DE
Sample Name	GlcN-Julo-Me-	Instrument / Ser#	micrOTOF 213750.10321
Comment			

Acquisition Parameter

Source Type	ESI	Ion Polarity	Positive	Set Nebulizer	0.3 Bar
Focus	Not active			Set Dry Heater	180 ‹C
Scan Begin	50 m/z	Set Capillary	4500 V	Set Dry Gas	4.0 l/min
Scan End	1300 m/z	Set End Plate Offset	-500 V	Set Divert Valve	Waste

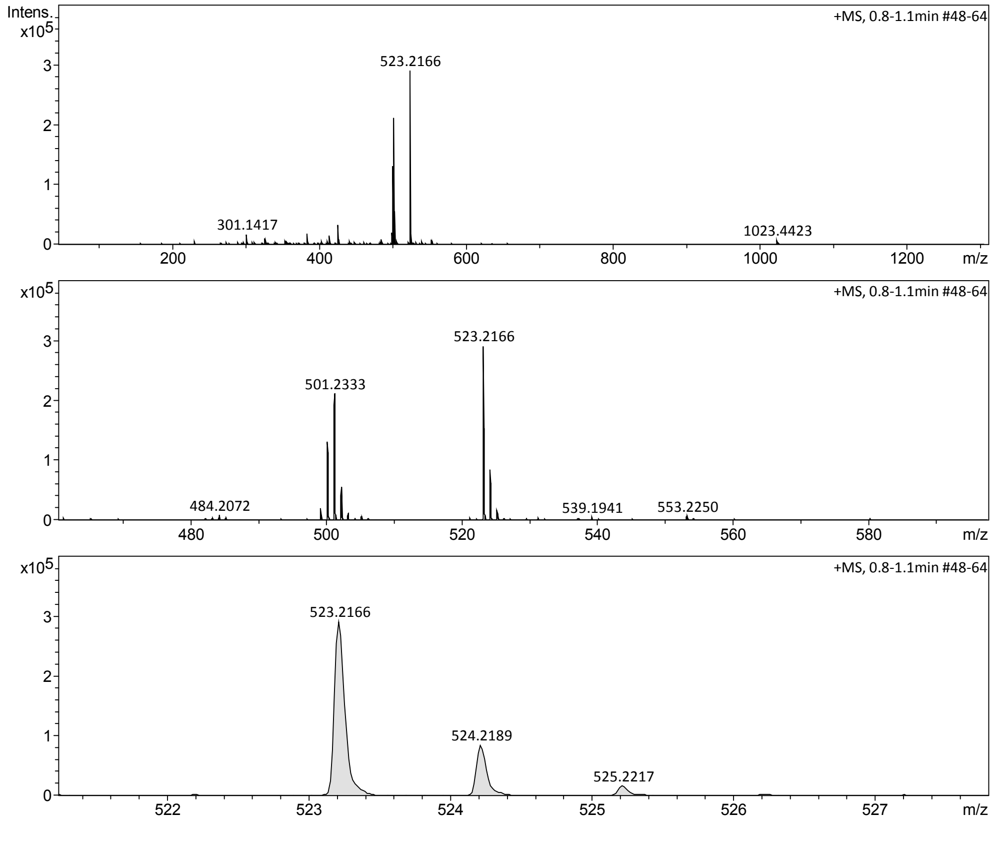

HPLC chart of GlcN-PyrroB-Ph (at 468 nm)

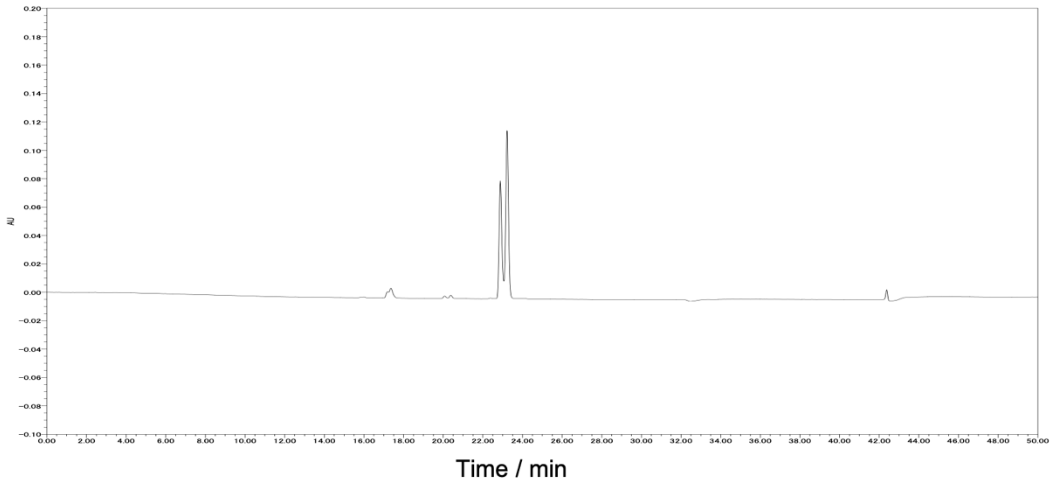

ESI-TOF-MS spectra of GlcN-PyrroB-Ph

TDCMAS ESI-TOF

Analysis Info

Analysis Name	D:\Data\ofcbunseki\irai\2024\yuasa_lab\sadai\241213\GlcN-Pyrro-Ph-000002.d	Acquisition Date	2024/12/12 9:34:13
Method	esi_posi_low.m	Operator	BDAL@DE
Sample Name	GlcN-Pyrro-Ph-	Instrument / Ser#	micrOTOF 213750.10321
Comment			

Acquisition Parameter

Source Type	ESI	Ion Polarity	Positive	Set Nebulizer	0.3 Bar
Focus	Not active			Set Dry Heater	180 ‹C
Scan Begin	50 m/z	Set Capillary	4500 V	Set Dry Gas	4.0 l/min
Scan End	1300 m/z	Set End Plate Offset	-500 V	Set Divert Valve	Waste

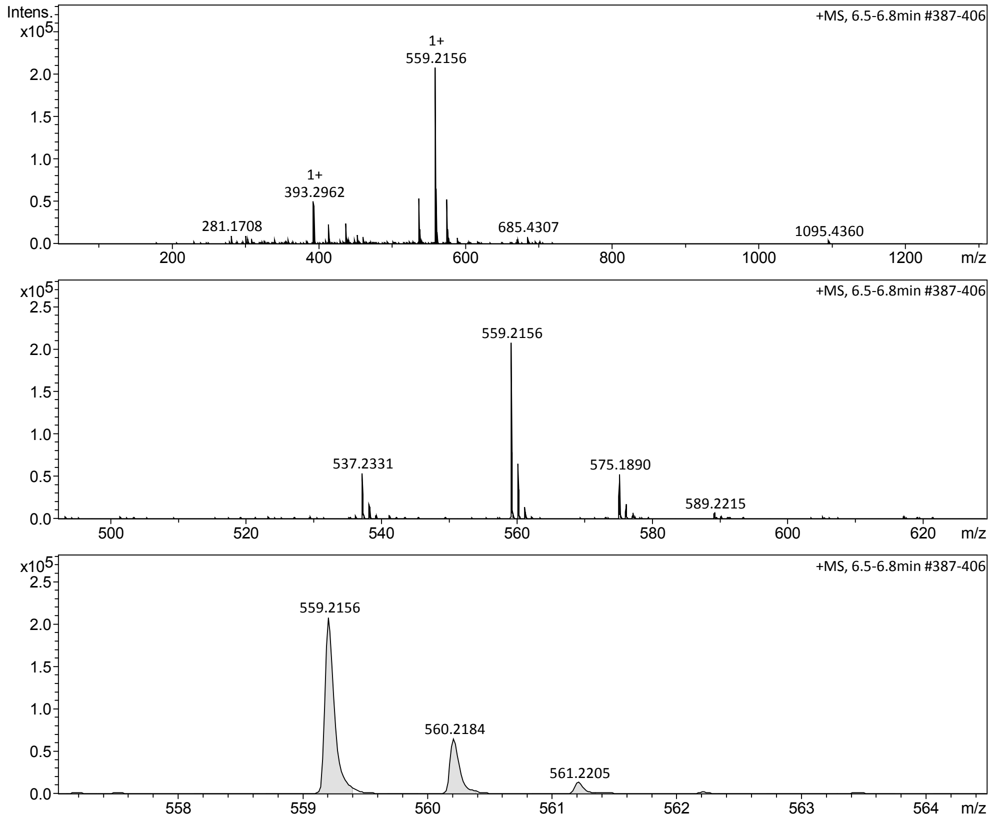

HPLC chart of GlcN-Julo-Ph (500 nm)

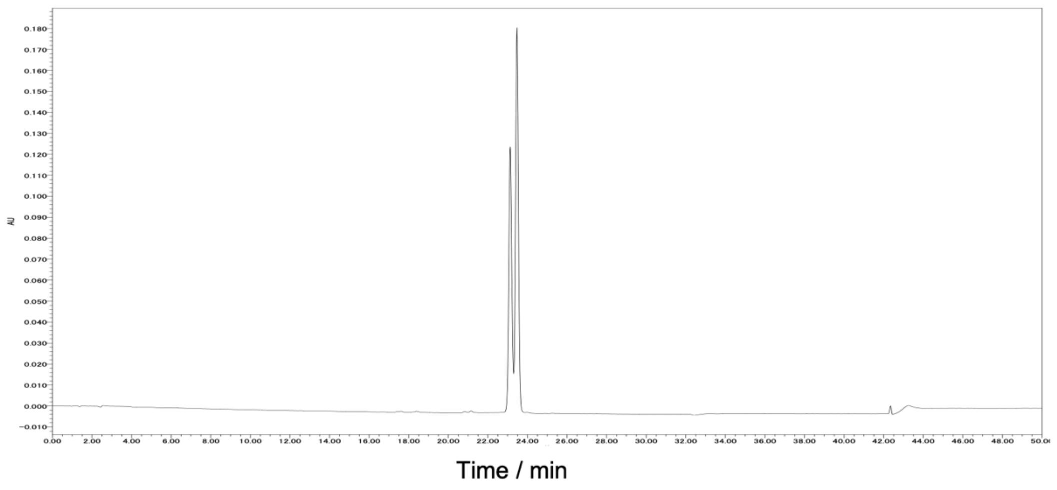

ESI-TOF-MS spectra of GlcN-Julo-Ph

TDCMAS ESI-TOF

Analysis Info

Analysis Name	D:\Data\ofcbunseki\irai\2023\yuasa_lab\sadai\231213\SDI062-2nd-000002.d	Acquisition Date	2023/12/12 10:29:02
Method	esi_posi_low.m	Operator	BDAL@DE
Sample Name	SDI062-2nd-	Instrument / Ser#	micrOTOF 213750.10321
Comment			

Acquisition Parameter

Source Type	ESI	Ion Polarity	Positive	Set Nebulizer	0.3 Bar
Focus	Not active			Set Dry Heater	180 ‹C
Scan Begin	50 m/z	Set Capillary	4500 V	Set Dry Gas	4.0 l/min
Scan End	1400 m/z	Set End Plate Offset	-500 V	Set Divert Valve	Waste

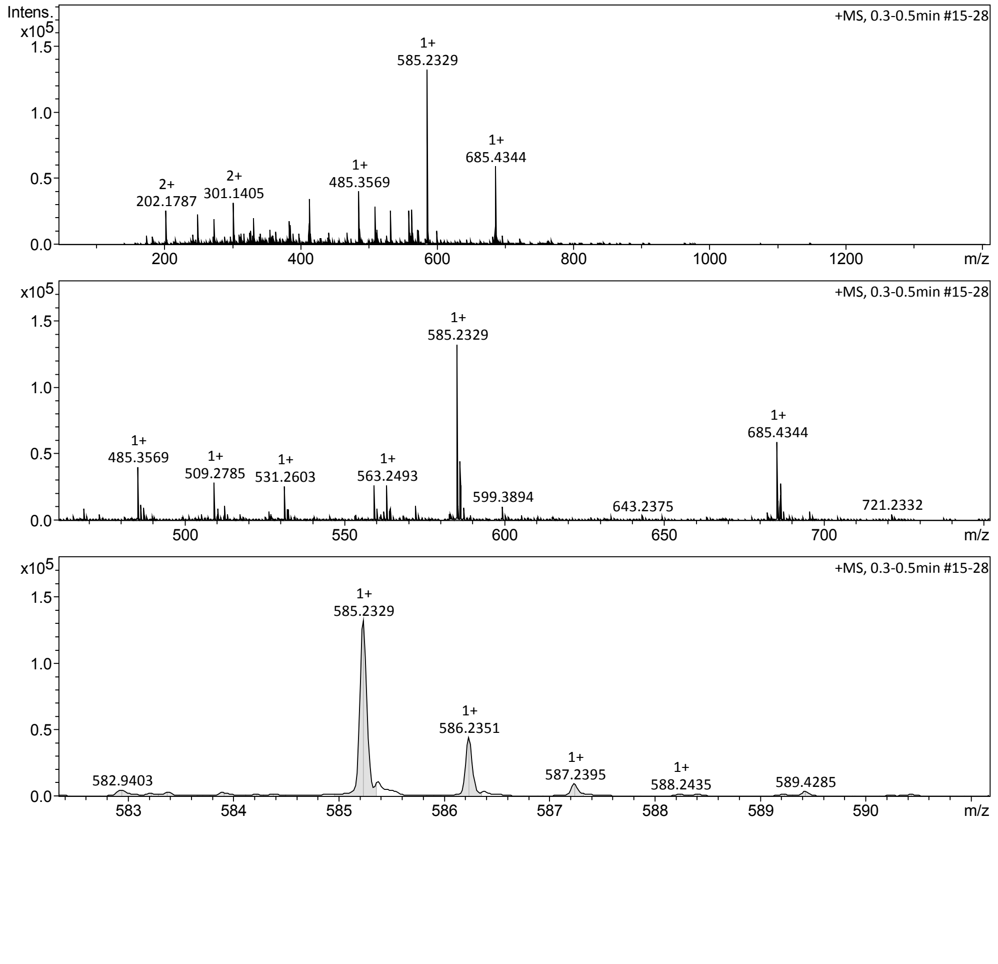

3. References

[1] J. Kowalik, A. Baldridge, L. Tolbert, *Synthesis* **2010**, *2010*, 2424-2436.

[2] T. B. Clark, M. E. Orr, D. C. Flynn, T. Goodson, *J. Phys. Chem. C* **2011**, *115*, 7331-7338.

[3] P. Magdolen, M. Mečiarová, Š. Toma, *Tetrahedron* **2001**, *57*, 4781-4785.

[4] D. Su, C. Zhan, Y. Xiang, W. Wang, C. Li, Q. Zhou, D. Dong, S. Xiao, *J. Mater. Chem. A* **2025**, *13*, 3903-3912.

[5] V. Yarlagadda, M. M. Konai, G. B. Manjunath, C. Ghosh, J. Haldar, *J. Antibiot.* **2015**, *68*, 302-312.

[6] Gaussian 16, Revision B.01, M. J. Frisch, G. W. Trucks, H. B. Schlegel, G. E. Scuseria, M. A. Robb, J. R. Cheeseman, G. Scalmani, V. Barone, G. A. Petersson, H. Nakatsuji, X. Li, M. Caricato, A. V. Marenich, J. Bloino, B. G. Janesko, R. Gomperts, B. Mennucci, H. P. Hratchian, J. V. Ortiz, A. F. Izmaylov, J. L. Sonnenberg, D. Williams-Young, F. Ding, F. Lipparini, F. Egidi, J. Goings, B. Peng, A. Petrone, T. Henderson, D. Ranasinghe, V. G. Zakrzewski, J. Gao, N. Rega, G. Zheng, W. Liang, M. Hada, M. Ehara, K. Toyota, R. Fukuda, J. Hasegawa, M. Ishida, T. Nakajima, Y. Honda, O. Kitao, H. Nakai, T. Vreven, K. Throssell, J. J. A. Montgomery, J. E. Peralta, F. Ogliaro, M. J. Bearpark, J. J. Heyd, E. N. Brothers, K. N. Kudin, V. N. Staroverov, T. A. Keith, R. Kobayashi, J. Normand, K. Raghavachari, A. P. Rendell, J. C. Burant, S. S. Iyengar, J. Tomasi, M. Cossi, J. M. Millam, M. Klene, C. Adamo, R. Cammi, J. W. Ochterski, R. L. Martin, K. Morokuma, O. Farkas, J. B. Foresman, D. J. Fox, Gaussian, Inc., Wallingford CT, **2016**.
